# Oncogenic State and Cell Identity Combinatorially Dictate the Susceptibility of Cells within Glioma Development Hierarchy to IGF1R Targeting

**DOI:** 10.1002/advs.202001724

**Published:** 2020-10-01

**Authors:** Anhao Tian, Bo Kang, Baizhou Li, Biying Qiu, Wenhong Jiang, Fangjie Shao, Qingqing Gao, Rui Liu, Chengwei Cai, Rui Jing, Wei Wang, Pengxiang Chen, Qinghui Liang, Lili Bao, Jianghong Man, Yan Wang, Yu Shi, Jin Li, Minmin Yang, Lisha Wang, Jianmin Zhang, Simon Hippenmeyer, Junming Zhu, Xiuwu Bian, Ying‐Jie Wang, Chong Liu

**Affiliations:** ^1^ Department of Neurosurgery of the Second Affiliated Hospital Zhejiang University School of Medicine Hangzhou 310058 China; ^2^ Department of Pathology and Pathophysiology Zhejiang University School of Medicine Hangzhou 310058 China; ^3^ State Key Laboratory for Diagnosis and Treatment of Infectious Diseases Collaborative Innovation Center for Diagnosis and Treatment of Infectious Diseases The First Affiliated Hospital Zhejiang University School of Medicine Hangzhou 310058 China; ^4^ Department of Pathology of the Second Affiliated Hospital Zhejiang University School of Medicine Hangzhou 310058 China; ^5^ College of Basic Medical Science Inner Mongolia Medical University Hohhot 010059 China; ^6^ State Key Laboratory of Proteomics Institute of Basic Medical Sciences National Center of Biomedical Analysis Beijing 100850 China; ^7^ Department of Pathology Institute of Pathology and Southwest Cancer Center Southwest Hospital Third Military Medical University Chongqing 400038 China; ^8^ PharmaBlock Sciences (Nanjing), Inc. Nanjing 210032 China; ^9^ Institute of Science and Technology Austria Am Campus 1 Klosterneuburg 3400 Austria; ^10^ School of Brain Science and Brain Medicine NHC and CAMS Key Laboratory of Medical Neurobiology Zhejiang University School of Medicine Hangzhou 310058 China

**Keywords:** cancer cell of origin, glioma, IGF1R, lineage tracing, mosaic analysis with double markers (MADM), neural stem cells (NSCs), oligodendrocyte precursor cells (OPCs)

## Abstract

Glioblastoma is the most malignant cancer in the brain and currently incurable. It is urgent to identify effective targets for this lethal disease. Inhibition of such targets should suppress the growth of cancer cells and, ideally also precancerous cells for early prevention, but minimally affect their normal counterparts. Using genetic mouse models with neural stem cells (NSCs) or oligodendrocyte precursor cells (OPCs) as the cells‐of‐origin/mutation, it is shown that the susceptibility of cells within the development hierarchy of glioma to the knockout of insulin‐like growth factor I receptor (IGF1R) is determined not only by their oncogenic states, but also by their cell identities/states. Knockout of IGF1R selectively disrupts the growth of mutant and transformed, but not normal OPCs, or NSCs. The desirable outcome of IGF1R knockout on cell growth requires the mutant cells to commit to the OPC identity regardless of its development hierarchical status. At the molecular level, oncogenic mutations reprogram the cellular network of OPCs and force them to depend more on IGF1R for their growth. A new‐generation brain‐penetrable, orally available IGF1R inhibitor harnessing tumor OPCs in the brain is also developed. The findings reveal the cellular window of IGF1R targeting and establish IGF1R as an effective target for the prevention and treatment of glioblastoma.

## Graphical abstract

Using genetic mouse models with NSCs or OPCs as the glioma cells‐of‐origin/mutation, it is shown that desirable outcomes of IGF1R knockout on cell growth require mutant cells with the development hierarchy to commit to the OPC identity. The findings herein identify the cellular window of IGF1R targeting, and firmly establish IGF1R as an effective target for glioma prevention and treatment.

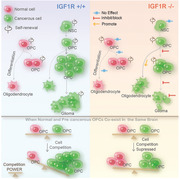

## 1 Introduction

Adult gliomas are the most common cancers of the central nervous system (CNS).^[^ ^1^ ^]^ Despite many years of efforts, the prognosis of malignant gliomas, particularly the most advanced one, glioblastoma multiforme (GBM), remains dismal. Therefore, it is urgent to identify effective targets for this lethal disease. Inactivation of a promising druggable target should suppress the growth of cancer cells and, ideally also pretransforming cells for the purpose of prevention, but minimally affects normal cells. The cell‐of‐origin can be considered as a rational “normal” reference, as it shares significantly more key features with pretransforming and cancer cells than with irrelevant cell types.

Following this concept, two studies identified Bone marrow X‐linked kinase (BMX) selectively suppressed the self‐renewal of human glioma tumor initiating cells (TICs) but not affected either cultured neural stem cells (NSCs) or astrocytes,^[^ ^2^, ^3^ ^]^ both of which have been considered as the putative cells‐of‐origin for human GBMs. The specificity of glioma TICs to BMX targeting is likely due to the absence of BMX in normal brain cells.^[^ ^2^, ^3^ ^]^ BMX may represent a unique case as many more genes should be shared by both cancer cells and their cells‐of‐origin. Indeed, by using a genetic mouse glioma model with NSCs as the putative cell‐of‐origin,^[^ ^4^ ^]^ Shi et al. identified Gboxin, an inhibitor targeting mitochondrial F_0_F_1_ ATP synthase,^[^ ^5^ ^]^ selectively suppressed primary mouse and human glioblastoma cells but not that of mouse embryonic fibroblasts (MEFs) or astrocytes. However, when using the NSCs as reference, Gboxin exhibited certain toxicity, although not as prominent as glioma TICs. Moreover, in both studies, it is unknown whether the identified genes function as specific targets to suppress spontaneous gliomagenesis in an in vivo context.

Oligodendrocyte precursor cells (OPCs) are a major type of glial cells in the brain, and, similar to NSCs, can self‐renew throughout their life.^[^ ^6^ ^]^ Using genetic mouse models combined with lineage tracing techniques, many laboratories including ours showed that OPCs may function as the putative cell‐of‐origin, not only for GBM,^[^ ^4^, ^7^, ^8^, ^9^, ^10^ ^]^ but also for lower‐grade adult gliomas^[^ ^11^, ^12^ ^]^ and pediatric gliomas,^[^ ^13^ ^]^ highlighting that OPC‐originated malignancies represent an important pathologic entity in human gliomas. Thus, OPC‐originating genetic glioma models serve as a useful experimental model system to identify and validate therapeutic targets for glioma treatment and early intervention.

Insulin‐like growth factor (IGF) signaling has been associated with the pathogenesis of many human cancers, including gliomas.^[^ ^14^ ^]^ The increased expression of insulin‐like growth factor I receptor (IGF1R) and/or IGFs has been reported in glioma tissues and cerebrospinal fluid from GBM patients.^[^ ^15^, ^16^, ^17^, ^18^ ^]^ Interference with IGF1R function was shown to inhibit the growth of glioma cell lines in vitro and in preclinical mouse models.^[^ ^15^, ^16^, ^19^, ^20^, ^21^, ^22^ ^]^ However, it remains unclear whether all cells in the development hierarchy of glioma respond to IGF1R targeting equally, considering the highly heterogeneous features of GBMs in a native tumor. It is an important topic given that the therapies targeting IGF1R have been undergoing in clinical trials.^[^ ^23^ ^]^ In addition, it is unknown theoretically whether targeting IGF1R could sufficiently suppress the initiation and progression of glioma in the native in vivo context, and whether it is specific to tumor cells given the presumption that IGF1R is widely expressed in many cells including normal NSCs and OPCs. In this study, we addressed these important questions by using genetic autochthonous mouse glioma models, in combination with ex vivo cell culture system as well as patient‐derived xenografts (PDX) models.

## 2 Results

### 2.1 Single‐Cell Transcriptomics Reveals a Lineage Development Hierarchy in Adult OPC‐Derived Gliomas

**Figure 1:**
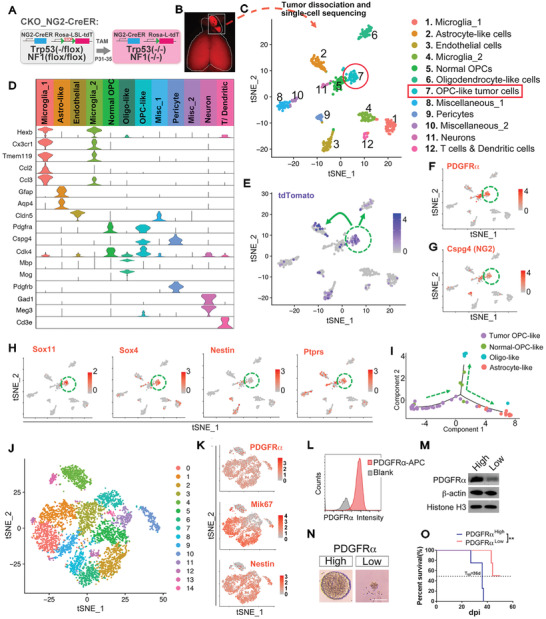
Single‐cell transcriptomics and the grafting assay reveal the TIC function of tumor OPCs in adult OPC‐derived gliomas. A) The genetic configuration of the CKO_NG2‐Cre^ER^ mouse model. B) The gross image of a tumor brain used for the scRNA‐seq in (C). C) The tSNE map of all sequenced cells from the tumor in (B). The cluster containing tumor OPCs is circled. D) The Violin plots of some marker genes from the clusters defined in (C). Same color code is used in (C) and (D). E–H) Projection of the lineage or marker genes as indicated onto the tSNE map in (C). The cluster containing tumor OPCs is circled. I) The pseudo‐time plot of all tdTomato^+^ cells from Clusters 2, 5, 6, and 7. The presumed differentiation directions are marked as dotted arrow lines. J) The tSNE map of a mouse CKO_NG2‐Cre^ER^ glioma cell line from the scRNA‐seq data. Distinct clustered are marked by different colors. K) Projection of the marker genes as indicated onto the tSNE map in (J). L) The representative FACS plot showing the expression of PDGFR*α* on mouse tumor OPCs. M) The Western blots validate the expression of FACS‐sorted glioma cells based on their surface PDGFR*α* expression. N) The in vitro sphere assay of tumor OPCs based on their surface PDGFR*α* expression sorted by FACS. Scale bar: 100 µm. O) The survival curves of mice grafted with PDGFR*α* high/low tumor OPC fractions, *N* = 4 mice for each group, ::*p* < 0.01.

In order to delineate the intratumoral hierarchy of OPC‐originated gliomas, we resorted to a genetic mouse model (referred to as CKO_NG2‐Cre^ER^, **Figure** **1**A) and performed droplet‐based single‐cell RNA sequencing (scRNA‐seq).^[^ ^8^ ^]^ In the CKO_NG2‐Cre^ER^ model, tumor suppressors *Trp53* and *NF1* were specifically inactivated in adult OPCs using a temporally controllable OPC‐specific NG2‐Cre^ER^ transgene. In addition, a Cre‐recombinase‐dependent lineage‐tracing reporter tdTomato was incorporated to visualize all initially generated mutant cells and their progeny (including tumors developed at the later stage, as shown in Figure 1B). In agreement with previous reports,^[^ ^4^, ^8^ ^]^ we confirmed that in this model the NG2‐Cre^ER^ transgene solely labeled OPCs and nonneural lineage pericytes, but not other neuroglia, or neural stem cells (NSCs) residing in all brain germinal zones examined (including the subventricular zones from the lateral, third, and fourth ventricles as wells as the hippocampus, data not shown). Therefore, the CKO_NG2‐Cre^ER^ model represents an in vivo experimental system to study the biology of gliomas with OPCs as the cell‐of‐origin.

scRNA‐seq for the cells dissociated from a CKO_NG2‐Cre^ER^ tumor (Figure 1B) identified 12 distinct clusters, visualized using t‐distributed stochastic neighbor embedding (t‐SNE, Figure 1C). Referring to known cellular markers in the brain,^[^ ^24^ ^]^ we could identify the cell identities/states of ten clusters (Figure 1D; Figure S1, Supporting Information), including two for OPCs (C5 and C7), two for microglia (C1 and C4), one each for astrocytes (C2), endothelial cells (C3), oligodendrocytes (C6), pericytes (C9), neurons (C11), and T/dendritic cells (C12). Projection of lineage marker tdTomato onto the t‐SNE map further revealed that 4 clusters (C2, C6, C7, and C9) were likely derived from NG2‐Cre^ER^ labeled cells and/or their progeny (Figure 1E). As expected, all hematopoietic lineage clusters were devoid of tdTomato expression, concordant to the current view that they are unrelated to the OPC or pericyte lineage. One interesting observation is that some endothelial cells in C3 exhibited detectable tdTomato signals (Figure 1E). Although it is surely possible that OPC‐derived glioma cells may transdifferentiate into endothelial cells, we could not exclude that the signals came from the contamination of the debris of pericytes or astrocytes, both of which tightly associated with endothelial cells in vivo. Future study is warranted to distinguish these possibilities.

In the previous work, we and others have identified a subpopulation of tumor cells in OPC‐derived glioma models by both bulk RNA sequencing and in situ immunofluorescence staining.^[^ ^4^, ^7^, ^8^, ^25^ ^]^ We could here assign this subpopulation to the cluster 7 on the t‐SNE map of the scRNA‐seq dataset based on their coexpression of both tdTomato and OPC makers including *PDGFRα* and *CSPG4* (*NG2*) (Figure 1D–G; Figure S1, Supporting Information). This cluster also enriched some well‐known stemness markers such as *Sox11*, *Sox4*, *Nestin*, *Ptprs*, and *Cdk4* (Figure 1H; Figure S1, Supporting Information), suggesting that they may function as the TICs in the tumor.

In the brain of the wild‐type (WT) or CKO model at the pretransforming stage, we could readily see tdTomato‐labeled OPCs and oligodendrocytes, but not astrocytes (data not show), consistent with the notion that OPCs can further develop along the oligodendrocyte lineage but not be able to transdifferentiate into astrocytes. Nevertheless, we detected the clusters of tumor cells exhibiting prominent features of oligodendrocytes and astrocytes (C6 and C2, respectively) that were also labeled by tdTomato (Figure 1E). Therefore, scRNA‐seq not only suggests that tumor OPCs, resembling their normal counterparts, can further differentiate into the oligodendrocyte state (C6), but also possesses the capacity to transdifferentiate into astrocyte‐like cells (C2). Interestingly, in parallel to C7, we also identified a second cluster (C5) that exhibited prominent OPC feature. Given that they were largely devoid of tdTomato, we reasoned that they represented normal OPCs in the tumor. Supporting this notion, this cluster of cells expressed less stemness markers compared to those in C7 (Figure 1D–H). Interestingly, C5 also harbored a small fraction of tdTomato^+^ cells, implicating that tumor OPCs could partially “differentiate” into more “normal” OPC‐like status (Figure 1E).

To further confirm the lineage relationship among tdTomato labeled neuroepithelium‐derived cells, we performed pseudo‐time analysis of all tdTomato^+^ cells from these four clusters (C2, C5, C6, and C7). The pseudo‐time plot in Figure 1I revealed that tumor OPCs, tumor oligo‐ and tumor astrocyte‐like cells occupied the three ends of the lineage trajectory, and there was evident continuity between tumor OPC and astrocyte‐like cells. Intriguingly, tumor‐OPCs appeared to undergo a more “normal” OPC‐like state before further differentiating into mature astrocyte‐ or oligodendrocyte‐like states. Therefore, scRNA‐seq suggests the existence of a development hierarchy in OPC‐derived mouse gliomas; and tumor cells representing stem cells/progenitors, astrocytes, OPCs, and oligodendrocytes coexist within this hierarchy, nicely concordant with a recent comprehensive scRNA‐seq analysis of human GBM.^[^ ^26^ ^]^

### 2.2 Tumor OPCs Function as TICs in Adult OPC‐Derived Gliomas

scRNA‐seq suggests that tumor OPCs in adult OPC‐originated gliomas may function as TICs given their strong stemness feature. Supporting this notion, our previous work using the immunopanning approach showed that PDGFR*α* ^+^ subpopulation enriched tumor cells in nonadherent culture.^[^ ^25^ ^]^ To further confirm whether these cells function as TICs in vivo, we performed scRNA‐seq on the cell line from the CKO_NG2‐Cre^ER^ tumor (Figure 1J). These results confirmed that both OPC‐like and non‐OPC like cells coexisted (Figure 1K). *PDGFRα* ^+^ tumor OPCs also enriched markers for division and stemness (Figure 1K and not shown). We sorted tumor OPCs by flow cytometry based on their surface expression of PDGFR*α* (Figure 1L,M), and validated that PDGFR*α* high expression fraction not only enriched the tumor cells growing in nonadherent culture condition (Figure 1N), but also those more effectively initiating secondary tumors after orthotopically grafted in the NOD‐SCID mouse brains (Figure 1O). Thus, we conclude that tumor cells resembling OPC features function as TICs in the OPC‐derived glioma model driven by *Trp53* and *NF1* mutations.

To further validate our mouse model work in human context, we analyzed the tumor tissues from GBM patients. Constant with the single cell sequencing and the immunohistological results from previous studies,^[^ ^9^, ^25^, ^27^, ^28^, ^29^, ^30^, ^31^, ^32^ ^]^ we found the presence of Olig2^+^ tumor OPCs in human GBMs regardless the molecular subtypes or mutations (Figures S2 and S3, Supporting Information). Similar to that in the mouse model, many tumor OPCs underwent active cell division, suggesting that they contributed to the proliferation pool in human GBM (Figures S2 and S3A, Supporting Information). We established primary GBM cell lines and found numerous tumor OPCs (Figure S3A, right panel, Supporting Information). Sorting of tumor OPCs based on their surface expression of PDGFR*α* showed that PDGFR*α* high fraction was the subpopulation to effectively grow in vitro (Figure S3B–D, Supporting Information) and generated tumors in vivo (Figure S3E,F, Supporting Information). This result nicely mirrors the mouse data and suggests that tumor OPCs play critical roles in the progression of at least some human gliomas.

### 2.3 The IGF Signaling Axis Is Important to Sustain OPC‐Like TICs

**Figure 2:**
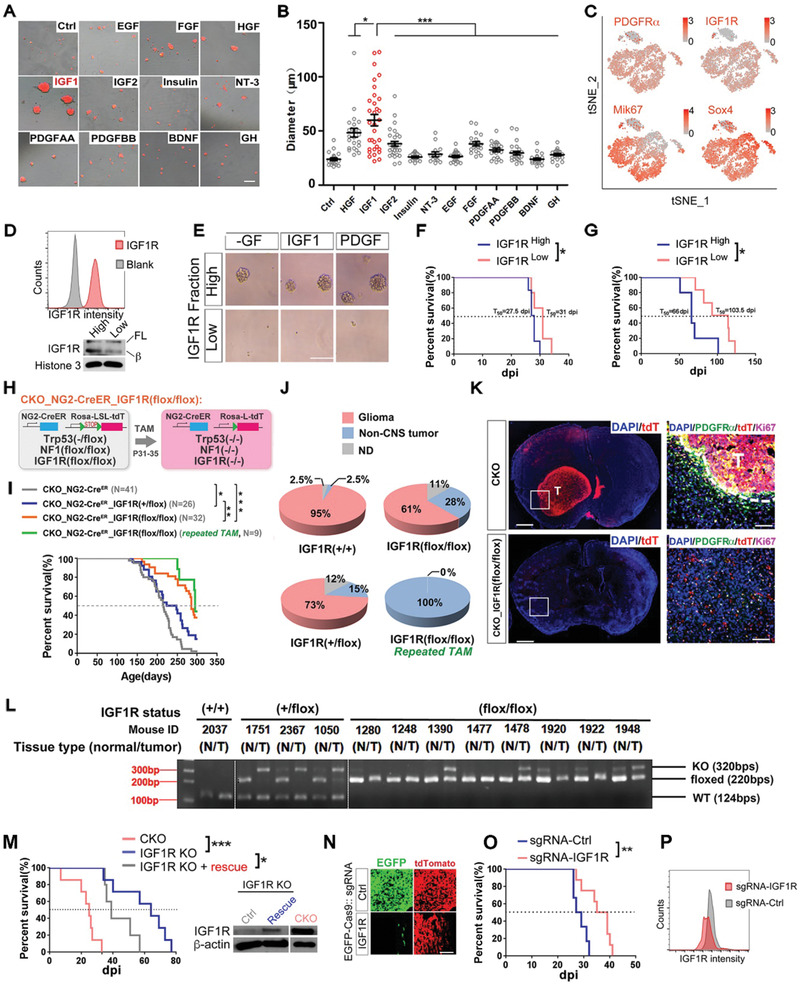
The role of IGF1R in the initiation and progression of the OPC‐derived glioma model. A,B) Representative images (A) and quantification (B) of sphere formation by tumor OPCs in the presence of different growth factors (GFs) as indicated. EGF, epithelial growth factor; FGF, basic fibroblast growth factor; HGF, hepatocyte growth factor; NT‐3, neurotrophin‐3; BDNF, brain‐derived neurotrophic factor; GH, growth hormone. All growth factors were provided as 10 ng mL^−1^. Scale bar: 100 µm. C) The projection of indicated genes on the tSNE map of a tumor OPC cell line that is also shown in Figure 1J. D) The representative FACS plot and Western blots showing the expression of IGF1R on mouse OPC‐like glioma cells. E) In vitro sphere assay of OPC‐like glioma cells based on their surface IGF1R expression sorted by FACS. Scale bar: 100 µm. F,G) The survival curves of mice grafted with IGF1R high/low OPC‐like glioma cells fractions. (F) and (G) are from two independent cell lines, *N* = 6 mice for IGF1R^high^ group and *N* = 5 mice for IGF1R^low^ group in (F), *N* = 5 mice for IGF1R^high^ group and *N* = 6 mice for IGF1R^low^ group in (G). H) The genetic configuration of the CKO_NG2‐Cre^ER^ mouse model with conditional IGF1R knockout. I) Survival curves for the three mouse models as indicated. The median survival times are indicated by the dashed line. For the repeated TAM group, the mouse model was given tamoxifen twice a month following the first round of tamoxifen treatment at postnatal (P) day 31‐P35. For the other three groups, tamoxifen was given only from P31‐P35. J) Tumor incidences in the three mouse models at the endpoint of analysis from (I). The same groups of mice were used for calculating survival curves in (I) and tumor incidence in (J), ND: Not determined. K) Representative images of brain sections from the CKO and CKO‐IGF1R (flox/flox) mice immune‐stained as indicated. The dashed line demarcates the tumor boundary. T, tumor. Scale bars: 1 mm (gross images), 100 µm (zoom‐in section). L) Genotyping data from paired normal and tumor tissues from the three mouse models indicated. N, normal. T, tumor.M) Survival curves of the NOD‐SCID mice orthotopically grafted with the tumor cells as indicated. The IGF1R KO+ rescue cells were the ones from the IGF1R KO tumor OPCs stably transfected with the lentiviral vector that overexpressed IGF1R. The expression/absence of the IGF1R protein from these cells was validated by the western blots, *N* = 7 mice for CKO and IGF1R KO group, *N* = 5 mice for IGF1R KO + rescue group. N–P) Knocking out of IGF1R by the CRISPR‐Cas9 approach. The histological analyses (N) of the tumor sections from these tumors as shown in (O). The survival curves (O) of the NOD‐SCID mice grafted with the indicated tumor cells. FACS analysis (P) confirmed the decreased expression of surface IGF1R protein. Scale bar: 100 µm in (N), *N* = 6 mice for sgRNA‐Ctrl group, *N* = 8 mice for sgRNA‐IGF1R group. Mean ± SEM. :*p* < 0.05, ::*p*<0.01, :::*p* < 0.001.

To identify critical growth factor (GF) receptors for OPC‐originated gliomas, we evaluated the efficiency of a panel of GFs in sustaining the nonadherent growth of mouse tumor OPCs. IGF1 exhibited the strongest ability to sustain nonadherent cultures in GF‐free media (**Figure** **2**A,B). This observation can be repeated by independent cell lines (Figure S4A, Supporting Information). As IGF2 and insulin exhibited much less efficacy (Figure 2A,B), IGF1 likely stimulated OPC‐like TICs through the IGF1R.^[^ ^33^ ^]^ This conclusion is supported by that small molecule IGF1R inhibitors (Figure S4B–G, Supporting Information), CRISPR‐Cas9 (Figure 2N–P), shRNA (Figure S4H,I, Supporting Information), and MicroRNA (Figure S4J–M, Supporting Information)‐based genetic approaches could effectively suppress the growth of tumor OPCs stimulated by IGF1/2. Surprisingly, despite PDGFR*α* was prominently expressed in tumor OPCs and its activation has been believed to be essential for normal OPCs, PDGF alone was not sufficient to support the growth of tumor OPCs (Figure 2A,B; Figure S4A, Supporting Information), suggesting the importance of the proper function of IGF1R for the influx of growth signals mediated by multiple RTKs beyond IGF1R. Indeed, PDGF and FGF2 was unable to rescue the growth of tumor OPCs after IGF1R was knocked down (Figure S4H, Supporting Information). It should be noted that, given that high concentration of insulin can also activate IGF1R, the minimal media used for nonadherent growth assay were devoid of insulin, which was otherwise routinely added for glioma cell culture in many studies.

In order to further delineate the role of IGF1R in tumor OPCs, we reexamined the expression pattern of *IGF1R* in our scRNA‐seq dataset. In the mouse glioma cell line, *IGF1R* highly overlapped not only with the OPC marker *PDGFRα*, but also with those marking proliferation and stemness (Figure 2C). The expression of IGF1R in intact tumor tissues was independently validated by the Western blots and in situ immunofluorescence histochemistry (Figure S4N,O, Supporting Information). These observations lead us to speculate that IGF1R may also function as a TIC marker for gliomas. To validate this speculation, we isolated OPC‐like cells based on their surface level of IGF1R (Figure 2D). Compared to those with minimal IGF1R expression (IGF1R^Low^), the IGF1R^high^ fraction significantly enriched the tumor cells to form spheres in vitro (Figure 2E) and to initiate tumors in vivo (two independent cell lines, Figure 2F,G). Therefore, IGF1 signaling axis likely plays essential roles in maintaining the stemness of tumor OPCs both in vitro and in vivo.

### 2.4 IGF1R Knockout Suppresses the Malignant Transformation of Adult OPCs

To ask whether IGF signaling pathway plays a role during gliomagenesis in the physiological relevant context, we concurrently knocked out *IGF1R* together with *Trp53* and *NF1* in adult OPCs (termed CKO_NG2‐Cre^ER^_IGF1R (flox/flox) model, Figure 2H). Removal of both IGF1R alleles in adult OPCs significantly prolonged the survival of the model (Figure 2I). While less profound, a similar phenotype was observed when one IGF1R allele was removed, suggesting IGF1R has dosage effects on tumor initiation and /or progression. We next examined the tumor incidence in these mice upon the moribund stage. Consistent with the previous report,^[^ ^8^ ^]^ a near‐to‐full penetrance of high‐grade glioma was found (Figure 2J). In rare cases, mice contracted sarcomas outside of the CNS, likely because of the expression of NG2‐Cre^ER^ in non‐CNS cells. In contrast, the glioma incidence significantly decreased in the CKO_NG2‐Cre^ER^_IGF1R (flox/flox) mice, indicating that IGF1R deactivation restrained OPC transformation. This conclusion was further confirmed by immunostaining at 165 days post injection (dpi) of tamoxifen, a time point when most tumor mice showed undetectable symptoms (Figure 2K).

As Cre^ER^‐LoxP system‐mediated gene knockout cannot be 100% efficient, we suspected that the tumors developed in the CKO_NG2‐Cre^ER^_IGF1R (flox/flox) model were due to the incomplete knockout of IGF1R allele in these tumor cells. To confirm this, we genotyped the IGF1R genomic locus. Indeed, despite that a complete deletion of the single IGF1Rflox allele was readily detected in the IGF1R (flox/+) mice (2/3 examined, Figure 2L), the IGF1R flox allele was either intact or with only partial recombination in the IGF1R (flox/flox) tumors (*N* = 17, representatives in Figure 2L). Supporting this notion, we found that repeated administration of tamoxifen (twice a month), which could further improve the recombination activity of NG2‐Cre^ER^, fully blocked glioma incidence in the IGF1R (flox/flox) mice (Figure 2J).We noted that the survival of these mice with repeated tamoxifen administration was only minimally further extended (Figure 2I). This can be explained due to the death caused by other tumors out of the CNS through spontaneous loss of *Trp53*. Supporting this notion, we found that when using the constitutively activated NG2‐Cre transgene in a different model, the death of tumor mice was almost completely prevented (see below in Figure 4). We therefore conclude that the proper function of IGF1R is critical for OPC transformation.

To further address whether IGF1R plays a role in the progression of tumor OPCs in vivo, we utilized the cell line from the CKO_NG2‐Cre^ER^_IGF1R (flox/flox) model where IGF1R was largely knocked out and examined its growth after grafted into the brains of NOD‐SCID mice. Our data showed that the tumor initiating capacity of this line was largely impaired. However, reintroduction of exogeneous IGF1R could partially restore the tumor initiating capacity of this cell line (Figure 2M). In parallel, we performed in vivo competition assay by using the CRISPR‐Cas9 approach (Figure 2N–P). Compared to tumor OPCs transfected with nonspecific sgRNA, those with sgRNAs specific to IGF1R were largely depleted from the tumor (Figure 2N). Correspondingly, the death of tumor mice was partially delayed (Figure 2O). The partial knockout of IGF1R was confirmed by the FACS assay at the protein level (Figure 2P). Taken together, these observations demonstrate that deletion of IGF1R severely compromises the progression, and likely also the initiation, of OPC‐originated glioma.

### 2.5 Knockout of IGF1R Impairs the Growth and Promotes the Differentiation of Mutant Adult OPCs But Minimally Affects Normal OPCs at the Pretransforming Stage

**Figure 3:**
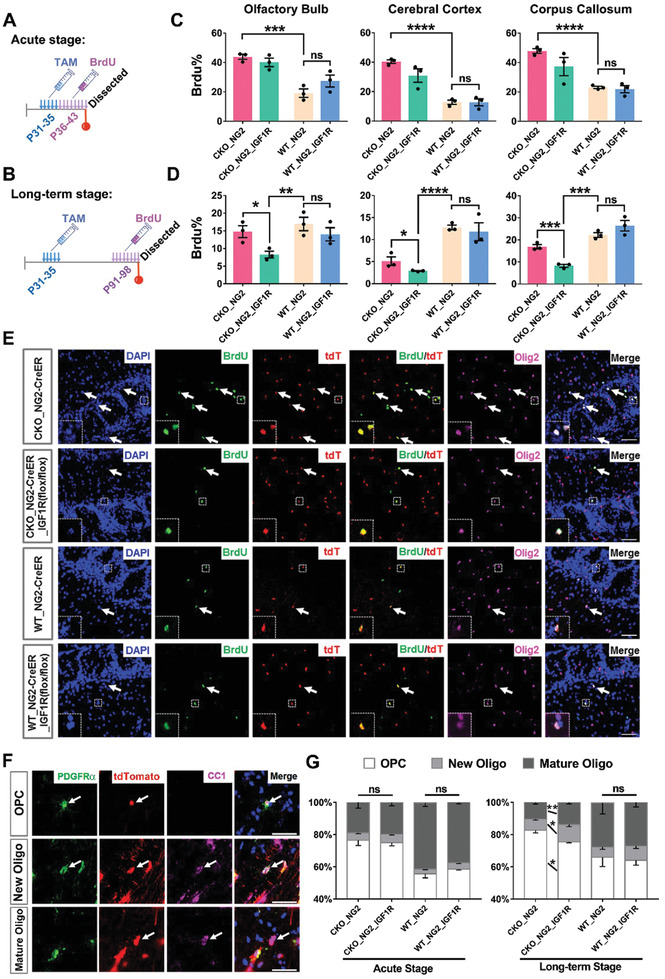
The impacts of IGF1R knockout on the growth of mutant and normal OPCs at the pretransforming stage of tumor development. A,B) Schematics showing the tamoxifen (TAM) and BrdU treatment schedules for the mice analyzed either acutely (A) or long‐term (B). C,D) The quantification of BrdU^+^ cells among all the tdTomato labeled Olig2^+^ cells in the brain regions of mice with the four different genotypes shown, after either the C) acute or D) long‐term experiments. Please refer to Table S7, Supporting Information, for all raw quantification data, *N* = 3 mice for each group. E) Representative images of the brain sections from the olfactory bulb region after the long‐term experiment. The arrows indicate cells coexpressing markers. The insets show a zoom‐in image of the indicated cells. Scale bar: 50 µm. F) Images showing the distinct cell types along the OPC lineage, where OPCs were defined as PDGFR*α* ^+^ and CC1^−^, newly formed oligodendrocytes as PDGFR*α* ^+^ and CC1^+^, and mature oligodendrocytes as PDGFR*α* ^−^ and CC1^+^. Scale bar: 30 µm. G) The relative abundance of the indicated subpopulations from the corpus callosum after the acute and long‐term experiments. *N* = 3 mice for each group. Mean ± SEM. *t*‐test, :*p* < 0.05, ::*p* < 0.01, :::*p* < 0.001, ::::*p* < 0.0001, ns, no significance.

We next set out to determine whether IGF1R deactivation suppressed the transformation of mutant OPCs at the pretransforming stage through compromising their capacity to proliferate and differentiate into mature oligodendrocytes. The proliferation rate of mutant OPCs at two time points, corresponding to the acute and long‐term stages during OPC tumorigenesis (**Figure** **3**A,B),^[^ ^8^ ^]^ was determined by BrdU incorporation. Consistent with the previous report,^[^ ^8^ ^]^ we found that mutant OPCs initially experienced a transient elevation of their proliferation rate after acquiring mutation (compared the first vs the third bars in Figure 3C), and then the proliferation of these mutant cells declined to the level of their normal control (compare the first vs the third bars in Figure 3D, and the first bars between Figure 3C,D). IGF1R knockout decreased the proliferation of mutant OPCs at both stages in various brain regions, with the long‐term stage becoming more prominent (the first vs the second bars in Figure 3C,D). In combination of two cellular markers, PDGFR*α* and CC1, to delineate subpopulations throughout the OPC lineage (Figure 3F), we further revealed that IGF1R knockout promoted the differentiation of mutant OPCs at the pretransforming stage (Figure 3G).

In contrast, we found that knockout of IGF1R minimally affect the proliferation (compared the third and the fourth bars in Figure 3C,D, representative images in Figure 3E) or differentiation potential (Figure 3G) of wild‐type OPCs within the same observation window. In fact, WT‐NG2‐Cre^ER^_IGF1R (flox/flox) mice manifested no detectable abnormality up to 400 dpi (data not shown), further supporting that IGF1R is not essential for the physiology of normal adult OPCs. Therefore, it appears that the impacts of IGF1R knockout on mutant OPCs were specifically associated with the oncogenic state but not the intrinsic identity of their normal cell‐of‐origin.

### 2.6 MADM Reveals That IGF1R Knockout Preferentially Affects Mutant OPCs in a Mosaic Context

**Figure 4:**
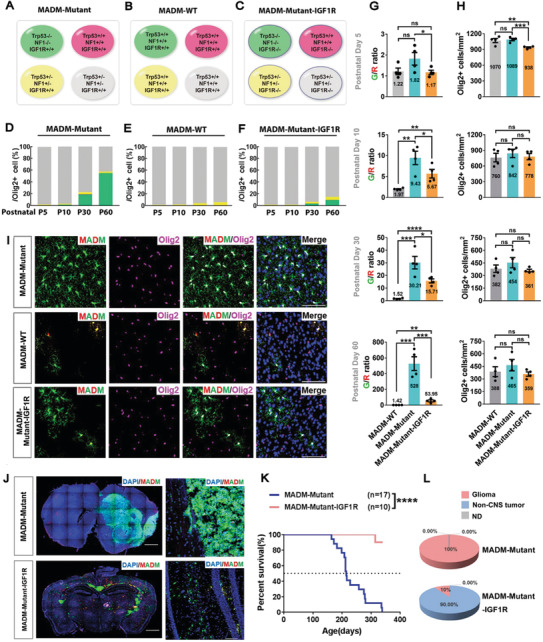
IGF1R knockout in a MADM glioma model. A–C) Genetic configurations of the OPC‐lineage cells in the MADM‐based models. Notably, in (C) all OPC‐lineage cells, not only those labeled by MADM, were IGF1R (‐/‐). MADM‐labeling was induced by the NG2‐Cre. The color for each genotype indicates the expression of the fluorescent protein(s) in the cell. Red, tdTomato; green, EGFP; yellow, both tdTomato and EGFP; gray, no fluorescent protein expressed (nonlabeled). D–F) The abundance of each genotype of Olig2^+^ cells among all the OPC‐lineage cells in the D) MADM‐Mutant, E) MADM‐WT, and F) MADM‐Mutant‐IGF1R models at the four developmental time points as indicated. The color codes of the cells in (D), (E), and (F) are the same as those in (A), (B), and (C), respectively. The data for each group were the average from *N* = 4 mice. G) The G/R ratios of MADM‐labeled Olig2^+^ cells from the brains of the three models analyzed at the four development time points indicated, *N* = 4 mice for each group. H) The density of all OPC‐lineage cells from the mice in (G), *N* = 4 mice for each group. I) Representative images of the cortical brain sections from (A–C) at P60. Olig2 was used to mark all OPC‐lineage cells. Scale bar: 50 µm. J) The representative images of the brain sections from the indicated models at the end‐point stage. Scale bars: 1 mm (gross images), 100 µm (zoom‐in section). K,L) The survival curves (K) and the tumor incidences (L) of the models as indicated. Of note, the IGF1R allele of the only tumor mice from the MADM‐Mutant‐IGF1R model was intact based on the IGF1R genotyping (not shown), ND: Not Determined. Please refer to Table S7, Supporting Information, for all raw quantification data in Supporting Information. Mean ± SEM. :*p* < 0.05, ::*p* < 0.01, :::*p* < 0.001, ::::*p* < 0.0001, ns, no significance.

In patients, only a small number of normal cells acquired initial mutations. Therefore, we asked whether, in an in vivo context where mutant OPCs are generated sparsely, knockout of *IGF1R* preferentially suppresses the overproliferation of mutant OPCs compared to their surrounding normal siblings. To address this question, we leveraged a genetic mouse model termed Mosaic Analysis with Double Markers (MADM),^[^ ^7^, ^34^ ^]^ which we have previously used to mimic sporadic somatic mutagenesis in glioma patients (see also working scheme in Figure S5A, Supporting Information).^[^ ^7^ ^]^ In the MADM‐Mutant model (**Figure** **4**A), mitotic recombination mediated by the NG2‐Cre transgene enables pairs of GFP‐labeled mutant and tdTomato‐labeled wild‐type OPCs to be generated simultaneously from unlabeled heterozygous OPCs. As a control, we generated a MADM‐WT model in which both green and red OPCs possessed the wild‐type genotype (Figure 4B). To knock out of *IGF1R* among all OPCs with distinct genotypes, we introduced the homozygous IGF1R (flox/flox) alleles into MADM‐Mutant to generate the MADM‐Mutant‐IGF1R model (Figure 4C).

Consistent with our previous observation,^[^ ^7^ ^]^ the abundance of mutant OPC‐lineage cells in the MADM‐Mutant model progressively increased from 0.373% at P5 up to 55.12% at P60 (Figure 4D,I; Figure S5B, Supporting Information). Accordingly, the average G/R ratio (the relative number of green Mutant to red WT cells) drastically increased from 1.82 at P5 to 528.00 at P60 (Figure 4G). As the G/R ratio reflects the severity of the overexpansion of mutant OPCs compared to their normal siblings, these results demonstrate that mutant OPCs possessed a much more elevated proliferation rate compared to their surrounding WT siblings. In contrast, in the MADM‐WT model, the relative abundance of green WT cell always remained below 0.5% of all OPC‐lineage cells (Figure 4E,I) and the average G/R ratio was close to one (Figure 4G). Notably, the total number of OPC‐lineage cells was not significantly different between the two models at all four developmental time points examined (Figure 4H), suggesting that mutant OPCs increased their number at the expense of normal OPCs, possibly through cell‐to‐cell competition.

In stark contrast, we found that the overexpansion of mutant OPCs was significantly suppressed after IGF1R was knocked out (Figure 4F,I). Knockout of IGF1R also significantly reduced the G/R ratio, particularly at the later time point such as P60 (Figure 4G). This latter observation further supports that mutant OPCs are more sensitive than their WT siblings to IGF1R deprivation (otherwise, the G/R ratio should not be affected by the IGF1R status). Together with the findings that no significant change in the total number of OPC‐lineage cells was detected among the three MADM models at all developmental time points except for P5 (Figure 4H), we propose that IGF1R deactivation affects mutant and adjacent WT OPCs in opposite ways: it suppresses the overexpansion of mutant OPCs but recovers the number of normal OPCs, like by rebalancing the growth fitness between the two genotypes of OPCs. Furthermore, we also found that, although MADM‐Mutant model fully developed glioma around 8 months, the MADM‐Mutant‐IGF1R model exhibited no obvious symptoms up to 400 days (Figure 4J–L). These observations, together with those in Figure 2 by using a distinct model system, further support that IGF1R is critical for OPC transformation in vivo.

### 2.7 Knockout of IGF1R Minimally Affects Adult Mutant NSCs until They Commit to the OPC Identity

Does the identity of the cells among the development hierarchy into which the driver mutations were initially introduced (we named as the cell‐of‐mutation to distinguish the cell‐of‐origin, although both concepts are not necessarily mutually exclusive)^[^ ^7^ ^]^ also determine the sensitivity of IGF1R targeting? It is well known that NSCs can give rise to OPCs throughout life.^[^ ^35^ ^]^ Accordingly, knocking out of *Trp53* and *NF1* directly in NSCs, as in OPCs, can efficiently generate high‐grade gliomas.^[^ ^4^, ^7^ ^]^ Given that adult NSCs and OPCs are in the same development hierarchy during gliomagenesis but possess distinct cell identities, a genetic model using adult NSCs as the glioma cell‐of‐mutation will help to address this question.

**Figure 5:**
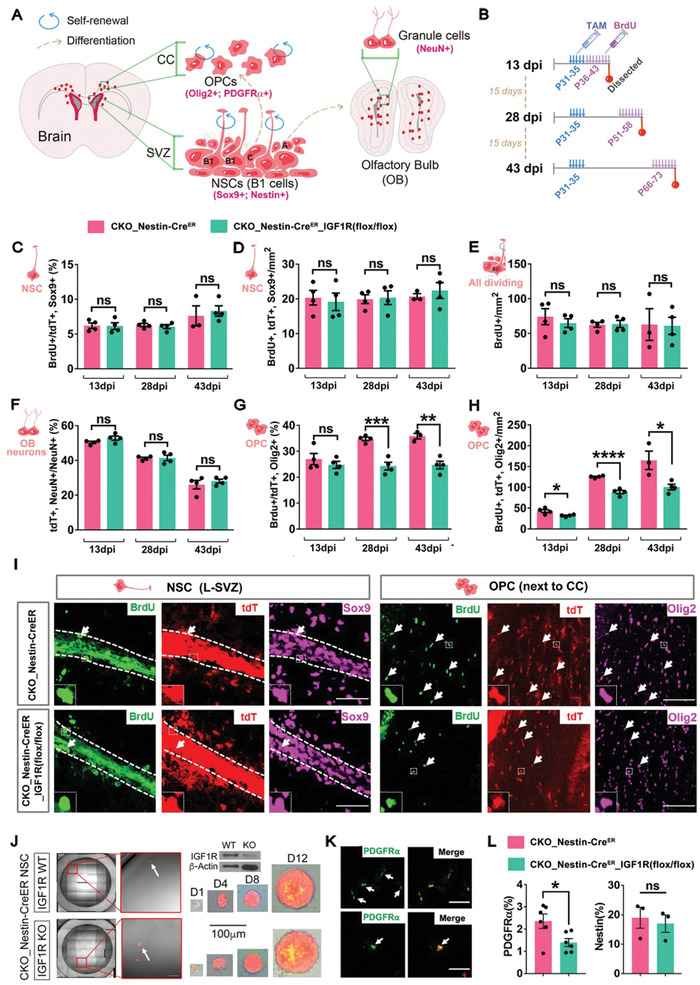
The susceptibility of NSCs and their progeny toward IGF1R targeting. A) Schematic showing the differentiation pattern of adult NSCs after acquiring the initial mutations in the mouse model. B) The schedules of tamoxifen (TAM) and BrdU administration for the three time points analyzed. C) The percentage of BrdU^+^ cells among all tdTomato^+^ and Sox9^+^ mutant cells in the SVZ from the two mouse models at three different time points as indicated. D,E) The density of BrdU^+^, tdTomato^+^ and Sox9^+^ proliferating mutant NSCs (D) and the density of all BrdU^+^ proliferating cells (E) in the SVZ from the two mouse models at three different time points as indicated. F) The percentage of mutant granule neurons among all neurons in the OB from the two models indicated. G,H) The percentage and density of proliferating mutant OPCs in the CC next to the SVZ (region 6 in Figure S7C, Supporting Information). L‐SVZ, the lateral subventricular zone; CC, the corpus callosum. I) Representative images of the brain sections from the two models at 43 dpi. Scale bar: 50 µm for the NSC panels, 100 µm for the OPC panels. J) Ex vivo culture of mouse adult NSCs with the genotypes as indicated. Scale bar: 200 µm for the left panel, 100 μm for the right panel. K,L) Representative images (K) and the quantification (L) of in vitro differentiation of adult NSCs as indicated. 2% serum was used to induce the differentiation of NSCs. Scale bar: 100 µm in (K). Mean ± SEM.:*p* < 0.05, ::*p* < 0.01, :::*p* < 0.001, ::::*p* < 0.0001, ns, no significance. *N* = 4 mice for all groups except for the CKO‐Nestin‐Cre^ER^ group at 43 dpi in (C)–(E), (G), and (H), where *N* = 3. Please refer to Table S7, Supporting Information, for all raw quantification data in Supporting Information.

We therefore generated a pair of mouse models with knockouts of *Trp53* and *NF1* with/without IGF1R ablation in adult NSCs using a Nestin‐Cre^ER^ transgene. The specificity of this transgene to label adult NSCs, but not *directly to* OPCs, was validated in Figure S6, Supporting Information. We detected a clear accumulation of mutant OPCs in the brain of the CKO_Nestin‐Cre^ER^ model after tamoxifen treatment (from 13 to 43 dpi, three time points examined), particularly at the locations next to the SVZ, which was otherwise rarely seen in the WT control brain (Figure S7, Supporting Information). These observations, together with the previous findings by us and others,^[^ ^7^, ^36^, ^37^ ^]^ support the notion that, upon acquiring mutations, adult NSCs preferentially give rise to OPCs, which then expand, similar to the situation where the same mutations were directly introduced into OPCs (as illustrated in **Figure** **5**A).

As in adult normal NSCs and normal OPCs,^[^ ^38^ ^]^ but not mutant OPCs, at all three time points examined (Figure 5B), *IGF1R* knockout in mutant adult NSCs did not significantly affect the proliferation rate or the number of these cells (Figure 5C,D,I). Even with the total number of BrdU^+^ cells as criteria, which included adult NSCs and their fast dividing progenitors, no alterations were detected (Figure 5E).The number of mutant granule cells in the olfactory bulb was not affected either (Figure 5F), further supporting that IGF1R deactivation minimally affects the self‐renewal of mutant adult NSCs in vivo. In contrast, when we examined mutant OPCs in these brains, we found a significant decrease in their proliferation when compared with their IGF1R‐intact counterparts in the CKO_Nestin‐Cre^ER^ brains (Figure 5G–I). This difference has already become prominent at 13 dpi, the earliest time point that we could detect the emergence of significant numbers of mutant OPCs. These data clearly demonstrate that adult pretransforming cells manifesting the cell identity of OPCs, but not NSCs, respond to the loss of IGF1R, at least read by their proliferation rates.

To further confirm this conclusion, we isolated adult NSCs from both models and assayed their self‐renewal and differentiation potential ex vivo (Figure 5J). Consistent with the in vivo data, we found that knockout of IGF1R did not affect the size of neurospheres (Figure 5J). However, the potential of NSC differentiation toward OPC lineage was significantly suppressed in IGF1R‐KO mutant NSCs compared to their IGF1R intact counterparts (Figure 5K,L). The distinct susceptibility of NSCs and OPCs to IGF1R knockout cannot be explained as the differences of their IGF1R expression, as both types of cells, regardless of aquiring mutations, expressed similar level of IGF1R as detected by qRT‐PCR (data not shown). Together, we propose that, in addition to the oncogenic state, the identity of the cell upon acquiring driver mutations is also crucial in the susceptibility to IGF1R targeting.

### 2.8 The IGF1R Is Preferentially Activated in Proliferating Tumor OPCs from Human Gliomas

**Figure 6:**
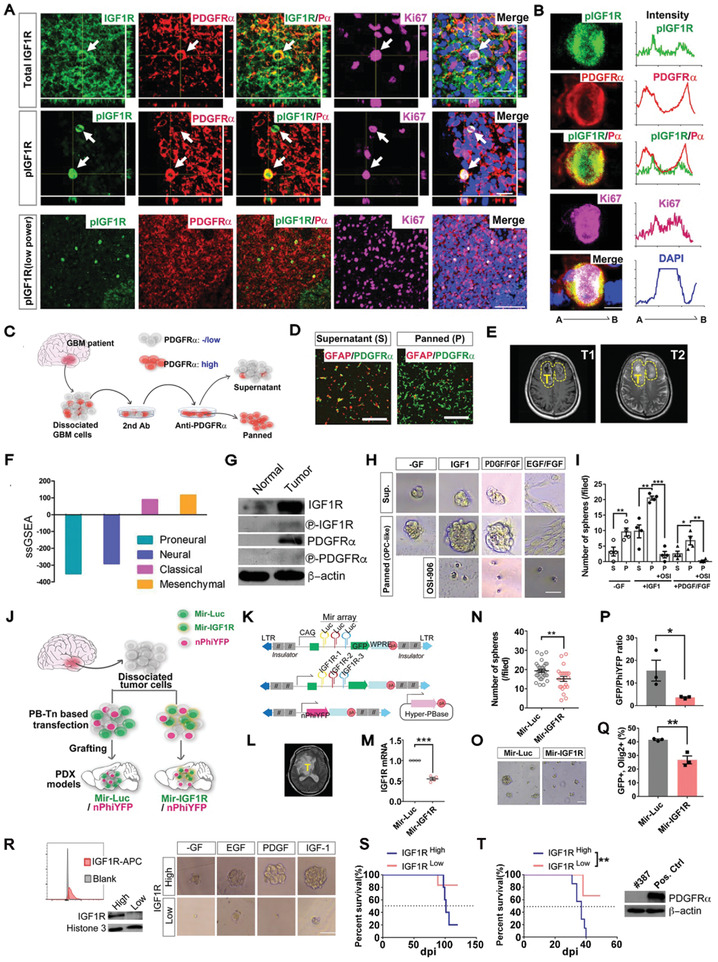
The susceptibility of human tumor OPCs toward IGF1R targeting. A) Representative images showing that IGF1R and pIGF1R are expressed in proliferating tumor OPCs from a GBM sample. The arrows indicate the cells coexpressing markers indicated. The low power images are provided at the bottom row to show no bias of imaging collection. *Z*‐axis orthogonal views are provided in zoom‐in images to confirm the colocalization of the markers. Scale bars, 20 µm in the top and middle rows; 100 µm in the bottom row. B) Zoom‐in of images to show the expression of the indicated markers in one tumor OPC. The fluorescence intensity across the middle plane of the cell is shown. Scale bars, 3 µm. C) Schematic diagram showing the immunopanning method used to enrich human tumor OPCs. D) Representative images showing that PDGFR*α*‐positive cells were enriched in the panned fraction. Conversely, GFAP‐positive tumor cells were largely depleted but appeared in the supernatant fraction. Scale bar: 400 µm. E–G) The MRI images (E), subtype analysis (F), and Western blots (G) of indicated proteins for a human GBM (#H5). Additional histological and pathological information of this tumor case can be found in Figures S3 and S8, Supporting Information. H,I) Representative images and the quantification from a sphere assay of a primary human GBM cell line (#H63) treated with the indicated growth factors. S: supernatant fraction, P: immunopanned tumor OPCs fraction. IGF1, 10 ng mL^−1^; EGF, 50 ng mL^−1^; FGF, 20 ng mL^−1^; PDGF, 20 ng mL^−1^. OSI‐906, 0.5 µm. Scale bar: 25 µm in (H). J) Schematic diagram showing the strategy to genetically knock down IGF1R in patient‐derived xenograft (PDX) models. K) Piggybac (PB) transposon vectors encoding multiplex miRNAs against different sites of IGF1R (Mir‐IGF1R) or against Luciferase (Mir‐Luc). L) The MRI image of the fresh tumor sample used in (P,Q). M) The knock down of IGF1R was validated by qPCR. N,O) The quantification (N) and the image (O) of tumor spheres after IGF1R was knocked down by MirRNA. Scale bar: 100 µm in (O). P,Q) The quantification results from the PDX model shown in (L), *N* = 3 mice for each group. R,S) The sphere assay (R) and the survival curves (S) of NOD‐SCID mice grafted with human OPC‐like glioma cells (#H5) based on their surface IGF1R expression. Scale bar: 100 µm in (R), *N* = 5 mice for IGF1R^high^ group and *N* = 6 mice for IGF1R^low^ group in (S). T) The survival curve of NOD‐SCID mice grafted with a human glioma stem cell line based on their surface IGF1R expression. Of note, this cell line does not express PDGFR*α*, *N* = 7 mice for IGF1R^high^ group and *N* = 6 mice for IGF1R^low^ group. Mean ± SEM. :*p* < 0.05, ::*p* < 0.01, :::*p* < 0.001.

To directly determine whether the IGF1R is activated in human tumor OPCs, we analyzed the tumor tissue from a GBM patient with the classical subtype (Figure S3A, Supporting Information). Similar to our mouse model studies shown in Figure S4N, Supporting Information, both total IGF1R and pIGF1R were found to be present in these human tumor OPCs (**Figure** **6**A). pIGF1R was most frequently detected in tumor cells expressing both Ki67 and PDGFR*α*. The prominent cytoplasmic localization of pIGF1R (Figure 6A,B; Figure S8A, Supporting Information), as shown in other type of tumors previously reported,^[^ ^39^ ^]^ further supports the activation of IGF1R in these cells. Similar results were observed in multiple GBMs and lower‐grade human gliomas (*N* = 5 for each grade, Figure S8B–D, Supporting Information). Of note, while pIGF1R^+^ cells represented only a small fraction of total cells, most were tumor OPCs. Moreover, the percentage of proliferating tumor OPCs among all pIGF1R^+^ cells was positively associated with the tumor grade (Figure S8D, Supporting Information). Therefore, we conclude that in human gliomas, particularly GBMs, IGF1R‐mediated signaling can be activated in tumor OPCs that are undergoing proliferation.

### 2.9 IGF1R Is Important for the Growth of Human Tumor OPCs Both In Vitro and In Vivo

We next determined whether human tumor OPCs require IGF1R and are sensitive to IGF1R inactivation. As long‐term culture of GBM cells in either serum or stem cell media with EGF may lead to the loss of tumor OPCs,^[^ ^25^ ^]^ we reestablished primary GBM cell lines de novo or directly used those freshly isolated from surgical samples to study tumor OPCs from patients. We first established a simple approach to enrich tumor OPCs based on the anti‐PDGFR*α* immunopanning technique (Figure 6C). Immunostaining (Figure 6D), RNA‐seq, qPCR analyses and ssGSEA (Figure S9A–C, Supporting Information) at the transcriptomic level confirmed successful enrichment of human tumor OPCs by this approach. Interestingly, the gene ontology (GO)‐term analysis suggested that the genes involved in both IGF and PDGF signaling pathways were consistently enriched in tumor OPC fraction (*N* = 4 GBMs, Figure S9D, Supporting Information), highlighting the intimate interaction between these two pathways in human tumor OPCs.

We next selected a tumor sample that contained numerous tumor OPCs (Figure 6E; Figure S3A, Supporting Information) despite molecular classification confirmed its mesenchymal subtype GBM (Figure 6F). Both WB (Figure 6G) and immunofluorescence staining (Figure S3A, Supporting Information) confirmed that activation of IGF1R in this tumor, mainly in tumor OPCs. Similar to that in the mouse model, immunopanning enriched tumor OPCs were the main fraction to grow in insulin‐free minimal media regardless the growth factor provided (Figure 6H,I). Interestingly, EGF/FGF did not support the nonadherent growth of either fractions from this line (Figure 6I).

We further conformed that the observation could be repeated by multiple GBM samples harboring a diverse variety of genetic aberrations and were stratified into distinct subtypes, in either nonadherent or monolayer cultures (Figure S10, Supporting Information). Akin to their mouse counterparts, human tumor OPCs exhibited a strong preference for IGF1. Importantly, IGF1R inhibitor OSI‐906 not only suppressed sphere formation induced by IGF1, but also that by other growth factors (Figure S10, Supporting Information). Similar results were observed by using PPP (Figure S10J, Supporting Information) and RNAi‐based genetic approach (Figure 6N,O), excluding the possibility that the observed inhibition of tumor spheres was caused by the off‐target effect of the inhibitors.

To further determine the role of IGF1R in the growth of human OPC tumor cells in vivo, we acutely transfected patient‐derived glioma cells with Piggybac (PB) transposon vectors encoding multiplex miRNAs against different sites of IGF1R (Mir‐IGF1R) or against Luciferase (Mir‐Luc) (Figure 6J,K). The design to include multiple miRNAs targeting distinct sequences in the same transcript could maximally increase the knockdown efficiency. As the tumor cells were derived directly from the patient (Figure 6L) with minimal exposure to in vitro conditions (less than 12 h) and electroporation delivered vectors into all tumor cell types equally, this patient‐derived xenograft (PDX) model offered a unique opportunity to evaluate the effects of IGF1R deprivation on OPC‐like and non‐OPC‐like cells in the same tumor, which helped to address the question whether distinct subpopulation of in the same tumor possessed the same susceptibility to IGF1R targeting. The knockdown of IGF1R was validated by the qPCR assay (Figure 6M). The results show that, not only did the relative abundance of Mir‐IGF1R‐expressing tumor cells (normalized to nPhiYFP^+^ cells serving as the internal reference in the same tumor) decrease (Figure 6P), but the percentage of Olig2^+^ cells among all Mir‐IGF1R‐expressing tumor cells also significantly decreased when compared to the nonspecific control (Figure 6Q). Similar results were also observed in another two sets of PDX models with tumor cells derived from different GBM patients (data not shown). These results indicate that IGF1R targeting suppresses the growth of human GBM cells in vivo and that tumor OPCs are more susceptible to IGF1R targeting. These results collectively substantiate our conclusion that human tumor OPCs among tumor hierarchy are preferentially susceptible to IGF1R targeting, paralleling to tumorigenic process shown in the mouse model studies.

Finally, we tested whether IGF1R could function as the TIC markers for human glioma cells. We found that IGF1R‐based sorting enriched the human GBM cells to form spheres in vitro (Figure 6R) and initiate tumors in vivo (Figure 6S). Therefore, we conclude that IGF1R is critical for the stemness of human OPC‐like tumor cells both in vitro and in vivo, nicely recapitulating our mouse work in Figure 2. Notably, IGF1R may be considered as a TIC marker independent of the OPC feature in some cases. The TICs in a classical human GBM stem cell line T387,^[^ ^3^ ^]^ which was completely absent of PDGFR*α*, can also be enriched based on their IGF1R expression (Figure 6T).

### 2.10 IGF1R Is a Key Player That Controls the Influx of Growth Signals into Tumor OPCs

**Figure 7:**
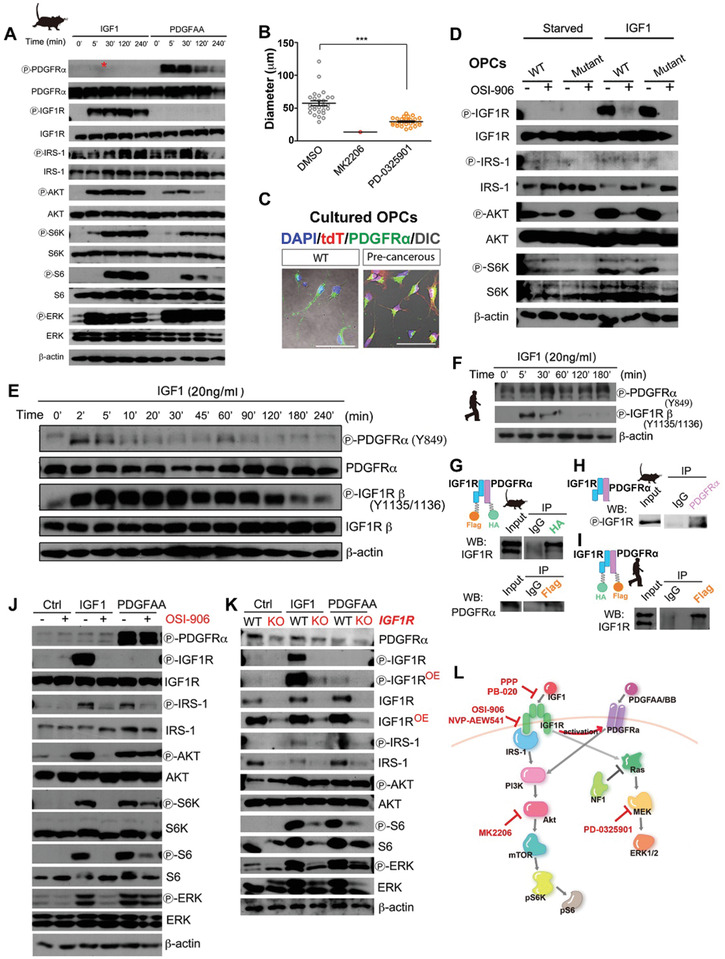
The crosstalk of IGF1R and PDGFR*α* pathways in tumor OPCs. A) Activation of signaling pathways in tumor OPCs after stimulation with IGF1 (20 ng mL^−1^) or PDGFAA (20 ng mL^−1^) for the indicated periods. OE, overexposed. The red asterisk indicates the presence of pIGF1R after PDGFAA stimulation for 120 min. B) Sphere assay for mouse tumor OPCs treated with the indicated inhibitors. Spheres were formed in the presence of 10 ng mL^−1^ IGF1. Mean ± SEM. :::*p* < 0.001. C) Representative images showing purified mutant and WT OPCs used for the biochemical analysis in (D). Scale bar: 50 µm. D) The response of WT and *Trp53/NF1* double mutant OPCs to IGF1 in the presence or absence of OSI‐906. E) Activation of indicated RTKs in mouse tumor OPCs after stimulation with IGF1 for the indicated periods. F) Activation of indicated RTKs in human tumor OPCs after stimulation with IGF1 for the indicated periods. G,H) Co‐IP assay to determine the interaction between mouse PDGFR*α* and IGF1R. Exogeneous expression of mouse PDGFR*α* and IGF1R in either HEK293 cells (G) or the endogenous PDGFR*α* and IGF1R in mouse tumor OPCs (H) was performed separately. I) Co‐IP assay to determine the interaction between exogenously expressed human PDGFR*α* and IGF1R in HEK293 cells. J) The effect of OSI‐906 on IGF1 (20 ng mL^−1^) and/or PDGFAA (20 ng mL^−1^) signal transduction. The cells were analyzed 2 h after OSI‐906 (0.5 µm) treatment. K) IGF1R‐null mouse tumor OPCs responded to the stimulation of growth factors (20 ng mL^−1^) as indicated. OE, overexposed. L) Schematic showing the hypothetical crosstalk between IGF1R and PDGFR*α* in tumor OPCs.

We next investigated the molecular mechanisms by which IGF1R preferentially regulates the growth of tumor OPCs and why other GFs such as PDGF cannot effectively rescue the growth of tumor OPCs without the proper function of IGF1R. We first examined the dynamic response of tumor OPCs to IGF1 or PDGFAA stimulation. As shown in **Figure** **7**A, compared to PDGFAA, IGF1 elicited a much stronger and more durable activation of the PI3K‐Akt signaling, indicating that IGF1 is more potent than PDGFAA to sustain the PI3K‐Akt pathway in these cells. Instead, PDGFAA was more effective in activating ERK. Same results could be observed by using mutant OPCs at the pretransforming stage (data not shown). Consistent with previous report by using different GBM cell lines,^[^ ^15^ ^]^ we found that tumor OPCs employed more on PI3K‐Akt signaling than the Ras‐Raf‐MEK‐ERK signaling cascade to support their growth (Figure 7B; Figure S11A,B, Supporting Information). This suggests that IGF1R may regulate OPCs mainly via the downstream PI3K‐Akt pathways throughout the tumor development stages.

Intriguingly, in our lineage tracing experiment at the pretransforming stage, we have observed that the proliferation rate of IGF1R‐deficient mutant OPCs was even lower than that of wild‐type OPCs at the long‐term stage (compare the second vs the third bars in Figure 3D). This implicated that mutant OPCs relied more on IGF1R signaling to sustain their growth. If the PI3K‐Akt axis is involved in this process, we would expect that inactivation of IGF1R should induced more profound effect in mutant OPCs than the wild‐type ones on the components of PI3K‐Akt axis. To prove this hypothesis, we enriched the WT and *Trp53/NF1*‐null mutant OPCs from neonatal mouse brains (Figure 7C) and indeed showed that in culture IGF1R inhibitor OSI‐906 only partially inhibited the PI3K‐Akt cascade in WT OPCs but completely blocked its activity in mutant OPCs (Figure 7D). Interestingly, the same phenotype can also be observed in WT and *Trp53/NF1*‐null MEFs (Figure S11I, Supporting Information). Therefore, this observation, together with the in vivo studies (Figures 3 and 4), strongly suggest that the oncogenic state can reprogram the signaling network, and drive the mutant OPCs to depend more, if not solely, on IGF1R‐mediated influx of extracellular signals to sustain the PI3K‐Akt signaling.

It has been puzzled that tumor OPCs do not need PDGF for their growth in vivo, although wild‐type OPCs absolutely require it. Unexpectedly, we found that, in addition to efficiently activate IGF1R, IGF1 also induced the phosphorylation of PDGFR*α* in mouse tumor OPCs (Figure 7E). This observation could be also observed in human tumor OPCs (Figure 7F). Therefore, our finding leads to the novel proposition that tumor OPCs may use IGF1R to activate both pathways for their maximal fitting in the in vivo condition. As the activation of PDGFR*α* occurred within 2 min after IGF1 treatment (Figure 7E,F), the transactivation of PDGFR*α* was likely through the protein‐protein interaction at the post‐translational level. Coimmunoprecipitation (Co‐IP) assay confirmed that PDGFR*α* and IGF1R from mouse tumor OPCs could form complex (through both ectopic expression and directly testing the endogenous proteins), either through direct or indirect interaction (Figure 7G,H). Similar results could be obtained in human context (Figure 7I).

On the other way around, our pharmacological and RNAi experiments demonstrated that even saturated amount of PDGFAA could not support tumor OPCs to growth without the proper function of IGF1R (Figure 6H; Figures S4H and S10, Supporting Information). At the molecular level, we found that IGF1R inhibitor OSI‐906 not only suppressed the activation of the PI3K‐Akt cascade stimulated by IGF1, but also that by PDGFAA (Figure 7J). A similar result was obtained using a different IGF1R inhibitor, genetic knockout of endogenous IGF1R, in multiple mouse and human primary glioma cell lines with different genetic mutations (Figure S11C–G, Supporting Information). Interestingly, OSI‐906 did not affect PDGFAA‐induced PI3K activation in human HEK293 cells (Figure S11H, Supporting Information), suggesting that this phenomenon depends on cellular contexts. Of note, we found that in IGF1R‐knockout tumor OPCs, PDGFR*α* protein lever was significantly decreased (Figure 7K), raising the interesting possibility that the interaction between IGF1R and PDGFR*α* may help to stabilize PDGFR*α* proteins and facilitate the PDGFR*α*‐mediated signaling, and therefore inactivation of IGF1R by degradation may have more profound effects than merely blocking its kinase activity. Taken together, these results support that IGF1R is a key player that controls the influx of growth signals into tumor OPCs by two folds of mechanism: 1) in the context without PDGF, the activation of IGF1R can activate PDGFR*α* at the same time; and 2) in the context with PDGF, the action of PDGFR*α* requires the existence of IGF1R (as proposed in Figure 7L).

### 2.11 A Novel Blood–Brain Barrier Penetrable IGF1R Inhibitor Efficaciously Suppresses the Propagation of GBM Cells Grafted into the Mouse Brain

**Figure 8:**
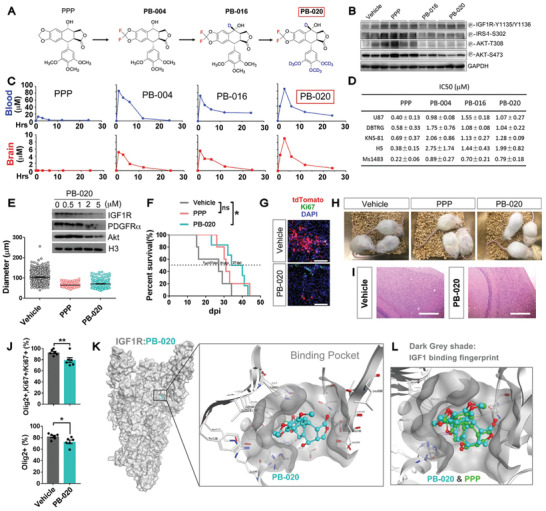
A new generation of oral IGF1R inhibitor that can penetrate the blood–brain barrier. A) Chemical evolution of PB‐020. B) The Western blots of the major pathways in the brain tissues from the mice that orally treated with the vehicle or the inhibitors as indicated. C) HPLC analyses of the brain extracts from the mice orally treated with the compound as indicated. Mice were dosed by gavage with 50 mg kg^−1^. At predosing (0 h) and different post‐dosing time points, the mice were euthanized, and their blood and brain samples were processed for HPLC analysis. D) IC_50_ of five lines of adherent glioma cell lines as indicated (*N* = 3, mean ± SD). Among them, U87MG, DBTRG, and KNS‐81 lines were maintained in 10% serum. Mouse glioma cell line Ms1483 and human GBM cell line #H5 were maintained in the stem cell media. E) The sphere assay of mouse tumor OPCs treated with the indicated inhibitors (0.5 µm) and 20 ng mL^−1^ IGF1. Western blots showing that PB‐020 also decreased the total level of both IGF1R and PDGFR*α*. F) Survival curves of NOD‐SCID mice orthotopically grafted with mouse tumor OPCs and orally treated with vehicle, PPP or PB‐020. *N* = 5 mice for vehicle and PPP group, *N* = 6 for PB‐020 group. G) Representative images of the brain sections from (F). Scale bar: 100 µm. H,I) Photos of mice and the H&E staining of the brain sections (including the hippocampus) treated with the vehicles or inhibitors as indicated. Scale bar: 400 µm in (I). J) The percentage tumor OPCs (Olig2^+^) among proliferating (top) or all tumor cells (bottom) in the tumor mice after treated with vehicle or PB‐020. Mean ± SEM, *N* = 6 for each group. K) The docking model of PB‐020 in the ligand‐binding site 1 of apo IGF1R. The zoom‐in view of PB‐020 bound in the binding site 1 of IGF1R. L) The comparison of the docked binding modes of PPP and PB‐020 in the ligand‐binding site 1 of apo IGF1R. Comparing to PPP, PB‐020 is pushed out a little bit further to the entrance of the IGF1 binding pocket (marked as dark gray shade) because of two fluorine atoms. All oxygen atoms are colored in red. :*p* < 0.05, ::*p* < 0.01, ns, no significance.

To pave the way for translating these findings into developing an IGF1R‐targeted GBM therapy, we pursued lead optimization for the well‐studied IGF1R inhibitor picropodophyllin (PPP, or AXL1717) that has been tested in clinical trials with tolerable systemic toxicity but exhibits poor penetration across the mouse blood–brain barrier (BBB; as shown in **Figure** **8**B,C).^[^ ^23^, ^40^, ^41^ ^]^ We designed and tested a series of novel PPP analogs (Figure 8A), and identified compound PB‐020 as a promising candidate for further development based on multiple criteria including BBB penetration capability assessed by biomarkers (Figure 8B) or directly measured by HPLC (Figure 8C), in vitro drug efficacy in cultured adherent glioma cells (Figure 8D) and tumor spheres (Figure 8E). Remarkably, oral administration of PB‐020 not only effectively suppressed the propagation of tumor OPCs that had been orthotopically grafted into mouse brains but also prolonged the survival of tumor mice (Figure 8F,G). Furthermore, long‐term oral administration of PB‐020 showed no obvious toxicity to tumor mice (Figure 8H,I; Figure S12, Supporting Information). PB‐020 inhibited the proliferation of tumor OPCs more than their non‐OPC counterparts (Figure 8J). These observations not only demonstrate the potential value of newly developed BBB‐penetrable oral IGF1R inhibitors in GBM treatment, but also substantiate our above conclusion that tumor OPCs are preferentially susceptible to IGF1R targeting.

To elucidate the interaction mode between PB‐020 and the IGF1R, we performed a molecular docking simulation by positioning PB‐020 into the ligand‐binding pocket of IGF1R apo structure (Figure 8K).^[^ ^42^ ^]^ When docked in the same manner, PB‐020 and PPP took similar binding modes to the pocket. Based on the full length structures of IGF1R in the presence or absence of its ligand IGF1,^[^ ^43^ ^]^ the ligand‐binding pocket used for the docking will have a conformational change to adopt IGF1 (Figure 8L). Therefore, both PB‐020 and PPP likely block the binding of IGF1 to IGF1R by occupying the ligand‐binding pocket to competitively inhibit the activation of IGF1R.

## 3 Discussion

In this study, we report that the susceptibility of glioma cells to IGF1R targeting is determined not only by its oncogenic state, but also by its cell identity/state within the hierarchy of tumor development. Along the differentiation route of NSCs to more differentiated progenitors such as OPCs, distinct cell types positioned on the developmental hierarchy respond differently to IGF1R upon acquiring the oncogenic insults. Deactivating the IGF1R mainly impacts on mutant OPCs but minimally affects the growth of normal adult OPCs or NSCs. Molecularly, high sensitivity of mutant and cancer cells with the OPC feature toward IGF1R targeting likely stems from the intimate coupling between IGF1R and PDGFR*α*, as the coexpression of both RTKs largely occurs in OPCs in the brain.

Recent studies suggest that tumor OPCs widely exist in human gliomas.^[^ ^9^, ^25^, ^27^, ^28^, ^29^, ^30^, ^31^, ^32^ ^]^ These cells have been reported to contribute to the proliferation pool and may exhibit distinct migration/infiltration behaviors in vivo.^[^ ^26^, ^28^, ^30^ ^]^ Our scRNA‐seq and grafting studies suggest tumor OPCs residue at the apex of the developmental hierarchy and likely contribute as the major proliferation pool in mouse gliomas with OPC as the cell‐of‐origin. This conclusion can be recapitulated at least in some human GBMs (this study and^[^ ^25^ ^]^). Therefore, OPC‐related cells, in addition to playing roles in gliomagenesis, likely represent an important target for glioma the treatment of some gliomas. This notion is supported by a recent study that tumor OPCs existed as the cancer stem cells in human GBMs and may function to resist to therapy.^[^ ^44^ ^]^ More work should be done to further stratify GBMs to determine the suitable patients with the best prognosis with IGF1R targeted therapy. It would be highly interesting to investigate whether those with glial tumors where the cells having OPC identities constitute the majority of the tumor mass, such as proneural subtype of GBM, oligodendrogliomas, and DIPGs, would particularly benefit from IGF1R targeted therapy.

One interesting topic in the future is to identify the in vivo cellular source of IGF1. Given the establishment of the importance of IGF1R axis in gliomagenesis and TICs maintenance, at least the ones related to OPCs, in the current study, interruption of the communication from the ligand side can be a novel strategy for IGF1R‐based anti‐glioma therapy. Indeed, a recent work suggest that tumor associated microglia (TAM) have been reported to be one source of IGF1.^[^ ^16^ ^]^ Interestingly, subtype of neurons in the brain and hepatocytes in liver also express IGF1.^[^ ^45^, ^46^ ^]^ Dissection the individual roles of these cellular sources in gliomagenesis and the self‐renewal of glioma TICs are currently undergoing.

Clinical trials targeting IGF1R in patients with advanced solid tumors have been described.^[^ ^47^, ^48^, ^49^, ^50^ ^]^ While conflicting results were sometimes encountered, some patients exhibited profound responses to IGF1R inhibitor monotherapy, supporting the notion that IGF1R‐targeting is tumor and cellular/oncogenic context‐dependent. Given the poor ability of currently available IGF1R inhibitors to penetrate the brain, clinical trials of IGF1R targeting in gliomas lag behind those focusing on other solid tumors. Despite our studies on the mouse model suggesting a marginal, if any, brain‐penetration capacity of PPP, the lead molecule from which PB‐020 was developed (Figure 8), a recent and thus far the only phase‐I clinical trial shows that PPP was well tolerated and demonstrated some ability to prolong survival of a small number of GBM patients.^[^ ^23^ ^]^

One explanation for this discrepancy is that PPP may partially inhibit gliomas by suppressing angiogenesis. In fact, our scRNA‐seq data revealed that endothelial cells in glioma strongly expressed IGF1R, which could be the target of IGF1R antagonists. Supporting this speculation, Zamykal et al. reported that IGF1R neutralizing antibody may inhibit the intracranial growth of U87MG by targeting the growth of blood vessels.^[^ ^21^ ^]^ Therefore, clinical administration of BBB‐penetrable IGF1R inhibitor is expected to have multiple anti‐glioma effects, both cell‐autonomously and noncell autonomously. The gain of the BBB‐penetrating capacity and better pharmacokinetic profile position PB‐020 as a putative anti‐glioma therapeutic agent, either alone or in combination with other therapies. Comprehensive examinations by using variable subtypes of human gliomas should be further performed and additional modifications might be expected before moving PB‐020 to clinical trials.

## 4 Experimental Section

##### Ethics

All animal procedures followed the animal care guidelines approved by the Institutional Animal Care and Use Committee of Zhejiang University School of Medicine. All human glioma samples in this study were received from patients with written informed consents; and the protocol was approved by the ethics committee of Zhejiang University School of Medicine.

##### Antibodies and Key Reagents

Information for all antibodies and key reagents can be found in Table S1, Supporting Information.

##### Mouse Models

Following strains were used to build up the genetic mouse models in this study: *NG2‐Cre^ER^*, *p53KO*, *Rosa‐tdTomato* (Ai9, stock no.007909, JAX), *NF1flox*, *p53flox*, *IGF1Rflox* (stock no.012251, JAX), *Nestin‐Cre^ER^*, TG11ML (stock no.022977, JAX), GT11ML (stock no.022976, JAX), hGFAP‐Cre (stock no. 004600, JAX), and *NG2‐Cre* (stock no. 008533, JAX).

##### Human Samples

The detailed information of human subjects refers as to Table S2, Supporting Information. Fresh glioma tissues were directly dissected from surgery and processed within 8 h.

##### Cell Lines

Glioma cell lines #1483 and #1877 were derived from the CKO_NG2‐Cre^ER^ mouse model. Cell line #3841 was from the MADM‐hGFAP‐Cre mouse model. #1920 cell line was from the CKO_NG2‐Cre^ER^_IGF1R (flox/flox) mouse model. #4612 cell line was derived from glioma mouse model generated by PB transposon‐based CRISPR‐cas9 system to knock out *Trp53*, *Rb1*, and *Pten* through neonatal electroporation. Human GBM cell lines were established from freshly resected tumor tissues of GBM patients during surgery. All human glioma cell lines (#H2, #H5, #H63) used in this paper were cultured in complete media with EGF and FGF. The MEF cells were derived from the mouse embryos with desired genotypes at the age of Embryonic (E) day E13.5. The WT MEFs and the Trp53^−/−^; *NF1* ^−/−^ MEF cell lines were established from the WT_NG2‐Cre^ER^ and the CKO_NG2‐Cre^ER^ models, respectively. *Trp53/NF1* mutant OPCs were enriched from the neonatal (P8) CKO_NG2‐Cre model by anti‐O4 immunopanning. The detailed procedures to preparation and maintenance of cell lines are described in the Supporting Information.

##### Human Tissue Preparation and Histology

Fresh human glioma tissues were divided into two parts. One part was directly immersed into 30% formalin and subjected to formalin‐fixation‐paraffin‐embedding preparation. The other part was transported to the laboratory in ice‐cold DMEM supplemented with 1% penicillin/streptomycin. This second part of tumor tissue was further divided into three fractions: one was diced into small chunks and gone through fixation and O.C.T. embedding as previously described^[^ ^29^ ^]^; the second fraction was snap‐frozen in liquid nitrogen for RNA‐sequencing and qPCR; the third one was digested by papain into single cells and used for primary culture or immunopanning.

##### Immunofluorescence Histological Analyses

All mouse frozen tissues were cryosectioned into 20 µm thickness and refixed into ice‐cold 2% PFA at room temperature for 15 min. The protocol of immunostaining for human tissue sections refers to previous studies by the authors.^[^ ^7^, ^29^ ^]^ For human tissue sections, 0.3% Sudan Black B was used to quench the autofluorescence.

##### Cell Culture

Mouse glioma cell lines were maintained in the mouse glioma cell culture complete media, which contained neurobasal media, l‐Glutamine, penicillin/streptomycin, sodium pyruvate, d‐biotin, gentamycin, trace element B, B27 minus VA. The cell lines were cultured in T25 flasks precoated with poly‐d‐lysine. All human GBM cell lines were maintained in the human GBM complete media, which contained Neurobasal media, l‐glutamine, penicillin/streptomycin, sodium pyruvate, d‐biotin, gentamycin, trace elements B, B‐27 minus VA, transferrin, BSA, putrescine, progesterone, sodium selenite, insulin, and GFs as indicated. DBTGR /KNS‐81 were cultured in DMEM, supplemented with penicillin/streptomycin and 10% FBS. U87MG was cultured in DMEM, supplemented with penicillin/streptomycin, 10% FBS, sodium pyruvate, MEM nonessential amino acids solution. Culture media was half changed every week and passaged as routine. MEF cell line was prepared from E13.5 embryos with proper genotype. The torso without head, arms, legs, and viscera was minced and digested by TrypLE, and dissociated into single cells before cultured and maintained in DMEM, supplemented with penicillin/streptomycin and 10% FBS.

##### Immunopanning

The dissociated human GBM cells were briefly cultured in the complete media (without GF) at 37 °C 5% CO_2_ for 12–14 h before immunopanning. Overnight‐cultured GBM cells were trypsinized with prewarmed TypLE and washed with DPBS (w/o Ca^2+^ and Mg^2+^). The cell palette was washed and resuspended into the panning buffer (DPBS with Ca^2+^ and Mg^2+^, 0.02%BSA) and transferred to Goat Anti‐Mouse IgG (Jackson Immuno Research, #115‐005‐003)‐coated plate at room temperature for 8 min. The supernatant was then transferred to the prewashed anti‐CD140*α* antibody (BD Pharmingen, #556001) coated plate and incubated at room temperature for 15 min. The enriched tumor OPCs were collected from the plate by TrpLE. Immunopanning of mouse neonatal OPCs were performed as previously described by using anti‐O4 antibody.^[^ ^51^ ^]^ The cells were maintained in PDGFAA and FGF (20 ng mL^−1^ each) until used.

##### Flow Cytometry Analysis and Cell Sorting

Dissociated cells were incubated with desired antibodies for 30 min at room temperature followed by FACS analysis or sorting on CytoFLEX LX or BD FACSAria II and Beckman moflo Astrios EQ cell sorter .The antibodies used for FACS are: PE‐conjugated Mouse Anti‐Human CD221 (BD, #555999), Alexa FluorR 647‐conjugated Mouse Anti‐Human CD140*α* (BD, #562798), APC‐conjugated IGF1R (Abcam, #ab225298); APC‐conjugated Mouse PDGFR*α* (BD, #FAB1062A); PE‐conjugated anti‐human PDGFR*α* (BD, #FAB1264P); APC‐conjugated anti‐Human PDGFR*α* (BD, #FAB1264A).

##### Tumor sphere Assay

1000 or 3000 cells were seeded into each well of the 96‐well plate and cultured in 0.1 mL mouse glioma cell basal media, which contained Neurobasal media, l‐glutamine, penicillin/streptomycin, and B27 minus insulin. Cells were cultured for 4–7 days before analyzed. Four wells were repeated for each condition. For human cells, 3000 cells in 100 µL GBM basal media supplemented with GFs and/or inhibitors were seeded in each well of the 96‐well plate. Four wells for each condition.

##### Isolation and Culture of Mouse Adult NSCs

The lateral SVZ of the mice (from 2 to 5mice) with desired genotypes was micro‐dissected and single cell suspension was prepared using the Neural Tissue Dissociation kit with Papain (Miltenyi, #130‐092‐628). Dissociated cells were transferred in Neurobasal A/B27 media supplemented with 1% l‐glutamine, 2 mg mL^−1^ of heparin, 20 ng mL^−1^ of human FGF (Peprotech, #100‐18‐B) and 20 ng mL^−1^ of human EGF (Peprotech, #AF‐100‐15). Cells were not used for longer than 6 passages.

##### Differentiation of Adult Mouse NSCs

Adult neural stem cells (NSCs, ≈3000 cells) were seeded on PDL‐coated 96 wells plate in differentiation medium consisted of Neurobasal A/B27, 1% l‐glutamine, heparin, FGF2, PDGFAA, and 2%FBS for 9–11 days, 1/2 media with fresh growth factors were regularly replaced every 3–4 days.

##### Co‐IP Assay

Mouse tumor OPCs or HEK293FT cells transfected with the plasmids were lysed with Cell lysis buffer for Western and IP (Beyotime, #P0013) supplemented with protease inhibitor (Roche, #21287100) and phosphatase inhibitor (Roche, #26920800). Lysates were precleared by mouse IgG (Abmart, #B30010S) and Protein A/G PLUS‐Agarose (Santa cruz, #sc‐2003). The beads were pelleted by centrifugation at 2500 rpm for 5 min at 4 °C, and supernatant was used for IP experiments.

##### Western Blots

Tissues or cultured cells were lysed in the cold RIPA buffer supplemented with the protease inhibitor cocktail and phosphatase inhibitor cocktail tablets. Protein samples were then subjected to the SDS‐PAGE by electrophoresis and transferred onto polyvinylidene difluoride membranes as routine procedure. WB bands were detected with secondary antibodies coupled to horseradish peroxidase by using chemiluminescence methods with the ECL detection kit.

##### Tamoxifen and BrdU administration

Tamoxifen citrate (TAM) was orally administrated via gavage at a concentration of 200 mg kg^−1^ (prepared in ddH_2_O) body weight for 5 days (1 dose per day) at P31–35 to induce recombination. For repeated administration of TAM given in Figure 2I, TAM was intermittently given as a 15day‐cycle until the mice showed symptoms. BrdU was administered by intraperitoneal injection (i.p.) at a concentration of 50 mg kg^−1^ (prepared in 1× PBS) body weight for 8 days (1 dose per day). Mice were sacrificed 2 h after the last injection of BrdU.

##### Intracranial Grafting of Mouse and Human Glioma Cells

Mouse (10 000 cells per mouse) or human glioma cells (100 000–300 000 cells per mouse) were suspended into Neurobasal media as a density of 5 000 000 or 50 000 000 viable cells mL^−1^ and grafted into the brains of NOD‐SCID mice. After the surgery, the mice were monitored daily and sacrificed at the onset of neurological symptoms, or based on the designed time points. The coordinates of grafting (measured according to bregma) was 1 mm anterior, 1 mm lateral, and 3.5 mm deep.

##### Synthesis of PB‐020 [(5R,5aS,8aR,9R)‐2,2‐difluoro‐9‐hydroxy‐5‐(3,4,5‐tris(methoxy‐d3)phenyl)‐5,8,8a,9‐tetrahydrofur′[3′,4′:6,7]naphtho[2,3‐d][1,3]dioxol‐6(5aH)‐one‐9‐d]

The detailed method for compound synthesis is provided in the Supporting Information. Briefly, to a stirred solution of intermediate **1.6** (400 mg, 0.89 mmol, 1.0 eq.) in methanol (10 mL), NaBD_4_ (37.5 mg, 0.89 mmol, 1.0 eq.) was added in portions, and the resultant mixture was stirred for 4 h. The reaction was quenched with water, and was concentrated to dryness. The obtained residue was diluted with water, and extracted with EtOAc. The combined organic phase was washed with brine, dried over MgSO_4_, and concentrated to dryness. The obtained residue was recrystallized from PE/EtOAc to yield compound **PB‐009** (140 mg, 35% yield) as white solid. Compound **PB‐019** was synthesized in a similar way as compound **PB‐009**, using 3,4,5‐tris (trideuteriomethoxy) benzaldehyde as the starting material. Compound **PB‐020** was prepared by chiral separation of compound **PB‐019** on chiral column chromatography.

##### Evaluation for BBB Penetration

The mice were dosed by gavage with 50 mg kg^−1^ of PPP, PB‐004, PB‐016, or PB‐020 dissolved in DMSO/corn oil (1:9 v/v) solution. At predosing (0 h) and 1, 3, 6, 12, and 24 h post‐dosing time points, the blood samples (approximately 1 mL) were collected from abdominal aorta, and the plasma samples were subsequently transferred into centrifuge tubes by centrifuging for 5 min at 4000 rpm. The brain tissues were collected and immediately frozen in liquid nitrogen. Approximately 0.3 g brain tissues were evenly divided into two parts. One part was homogenized in acetonitrile for HPLC assay, and the other part was homogenized in RIPA solution for immunoblotting. For HPLC‐UV assay, samples were subjected to the HPLC system (Hitachi Chromaster, Japan). The tested compounds present in the plasma or brain samples were identified by matching the peaks of retention time for the reference compounds, and their concentrations were determined by measuring the areas under the peaks.

##### Single Cell Transcriptome Sequencing

Single cells were processed through the 10× Chromium 5’ (for the primary tumor sample) or 3’ (for all cell lines) Single Cell Platform using the Chromium Single Cell 5’ or 3’ Reagent Kits v3, Gel Bead and Chip Kits (10× Genomics, Pleasanton, CA), following the manufacturer's protocol (Illumina). In this study, tdTomato gene inherent was added in the mouse model gene list to obtain the final matrics by modifying the reference genome. The matrix was separated by the cell type judgment result obtained by the cellranger. Cells with high quality were selected with the following criteria: 1) Genes detected in < 3 cells were removed; 2) cells with unique molecular identified (UMI) ≤ 150 or ≥ 4500 were removed. 3) Cells with ≥ 20% mitochondrial counts were removed. Normalization was performed using Seurat.^[^ ^52^ ^]^ R package Seurat was used for unsupervised clustering. Monocle was used to construct single‐cell pseudo‐time trajectories.^[^ ^53^ ^]^

##### Bulk Transcriptome Sequencing

Human GBM tissues and four pairs of panned and supernatant GBM fractions were analyzed by RNA‐Seq (Table S5, Supporting Information). The sequencing libraries of the four pairs of panned and supernatant human glioma cells were built based on Ovation Universal RNA‐Seq System and the seven human GBM tissues were prepared by the Truseq Stranded mRNA LT Kit. The libraries were sequenced on Illumina Hiseq platforms by Shanghai Personal Biotechnology Co., Ltd. The Genepattern online tool (https://genepattern.broadinstitute.org/) was used to calculate ssGSEA. The subtractive ssGSEA enrichment scores between panned and supernatant fractions from each sample were presented as the difference of the ssGSEA scores for the four subtypes.

##### GO‐Term Analysis

Differential expression genes (DEGs, log2‐fold change > 1, *p* < 0.05) were screened by DESeq in each pair of immune‐panned cells and supernatant cells. Those DEGs were then used for the GO Enrichment Analysis online (http://geneontology.org/) to generate related enriched pathway.

##### Imaging Collection and Processing

All confocal images were collected by an Olympus FV‐3000 inverted confocal microscopy and analyzed with Olympus Fluoview 1000 software. All sphere culture images in cell culture were collected using an Olympus CKX53 microscope and processed by Image J.

##### Statistics and Reproducibility

Survival curves were analyzed and presented as the Kaplan–Meier survival curves. The comparison between curves were performed by the log rank test. Student's *t*‐test was used to determine the statistical significance of all quantification. Values of *p* < 0.05 were considered as significant. Statistical analysis was performed using GraphPad Prism 5. Data were presented as mean ± SEM. Each in vitro experiment has been independently repeated at least twice. No statistical methods were used to predetermine sample sizes, but the sample sizes herein were similar to those reported in previous publications.^[^ ^5^, ^54^, ^55^ ^]^ Data distribution was assumed to be normal, but this was not formally tested. Randomization of animal studies was used in the data analysis. Data collection and analysis were not performed in a blinded manner to the conditions of the experiments. Detailed quantification schemes in this paper can be found in the Supporting Information.

## Conflict of Interest

J.L., M.Y., and L.W. are current employees of PharmaBlock Sciences (Nanjing), Inc.

## Author contributions

A.T., B.K., and B.L. contributed equally to this work. C.L. conceptualized the project. A.T. and C.L. initiated this study. C.L. supervised the study and conducted quality control on the data. C.L. wrote the manuscript. A.T., B.K., B.Q., W.J., F.S., Q.G., W.W., R.L., and P.C. performed the experiments. A.T. performed most histological experiments with the inputs from B.Q., F.S. and Q.G., Y.J.W., J.L., and M.Y. performed design and chemical synthesis of PPP analogs (PB‐020 series), B.K. and Y.J.W. conducted cellular, biochemical and in vivo analyses for PPP analogs (PB‐020 series), L.W. conducted molecular docking for PPP analogs (PB‐020 series). B.Q. performed the WB experiments. W.J., Q.G., and F.S. performed the cell culture and the RNA‐seq experiments. W.J. analyzed the RNA‐seq results. B.L. performed the pathology. J.Z. provided human tumor samples. W.W. maintained the transgenic animals. R.L. constructed all DNA vectors. A.T., Q.G., and W.J. performed statistical analysis of the results. All authors contributed to the interpretation of the results. C.L. and A.T. assembled the figures. All authors discussed results and commented on the manuscripts.

## Supporting information

Supporting Information

Supplemental Table 1

Supplemental Table 2

Supplemental Table 3

Supplemental Table 4

Supplemental Table 5

Supplemental Table 6

Supplemental Table 7
