# Supplementary material for: Oncogenic State and Cell Identity Combinatorially Dictate the Susceptibility of Cells within Glioma Development Hierarchy to IGF1R Targeting
Source: Adv Sci (Weinh). 2020 Oct 1;7(21):2001724. doi: 10.1002/advs.202001724 (PMC7610337; doi:10.1002/advs.202001724)
Supplement: Supplementary file 1 — Supporting Information [file ADVS-7-2001724-s001.pdf]

WILEY-VCH

© 2020 Wiley-VCH GmbH

ADVANCED SCIENCE
Open Access

Supporting Information

for *Adv. Sci.*, DOI: 10.1002/advs202001724

Oncogenic State and Cell Identity Combinatorially Dictate the Susceptibility of Cells within Glioma Development Hierarchy to IGF1R Targeting

*Anhao Tian, Bo Kang, Baizhou Li, Biying Qiu, Wenhong Jiang, Fangjie Shao, Qingqing Gao, Rui Liu, Chengwei Cai, Rui Jing, Wei Wang, Pengxiang Chen, Qinghui Liang, Lili Bao, Jianghong Man, Yan Wang, Yu Shi, Jin Li, Minmin Yang, Lisha Wang, Jianmin Zhang, Simon Hippenmeyer, Junming Zhu, Xiuwu Bian, Ying-Jie Wang and Chong Liu**

Supporting Information

Oncogenic State and Cell Identity Combinatorially Dictate the Susceptibility of Cells within Glioma Development Hierarchy to IGF1R Targeting

*Anhao Tian, Bo Kang, Baizhou Li, Biying Qiu, Wenhong Jiang, Fangjie Shao, Qingqing Gao, Rui Liu, Chengwei Cai, Rui Jing, Wei Wang, Pengxiang Chen, Qinghui Liang, Lili Bao, Jianghong Man, Yan Wang, Yu Shi, Jin Li, Minmin Yang, Lisha Wang, Jianmin Zhang, Simon Hippenmeyer, Junming Zhu, Xiuwu Bian, Ying-Jie Wang and Chong Liu **

Supporting information includes **supplementary materials and methods**, 12 supplementary figures (**Figure S1-S12**), and 7 supplementary tables (**Table S1-S7**).

Supplementary Figures:

Figure S1

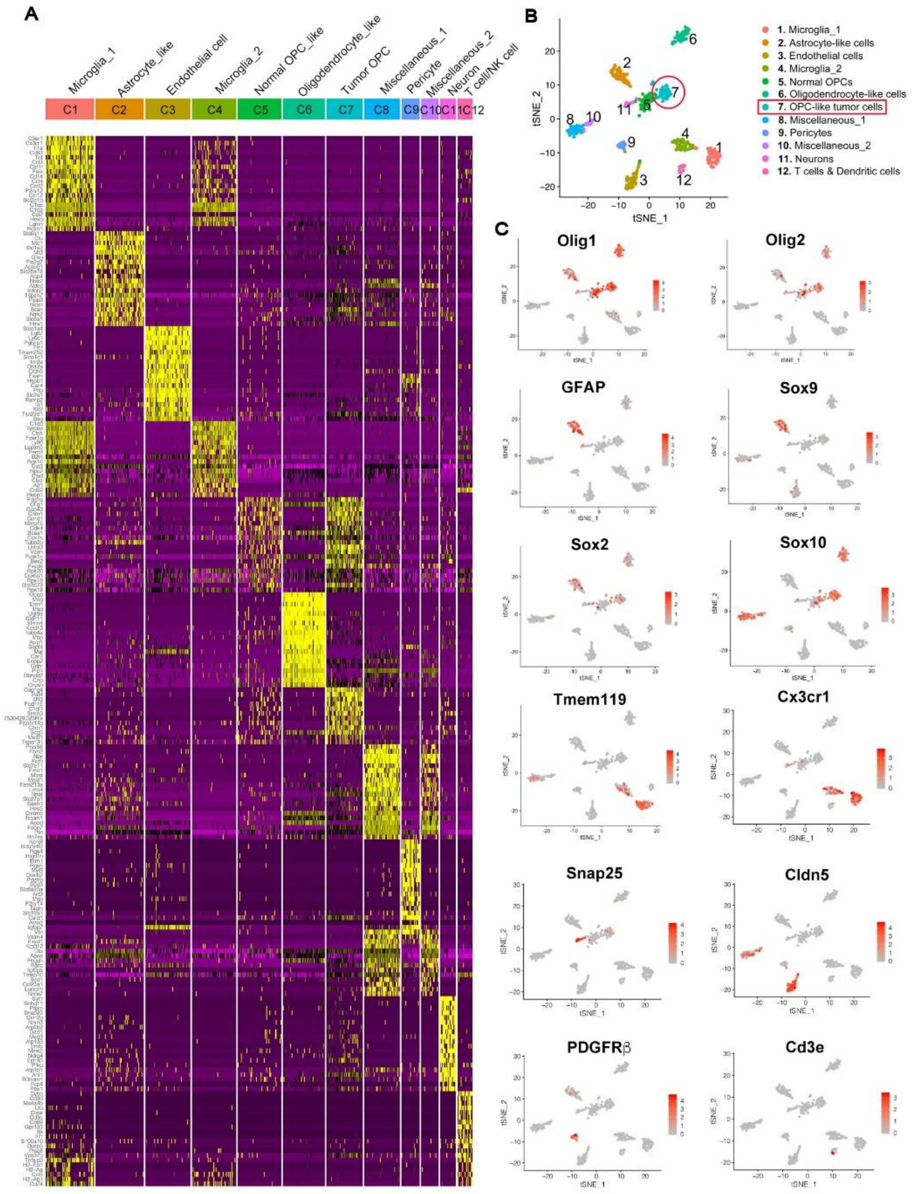

Figure S1. Heatmap Showing clustering analysis of all single cells from the CKO_NG2-Cre^{ER} tumor.

(A) Top 20 most variable genes are listed on the left. Same color codes were used as those in **Figure 1C-D**.

(B) The clustering of the cells from a CKO-NG2-Cre^{ER} tumor (same as that in **Figure 1C**).

(C) The expression of some typical marker genes on the tSNE map in (B).

Figure S2

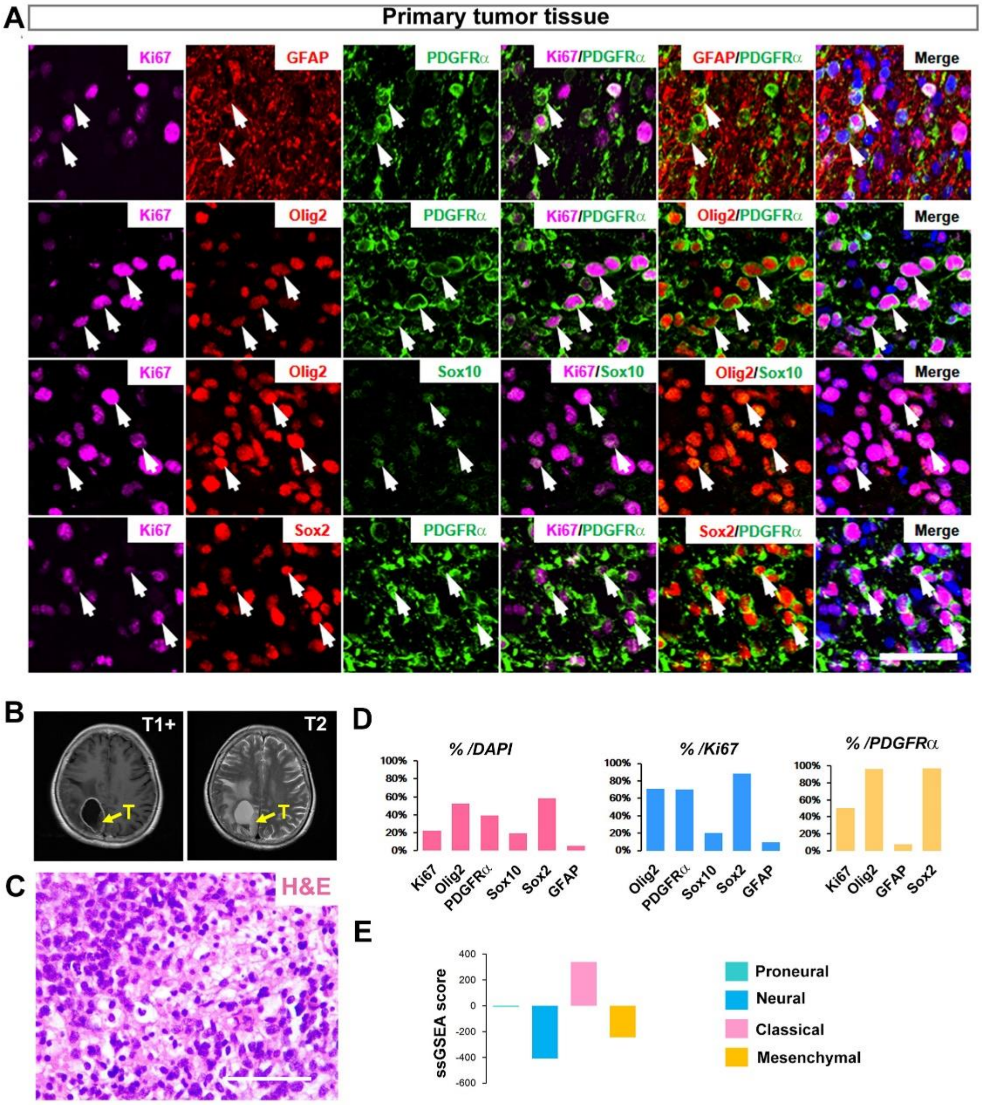

Figure S2. A representative example showing that tumor OPCs are present in human glioblastoma and serve as the major proliferating pool in the tumor mass.

(A) Representative images of a frozen human GBM sample (#H31) co-stained with the markers as indicated. Please refer to **Table S2** (**Supporting Information**) for detailed pathological information about this patient. The arrows point to the cells with identifiable marker co-localization. Scale bars: 50 µm. This is the same tumor sample used in **Figure 6A**. Importantly, the similar results have been observed in multiple GBM and lower-grade human glioma samples, as shown in **Figure S8D (Supporting Information).**

(B) The T1 enhanced (T1+) and T2 phase of the MRI images from the GBM patient shown in (A). The arrows indicate the brain tumor.

(C) The H&E staining confirms the GBM pathology of this tumor sample. Scale bar: 100 μm.

(D) Quantitative analysis of the tumor cells expressing a combination of cellular and proliferation markers in this GBM sample.

(E) ssGSEA analysis stratifies this GBM sample into the classical subtype.

Figure S3

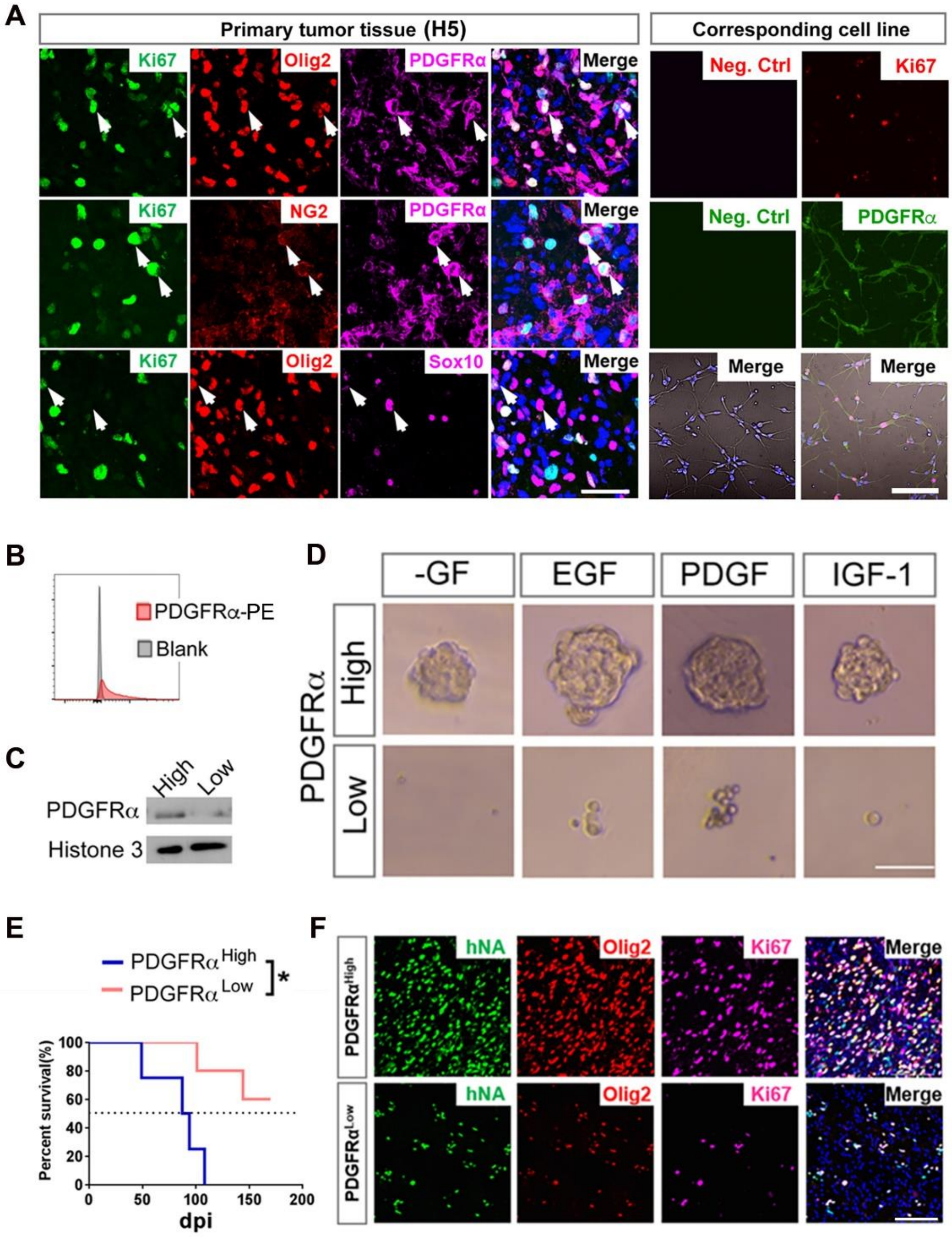

Figure S3. Human tumor OPCs can function as tumor initiating cells.

(A) Characterization of the primary tumor tissue and the corresponding cell line or the #H5 sample. This sample was used as the major human prototype in this study, including Western blots, grafting assay and PB-020 test. Scale bar: 50 μm in primary tumor tissue, 200 μm in corresponding cell line.

(B-D) The sphere assay (D) of human tumor OPCs (#H5) based on their surface PDGFRα expression (B). The expression of PDGFRα in high/low fraction was validated by Western Blots in (C). Scale bar: 100 μm in (D)

(E) The survival curves of NOD-SCID mice grafted with human tumor OPCs (#H5) based on their surface PDGFRα expression, N=4 mice for PDGFRα^{High} group and N=5 mice for PDGFRα^{Low} group, ** p<0.01..

(F) The histological analysis of the brain sections from the tumor mice in (E). Human tumor cells were visualized by their hNA expression. Scale bar: 100 μm.

Figure S4

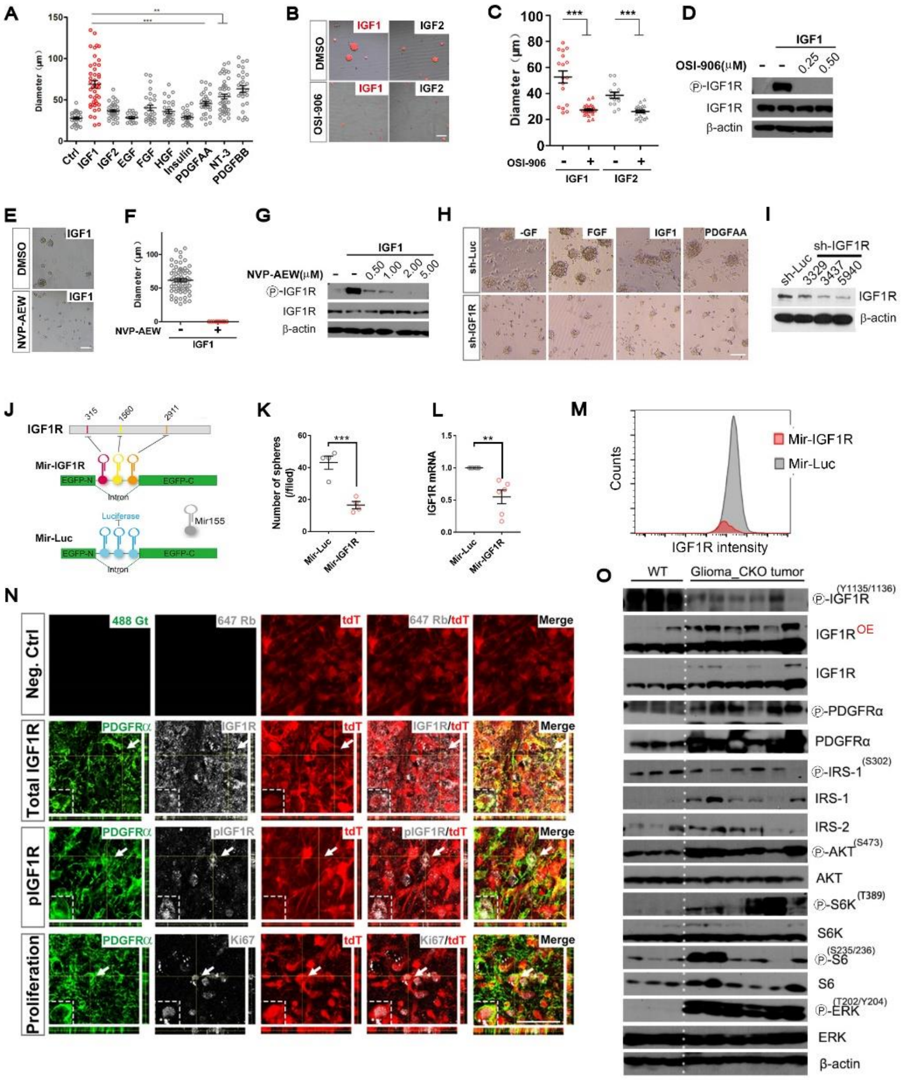

Figure S4. Tumor OPCs and IGF1R signaling.

(A) The quantification of a tumor sphere assay for OPC-originated tumor OPCs stimulated by the growth factors as indicated. This cell line was established from a CKO_NG2-Cre^{ER} tumor mouse different than that used in **Figure 2A-B**. All growth factors were provided as 10 ng/ml.

(B-C) Effect of the IGF1R inhibitors OSI-906 (0.5 µM) on IGF-stimulated tumor sphere formation. Scale bar: 100 µm in (B).

(D) The western blots (WB) confirm the ability of OSI-906 to inhibit IGF1R autophosphorylation.

(E-G) Effect of the IGF1R inhibitor NVP-AEW541 on IGF-stimulated tumor sphere formation. The western blots (WB) shown in (G) confirm the ability of these inhibitors to inhibit IGF1R activity. Scale bar: 100 μm in (E).

(H) Representative images of *in vitro* cultures of tumor OPCs transfected with the combination of three shRNA vectors that targeting distinct regions within the IGF1R encoding region. Notably, none of IGF1, FGF or PDGFAA could rescue the phenotype of IGF1R knockdown. The tumor cells were cultured on PDL-coated plates. Scale Bar: 100 μm.

(I) Western blots validating the IGF1R knockdown in those tumor cells in (H).

(J) Schematic of the Mir155 construct used to knock down endogenous mouse IGF1R. miRNA sequences against luciferase were used as the non-specific control. Of note, these miRNA sequences were inserted into the artificial beta-globin intron of an EGFP coding sequence to enable the visualization of transfected cells.

(K) miRNA155-based RNAi targeting the endogenous IGF1R suppressed the IGF1 stimulated sphere formation. The area per field in (K), 0.66 mm^2.

(L-M) Quantitative PCR (L) and FACS (M) validating the IGF1R knockdown by the Mir155-based RNAi targeting the IGF1R at the mRNA or the protein level.

(N) Representative immunofluorescence (IF) confocal images of the tumor sections from a Glioma_CKO_NG2-Cre^{ER} mouse model. Ki67 was used as the proliferation marker. The arrows indicate cells co-expressing markers. The insets show a zoom-in image of the indicated cells. Z-axis orthogonal views were provided to confirm the marker co-localization. Scale bar: 50 μm.

(O) Western blots showing expression of IGF1R/PDGFRα signaling proteins in tumor tissues directly isolated from the CKO_NG2-Cre^{ER} mouse model. FL, full length of IGF1R. B, beta-chain of IGF1R. OE, over-exposed.

Mean ± SEM. $^{**}P < 0.01$, $^{***}P < 0.001$.

Figure S5

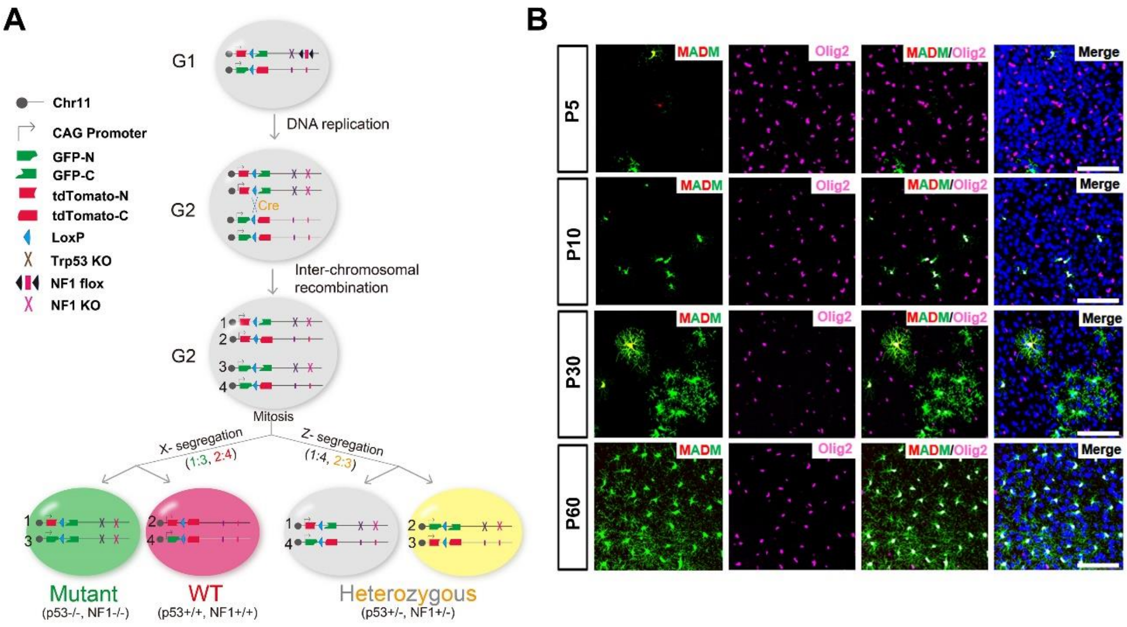

Figure S5. Working Scheme of MADM system.

(A) Schematic showing how the MADM system works. Shown here is the configuration for MADM-mutant model as shown in **Figure 4A**. NG2-Cre was used in the MADM models to label OPC-lineage cells.

(B) Representative images of the brain sections from the MADM-mutant model at the indicated developmental time points. Olig2 was used to mark all OPC-lineage cells. Scale bars: 50 μm.

Figure S6

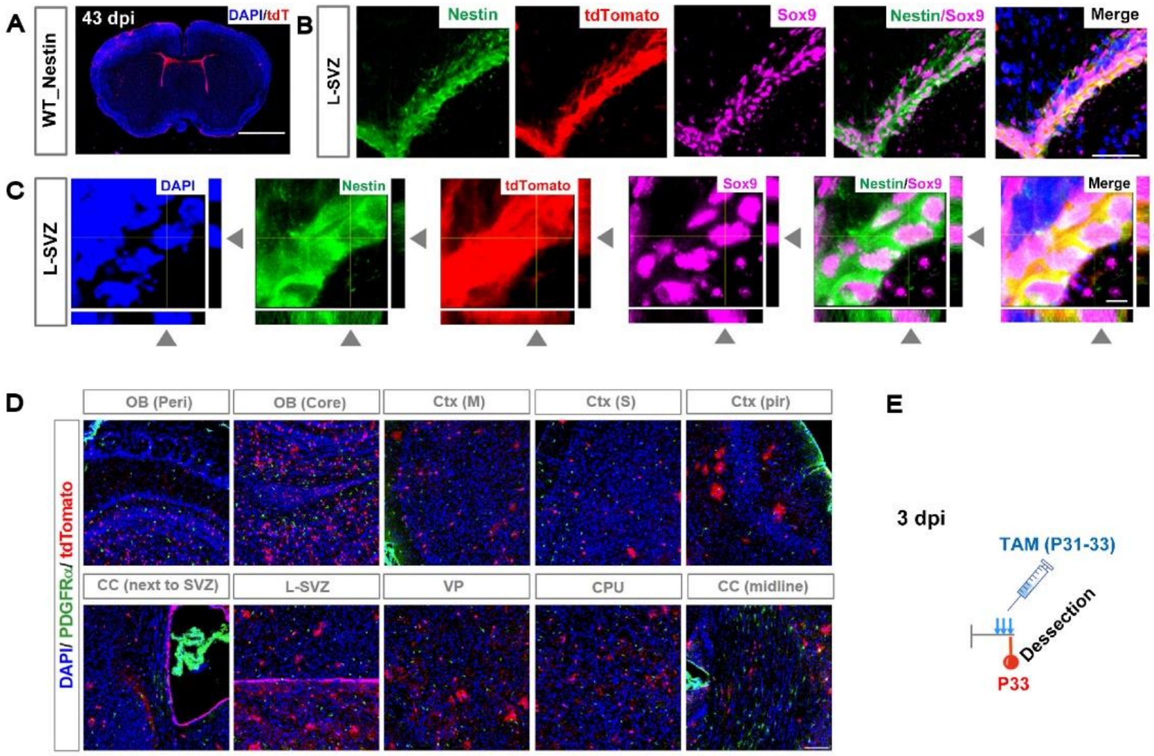

Figure S6. Characterization of the specificity of Nestin-Cre^{ER} transgene in targeting adult neural stem cells (NSCs) but does not directly targeting OPCs.

(A) Gross images of the brain section from a WT_Nestin-Cre^{ER} mouse model one month after tamoxifen treatment. The red fluorescence signal (tdTomato) visualizes the distribution of the targeted cells lining the lateral SVZ. DAPI was used to outline the brain structure. Scale bar: 2 mm.

(B-C) Zoom-in of images from (A) showing that tdTomato-labeled cells co-express Nestin and Sox9. An orthogonal view is provided in (C). The arrows indicate a cell expressing all of the indicated markers. Scale bars: 50 μm in (B), 5 μm in (C).

(D) Representative images of the different brain regions from a WT_Nestin-Cre^{ER} mouse model 3 days (3dpi) after tamoxifen treatment. No red cells co-localized with OPC marker PDGFRα. N=3 mice were examined. OB, olfactory bulb; Ctx (M), motor cortex; Ctx (pir), piriform cortex; CC, corpus callosum; VP, ventral pallidum; CPU, striatum. Please refer

Figure S7C (Supporting Information) for the locations of these regions in the mouse brain.

Scale bar: 100 μm.

(E) Schematic of the tamoxifen administration schedule in (D).

Figure S7

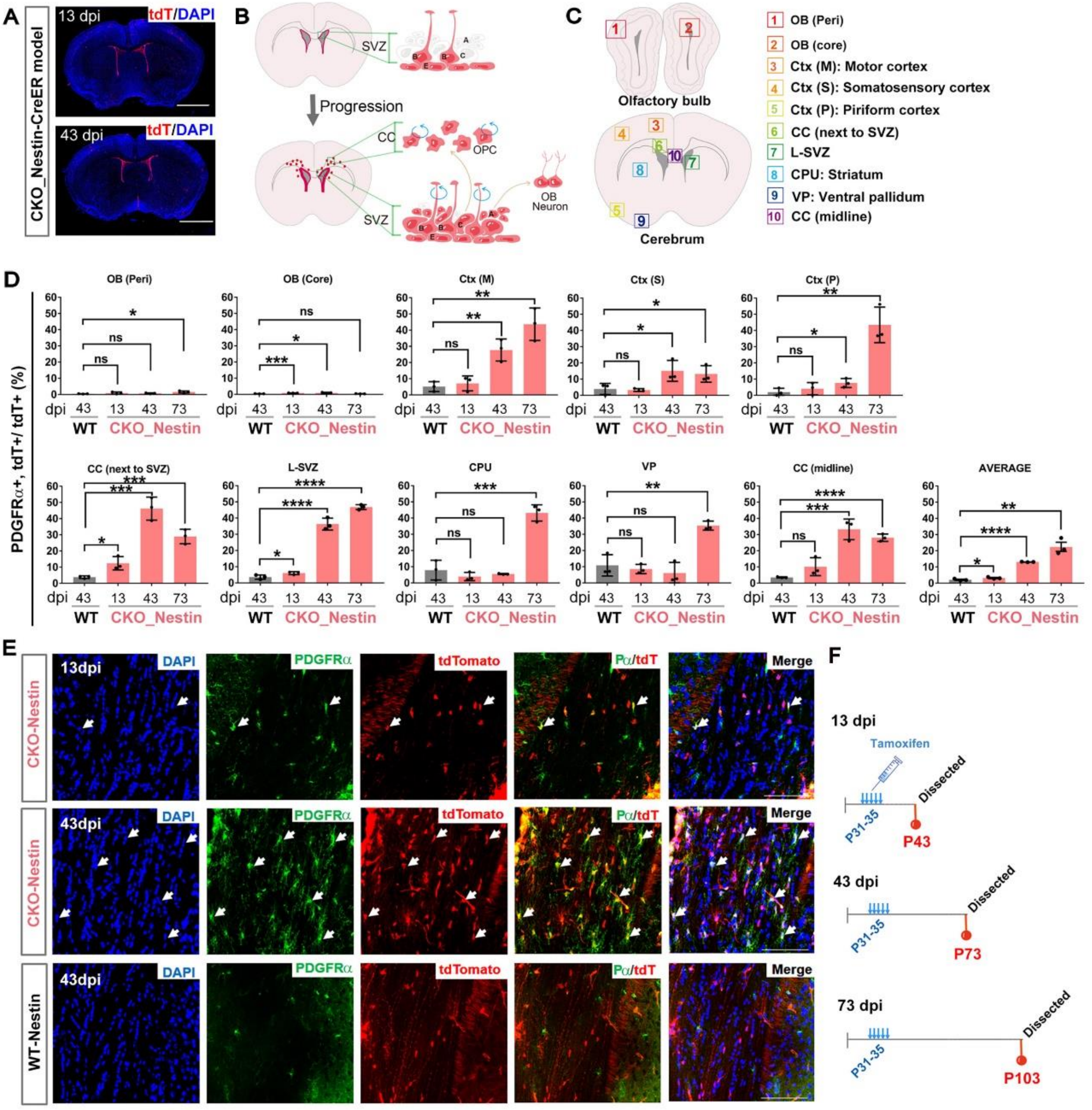

Figure S7. Adult NSCs preferentially generate OPCs after acquiring *Trp53* and *NF1* mutations.

(A) Gross images of brain sections from the CKO_Nestin-Cre^{ER} model analyzed at two time points after tamoxifen treatment. Scale bar: 2 mm.

(B) Schematic showing the differentiation pattern of adult NSCs after acquiring the initial mutations. E, ependymal cells.

(C) Schematic of the quantification scheme for the brain regions used in (D).

(D) The percentage of labeled mutant OPCs among all labeled mutant cells in multiple brain regions at three different time points after tamoxifen treatment. The WT_Nestin-Cre^{ER} model at 43 dpi was used as the reference. Data averaged across all these regions are provided on the right. N=3 mice for each group.

(E) Representative images of the brain sections from the mouse models indicated. The images were obtained from the CC region (region #6 in C). The arrows point to the cells co-expressing all of the markers indicated. Importantly, in the WT brain, adult NSCs rarely gave rise to OPCs even at 43 dpi. Scale Bar: 100 μm.

(F) Schematic of the tamoxifen administration schedule for the mouse models used in this figure.

Please refer to **Table S7 (Supporting Information)** for all raw quantification data. Mean ± SEM.* $P<0.05$, ** $P<0.01$, *** $P<0.001$, **** $P<0.0001$, ns, no significance.

Figure S8

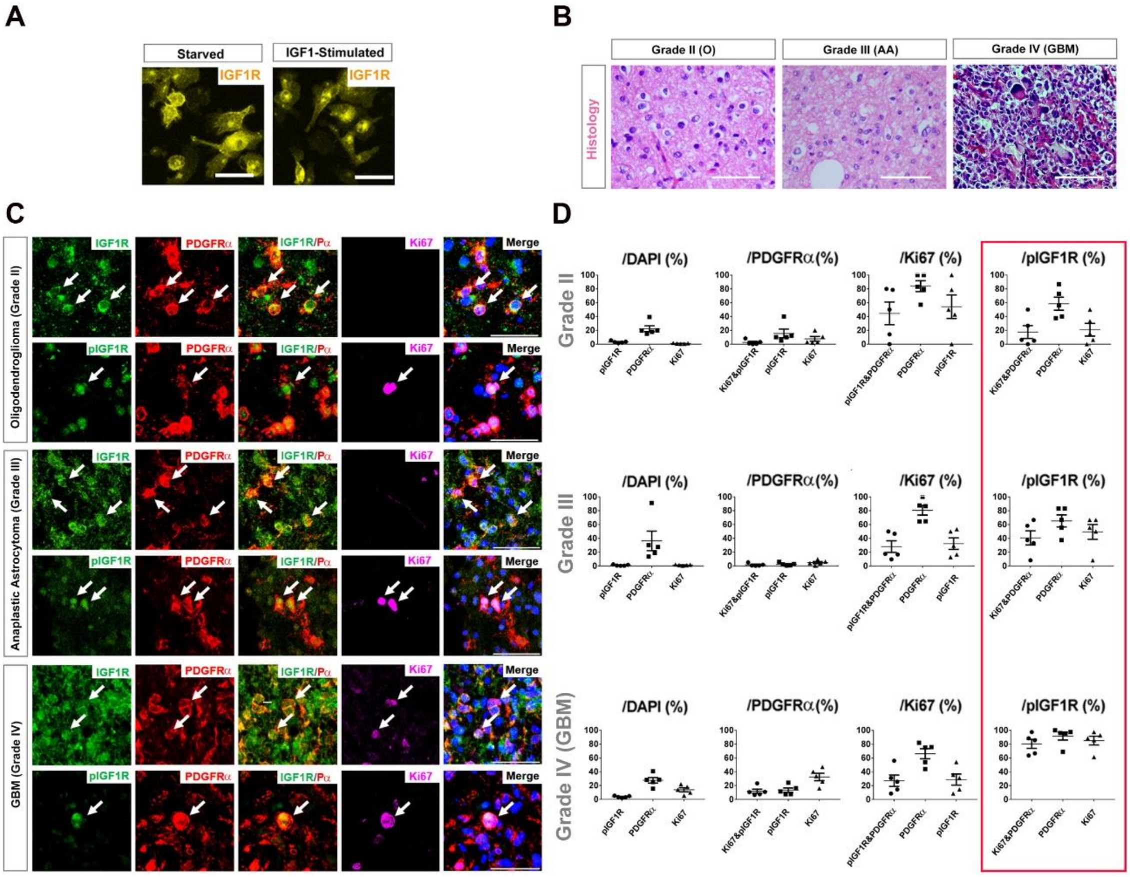

Figure S8. Representative cases to showing that IGF1R can be preferentially activated in proliferating tumor OPCs in human malignant gliomas.

(A) Confocal images of a primary human GBM cell line showing the translocation of the IGF1R from the cell membrane to the cytoplasm following IGF1 stimulation. The cells were stimulated with 20 ng/mL IGF1 for 8 hours. Scale bar: 50 μm.

(B) H&E staining confirms the pathological identities of the three samples in (C). Scale bar: 100 μm.

(C) Representative images of three frozen human glioma samples (each represents a different WHO grading) co-stained with the cellular markers indicated. The arrows indicate cells with identifiable marker co-localization. Scale bar: 50 μm.

(D) The quantification of the cells co-expressing indicated markers in multiple human glioma samples (N=5 for GBM, grade III and grade II), Mean ± SEM.

Figure S9

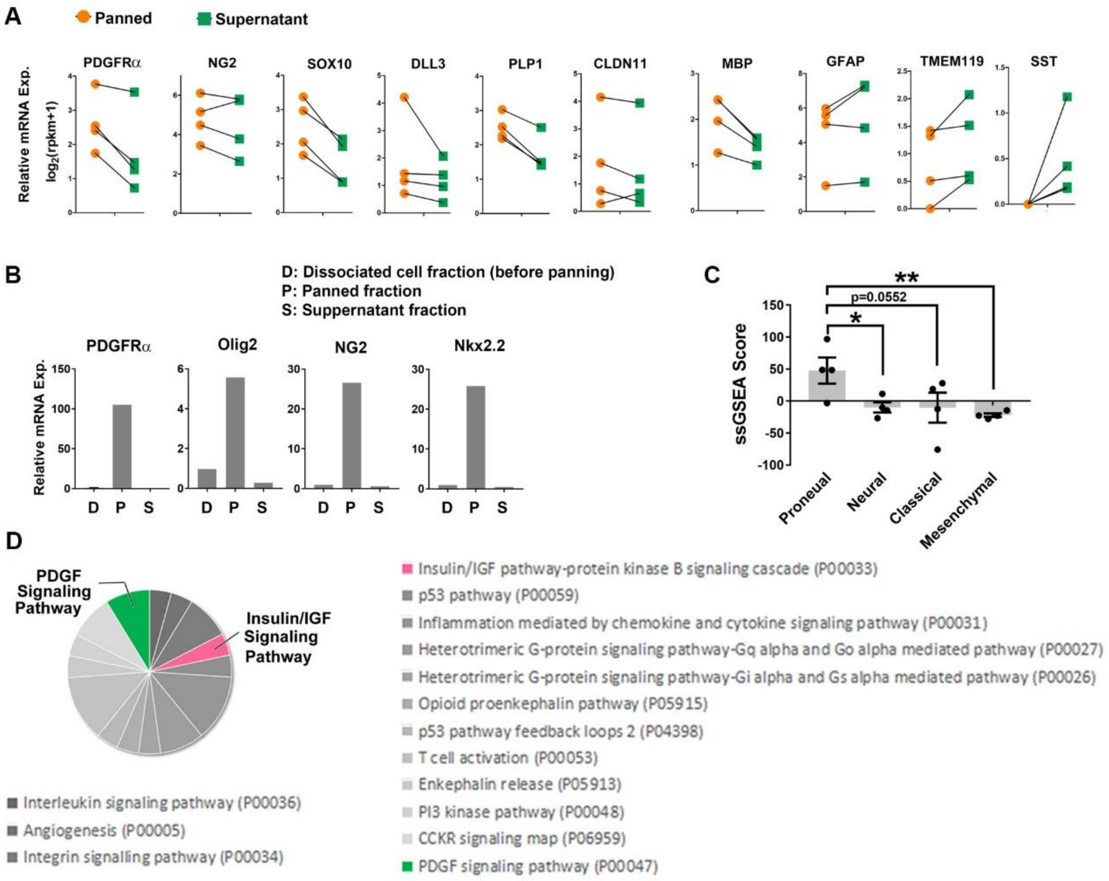

Figure S9. OPC-like identity of immune-panned human GBM cells.

(A) The RPKMs of indicated genes from an RNA-Seq analysis of immuno-panned vs. supernatant fractions from four human GBM samples. The immuno-panned and supernatant fractions from the same sample were paired (#H63, #H80, #H81, #H84).

(B) q-PCR validation of the enrichment of tumor OPCs from a fresh GBM sample.

(C) The subtractive ssGSEA enrichment score of the immuno-panned to supernatant fractions from four human GBM samples (#H63, #H80, #H81, #H84), * P<0.05, ** P<0.01.

(D) GO-term analysis of the genes showing > 2-fold up-regulation in the immuno-panned fraction compared to the supernatant fraction from the H63 GBM cell line. Similar enrichment of IGF and PDGF signaling can also be observed in other three samples analyzed.

Figure S10

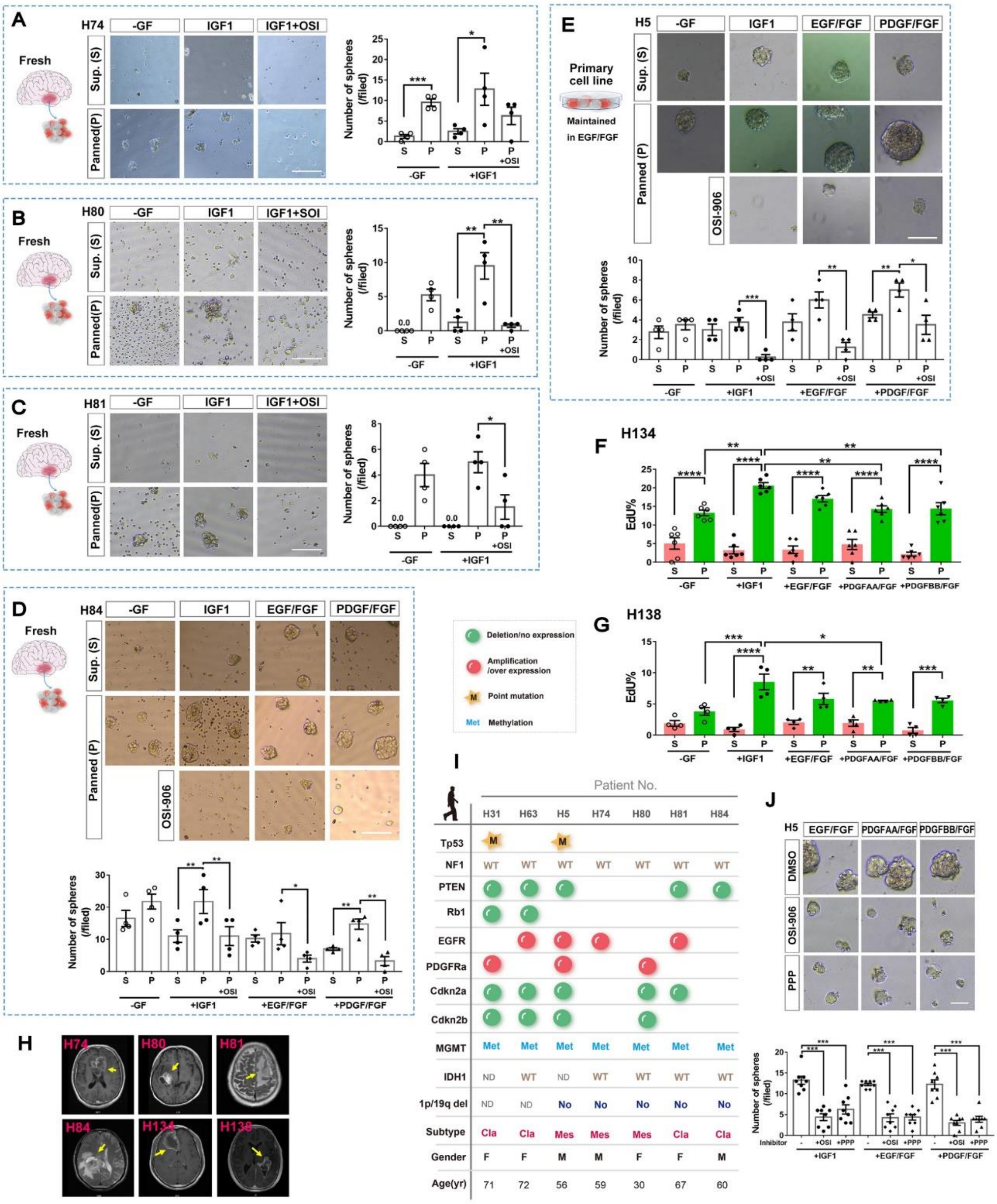

Figure S10. Human tumor OPCs tend to form spheres and are highly sensitive to IGF1R inhibition.

(A-E) Representative images and quantitation of the sphere assay performed on immuno-panned vs. supernatant fractions from four fresh human GBM samples (A-D) or a primary GBM cell line (E). The tumor OPCs were enriched using an immuno-panning approach. The

cell line in (E) was maintained in EGF/FGF before used for immuno-panning. The enrichment of tumor OPCs from the fresh samples was validated using an RNA-Seq analysis (as shown in **Figure S9, Supporting Information**). S: Supernatant fraction, P: Immuno-panned (tumor OPCs) fraction. OSI-906, 0.5 µM. Scale bars: 100 µm in (A-E).

(F-G) Proliferation rates of tumor OPCs from two GBM tissues. The cells were cultured as adherent culture. The proliferation rates were measured by EdU incorporation.

(H) The MRI images of the patients used in this figure. Arrows point to the tumor locations.

(I) Summary of the genetic information and the molecular classification of the GBM samples used in this study. Notably, none of the tumors contained *NF1* mutations, suggesting that aberrant oncogenic signaling circuits, rather than the *NF1* mutation *per se* is important for tumor OPCs to respond to IGF1R targeting. See also **Tables S2 (Supporting Information)** for information of molecular classification of all of these GBM samples. ND: No Determined.

(J) Both OSI-906 and PPP effectively suppressed sphere formation of human glioma cell line #H5 stimulated by multiple growth factors.

The area per field in (A-E), (F), and (J), 0.66 mm^2.

Mean ± SEM. *$P < 0.05$, **$P < 0.01$, ***$P < 0.001$, ****$P<0.0001$.

Figure S11

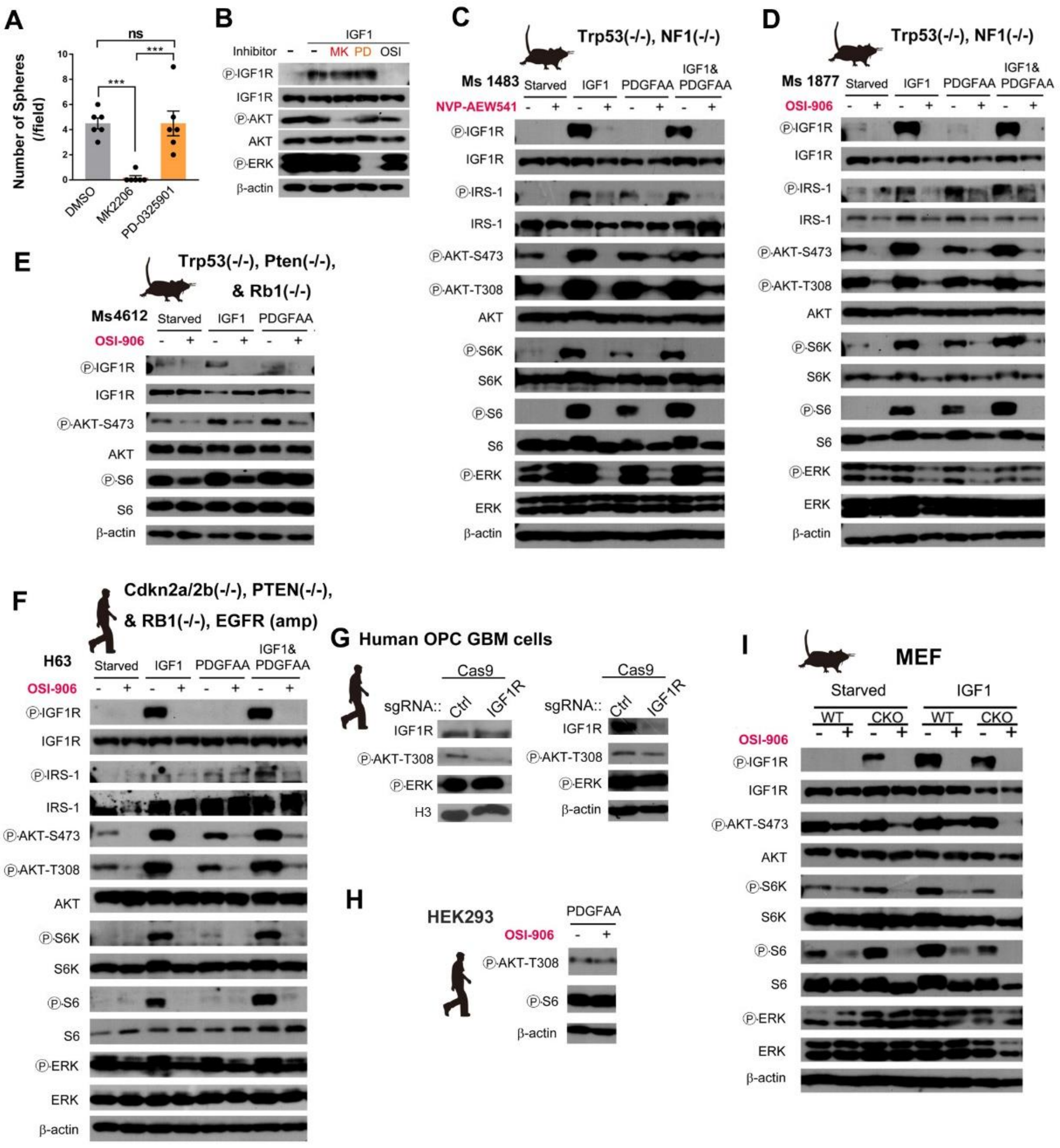

Figure S11. Characterization of IGF1R signaling in multiple mouse and human primary glioma cell lines.

(A) Sphere assay for mouse tumor OPCs treated with the indicated inhibitors. Spheres were formed in the presence of 10 ng/ml IGF1. Mean ± SEM. ***P < 0.001, ns, no significance.

(B) Western blots to validate the specificity of the inhibitors. MK, MK2206. PD, PD-0325901. OSI, OSI-906. The concentration for inhibitors: MK2206, 3 μM; PD-0325901, 1 μM; OSI-906, 0.5 μM.

(C) The same mouse glioma cell line as used in **Figure 7C** but treated with a different IGF1R inhibitor NVP-AEW541.

(D) An independent CKO_NG2-Cre^{ER} mouse glioma cell line was examined to confirm the result shown in **Figure 7C**.

(E) Similar results can be observed by using the mouse glioma cell line with *Trp53*, *Pten* and *Rb1* mutations. This cell line was established from a PB transposon-mediated, CRISPR-Cas9-based, somatic genetic knockout mouse model.

(F) The same observation can also be obtained using a primary human GBM cell line (#H63). This cell line was established from a human GBM stratified into the classical subtype. Please refer to **Figure S10I** and **Table S2 (Supporting Information)** for the pathological information about this cell line.

(G) Genetic knockout of IGF1R in two human tumor OPCs cell lines (#H63 and #H5 respectively) decreased the phosphorylation of AKT.

(H) OSI-906 failed to inhibit PDGFAA-elicited PI3K pathway in human HEK293 cells, suggesting that IGF1R-dependent activation of PI3K by PDGFAA depends on the cellular context.

(I) The response of WT and *Trp53/NF1* double mutant MEFs to IGF1 in the presence or absence of OSI-906. These results are well consistent with WT and precancerous mutant OPCs shown in **Figure 7D**.

For the inhibitor experiments, cells were starved overnight in basal media. After pre-treatment with inhibitors for 30 minutes, the cells were stimulated with IGF1, PDGFAA, or both (20 ng/mL of each) for 2 hours before being analyzed by western blotting. The concentration of inhibitors: OSI-906, 0.5 μM; NVP, 2 μM.

Figure S12

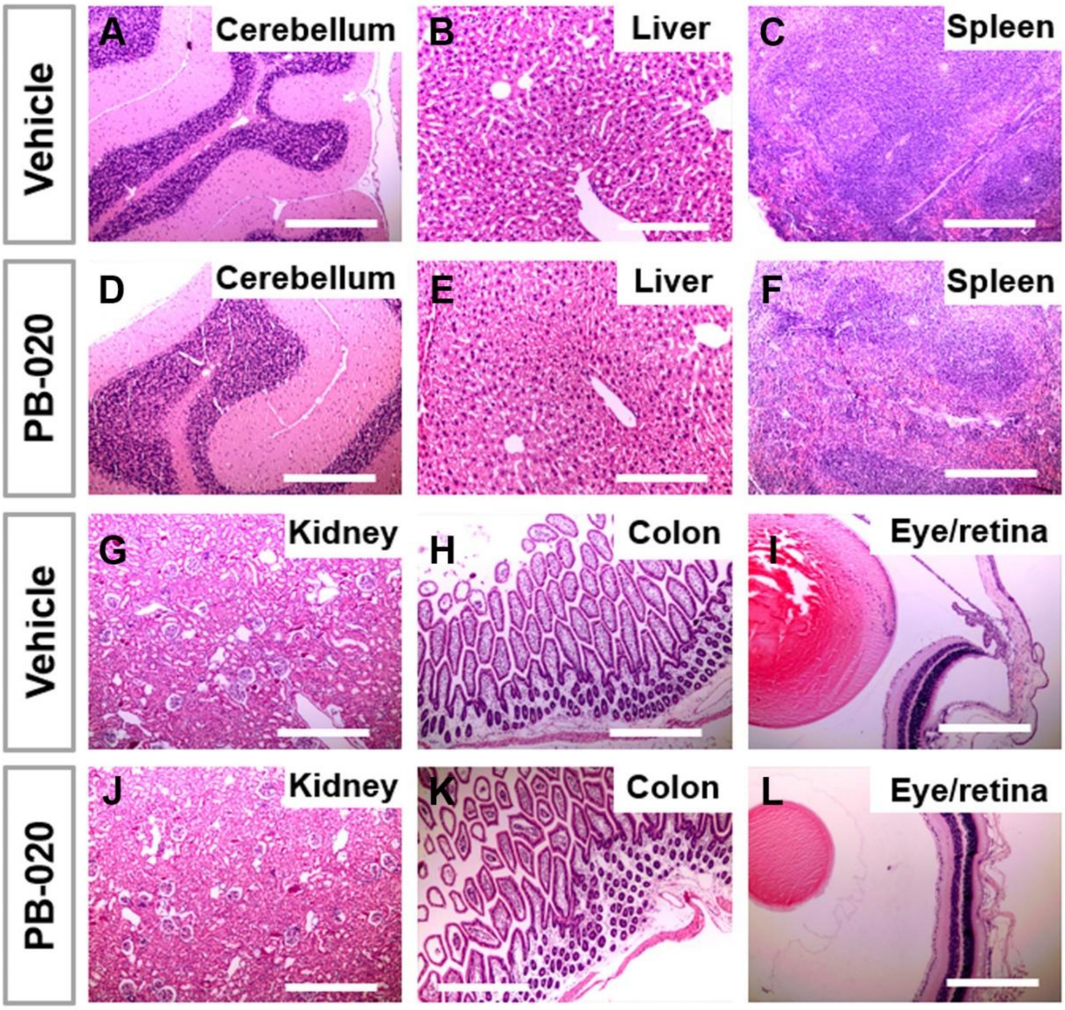

Figure S12. Oral administration of PB-020 does not trigger detectable pathological alterations in multiple key organs.

Wild-type (C57/BL6) mice were orally administrated vehicle or PB-020 (50 mg/kg/time, twice a day) for three days. Mice were dissected two hours after the last dose of drug administration. Multiple organs as indicated were analyzed through routine histological analysis, N=3 mice for each group, Scale bar: 200 μm in **(B)** and **(E)**, 400 μm in **(A)** **(C)** **(D)** **(F-L)**.

Supplementary Materials and Methods:

The genetic mouse models and their genotypes described in this study

The **CKO_NG2-Cre^{ER}** model: *p53KO, NF1flox/ p53flox, NF1flox; NG2-CreER/ WT; Rosa-tdTomato/ WT.*

The **CKO_NG2-Cre^{ER}_IGF1R (+/flox)** model: *p53KO, NF1flox / p53flox, NF1flox; NG2-CreER/ WT; Rosa-tdTomato/ WT; IGF1Rflox/WT.*

The **CKO_NG2-Cre^{ER}_IGF1R (flox/flox)** model: *p53KO, NF1flox / p53flox, NF1flox; NG2-CreER/ WT; Rosa-tdTomato/ WT; IGF1Rflox/ IGF1Rflox.*

The **WT_NG2-Cre^{ER}** model: *NG2-CreER/ WT; Rosa-tdTomato/ WT.*

The **WT_NG2-Cre^{ER}-IGF1R (flox/flox)** model: *NG2-CreER/ WT; Rosa-tdTomato/ WT; IGF1Rflox/ IGF1Rflox.*

The **CKO_Nestin-Cre^{ER}** model: *p53flox, NF1flox/ p53flox, NF1flox; Nestin-CreER/ WT; Rosa-tdTomato/ WT.*

The **CKO_Nestin-Cre^{ER}_IGF1R (flox/flox)** model: *p53flox, NF1flox / p53flox, NF1flox; Nestin-CreER/ WT; Rosa-tdTomato/ WT; IGF1Rflox/ IGF1Rflox.*

The **WT_Nestin-Cre^{ER}** model: *Nestin-CreER/ WT; Rosa-tdTomato/ WT.*

The **MADM-hGFAP-Cre** model: *TG11ML, p53KO, NF1flox/ GT11ML; hGFAP-Cre/WT.*

The **MADM-WT** model: *TG11ML/ GT11M; NG2-Cre/ WT.*

The **MADM-Mutant** model: *TG11ML, p53KO, NF1flox/ GT11ML; NG2-Cre/ WT.*

The **MADM-Mutant-IGF1R (flox/flox)** model: *TG11ML, p53KO, NF1flox / GT11ML; NG2-Cre/ WT; IGF1Rflox/ IGF1Rflox.*

The **CKO_NG2-Cre** model: *p53KO, NF1flox/ p53flox, NF1flox; NG2-Cre/ WT; Rosa-tdTomato/ WT.*

Nomenclatures for the symbols used in mouse genotypes: "," - alleles positioned on the same chromosome; ";" - alleles positioned on the unrelated chromosomes; "/" - separates the pair of

homologous chromosomes; “WT”- wild-type allele; “flox”- the floxed allele; “KO” - the null allele.

Genotyping methods

For genotyping methods, primer sequences, and PCR conditions see below:

Cre:

Upper primer 5-CACCCTGTTACGTATAGCCG-3;

Lower primer 5-GAGTCATCCTTAGCGCCGTA-3.

PCR product: KI band: 300bps.

Rosa2:

OIMR9020: 5-AAGGGAGCTGCAGTGGAGTA-3;

OIMR9021: 5-CCGAAAATCTGTGGGAAGTC-3;

OIMR9103: 5-GGCATTAAAGCAGCGTATCC-3;

OIMR9105: 5-CTGTTCCTGTACGGCATGG-3;

PCR products: Mut band: 196bp; WT band: 297bp.

MADM11_Eif (for all MADM cassettes, including TG11ML and GT11ML):

Chr11_CS1: 5-TGGAGGAGGACAAACTGGTCAC-3

Rosa4: 5-TCAATGGGCGGGGGTCGTT-3

Chr11_CS2: 5-TTCCCTTTCTGCTTCATCTTGC-3

PCR products: Knock-in (KI) band: 230bps; WT band: 350bps.

In MADM mouse model, only KI band can be detected.

P53 KO allele:

Neo tail: 5-ACCGCTATCAGGACATAGCGTTGG-3

p53 TJW5: 5-CACAGCGTGGTGGTACCTTATG-3

p53 TJW3: 5-GGTATACTCAGAGCCGGCCTG-3

PCR products: KI band: 700bps; WT band: 450bps.

NF1flox and recombination (KO) allele:

NF01: 5-ACCTCTCTAGCCTCAGGAATGA-3;

NF02: 5-CTTCAGACTGATTGTTGTACCTGA-3;

NF03: 5-TGATTCCCACTTTGTGGTTCTAAG-3 and

P2: 5-CATCTGCTGCTCTTAGAGGAACA-3

PCR products: WT band: 479bps; Flox (Neo) band: 350bp; KO band: 280bp.

P53 flox allele:

T008: 5-CACAAAAACAGGTTAAACCCAG-3;

T009: 5-AGCACATAGGAGGCAGAGAC-3;

PCR products: WT band: 288bps; Flox band: 370bp.

IGF1R-recox:

10451: 5-CTTCCCAGCTTGCTACTCTAGG-3;

10452: 5-CAGGCTTGCAATGAGACATGGG-3;

IGF1R-rec-F: 5-TGAGACGTAGCGAGATTGCTGTA-3;

PCR products: WT band: 124bps; Mut band: 220bps; Rec band: 320bps.

PCR conditions for *p53*flox, *NF1*flox, Rosa2, Eif, Cre, IGF1R-rec PCRs: one cycle at 94°C for 3minutes; 32 cycles at 94 °C for 15 seconds; 58°C for 25 seconds; 72 °C for 45 seconds;

and then followed by 72 °C for 5 minutes. For *p53*KO PCR, the annealing temperature was increased to 60 °C.

Genotyping standards for all mouse models used in this paper

To ensure the correct genotyping information, all mice used in the study were genotyped twice: the first time were performed before weaning; and the second time was done upon analysis.

The **CKO_NG2-Cre^{ER}** model: *Cre (positive), p53KO (heterozygous), p53flox (heterozygous), NF1flox (homozygous), Rosa-tdT (positive);*

The **CKO_NG2-Cre^{ER}_IGF1R (flox/flox)** model: *Cre (positive), p53KO (heterozygous), p53flox (heterozygous), NF1flox (homozygous), Rosa-tdT (positive), IGF1R-recox (homozygous);*

The **CKO_NG2-Cre^{ER}_IGF1R (+/flox)** model: *Cre (positive), p53KO (heterozygous), p53flox (heterozygous), NF1flox (homozygous), Rosa-tdT (positive), IGF1R-recox (heterozygous);*

The **WT_NG2-Cre^{ER}** model: *Cre (positive), Rosa-tdT (positive);*

The **WT_NG2-Cre^{ER}_IGF1R (flox/flox)** model: *Cre (positive), Rosa-tdT (positive), IGF1R-recox (homozygous);*

The **CKO_Nestin-Cre^{ER}** model: *Cre (positive), p53flox (homozygous), NF1flox (homozygous), Rosa-tdT (positive);*

The **CKO_Nestin-Cre^{ER}_IGF1R (flox/flox)** model: *Cre (positive), p53flox (homozygous), NF1flox (homozygous), Rosa-tdT (positive), IGF1R-recox (homozygous);*

The **WT_Nestin-Cre^{ER}** model: *Cre (positive), Rosa-tdT (positive);*

The **MADM-hGFAP-Cre** model: *Eif (homozygous), Cre (positive), p53KO (heterozygous), NF1flox (heterozygous);*

The **MADM-Mutant** mouse model: *Eif (homozygous), Cre (positive), p53KO (heterozygous), NF1flox (heterozygous);*

The **MADM-Mutant-IGF1R (flox/flox)** model: *Eif (homozygous), Cre (positive), p53KO (heterozygous), NF1flox (heterozygous), IGF1R-recox (homozygous);*

The **MADM-WT** model: *Eif (homozygous), Cre (positive), p53KO (heterozygous), NF1flox (heterozygous).*

The **CKO_NG2-Cre** model: *Cre (positive), p53KO (heterozygous), p53flox (heterozygous), NF1flox (homozygous), Rosa-tdT (positive) (used to purified neonatal precancerous OPCs); or Cre (negative), p53KO (heterozygous), p53flox (heterozygous), NF1flox (homozygous), Rosa-tdT (positive) (used to purify neonatal normal OPCs);*

Tissue Preparation and Histology

Mouse tissue preparation and histology

After anesthesia, mice were briefly perfused with cold PBS and then thoroughly perfused with 4% paraformaldehyde. Brains were isolated and post-fixed in 4% PFA before dehydrated in 30% sucrose. Fixed brain tissues were embedded into optimal cutting temperature (O.C.T.) and snap-frozen on dry ice before preserved in a -80℃ refrigerator and used for cryosection. To collect fresh tissues for Western Blot, qPCR, RNA-Seq, sc-RNA sequencing and cell culture, tissues were acutely collected from deeply anesthetized mice without perfusion. A fluorescence stereoscope was used to facilitate visualization and collection of tumor tissues whenever necessary. Fresh tissues were either snap-frozen in liquid nitrogen or directly dissociated into single cells for sc-RNA seq and/or the primary cell culture. A small piece of adjacent tumor tissue was usually collected and fixed into PFA for routine histological analyses.

Special immunofluorescence staining

BrdU staining

For BrdU staining, tissue sections, after three washes in 1x PBS (10 minutes each), were immersed in 1.5M HCl (in 1x PBS) for 30 minutes at 37°C. Following HCl treatment, tissue sections were washed three times with 1x PBS (10 minutes each) before incubated with the blocking solution (0.3% Triton X-100 in 1x PBS, 5% Normal Donkey Serum, 0.1% NaN_3) and gone through routine staining procedure as described. [7, 25]

CC1-antibody staining

To remove the nonspecific staining background signals generated by the mouse anti-CC1 monoclonal antibody (Millipore, #OP80), we used donkey-anti-mouse monovalent Fab fragment to block the tissue sections before primary antibody staining as described previously. [7, 25] After incubated in blocking solution, the slides were washed three times in 1x PBT (2 minutes each) and then incubated with Fab Donkey-anti-Mouse IgG (H+L) (1:10 diluted in 1x PBT, Jackson ImmunoResearch, #715-007-003) overnight at 4°C. The slides were then washed three times in PBT (10minutes each) before incubated with the desired primary antibody combination.

Cell culture conditions

Mouse glioma cell culture

All mouse glioma cell lines were maintained in the *mouse glioma cell culture complete media*, which contained Neurobasal media (Gibco, #21103049), L-Glutamine (Gibco, #21051-024, 1X), Penicillin/Streptomycin (HyClone, #SV30010, 1X), Sodium Pyruvate (Gibco, #11360-070, 1X), d-Biotin (Sigma, #B4639, 10 ng/ml), Gentamycin (Sigma, #46305, 50 µg/ml), Trace Element B (Cellgro, #99-175-CI, 1X), B27 minus VA (Gibco, #12587010, 5

µl/mL). The cell lines were cultured in T25 flasks that were pre-coated with Poly-D-Lysine (Sigma, #P1149).

Human GBM cell culture

All human GBM cell lines were maintained in the *human GBM cell culture complete media*, which contained Neurobasal media (Gibco, #21103-049), L-Glutamine (Gibco, #21051-024, 1X), Penicillin/Streptomycin (HyClone, #SV30010, 1X), Sodium Pyruvate (Gibco, #11360-070, 1X), d-Biotin (Sigma, #B4639, 10 ng/ml), Gentamycin (Sigma, #46305, 50 µg/ml), Trace Element B (Cellgro, #99-175-CI, 1X), Transferrin (Sigma, #T-1147, 100 µg/mL), BSA (Sigma, #A-4161, 100 µg/mL), Putrescine (Sigma, #P-5780, 16 µg/mL), Progesterone (Sigma, #P8783, 60 ng/mL), Sodium selenite (Sigma, #S5261, 40 ng/mL), Insulin (Sigma, 3I6634, 5 µl/mL), B27 minus VA (Gibco, #12587-010, 20 µl/mL), supplemented with growth factors as indicated. The concentration of growth factors was used as following: EGF (50 ng/ml) /FGF (20 ng/ml) or PDGFAA (20 ng/ml). Culture media were changed every week and the cells were passaged until cells cover 80% of the flask.

HEK293T and mouse 3T3 cell culture

Human 293T and mouse 3T3 cells were maintained in DMEM (1X), Penicillin/Streptomycin (1X) and 10% FBS (SeraPro, #S601S).

MEF cell line preparation and culture

To prepare MEFs, timed matings were set up (with the plug day as E0.5) and the embryos were collected at E13.5. As the embryos from the same litter harbored distinct genotypes, the MEFs from each embryo were prepared and used separately. For each embryo, the head, arms, legs and viscera were tossed and the remained torso was minced and digested by TrypLE

(Gibco, #12605028) and dissociated into single cells before cultured and maintained in DMEM (HyClone, #SH30022.01B), supplemented with Penicillin/Streptomycin and 10% FBS. The remained tissues were used for genotyping. As the NG2-Cre^{ER} was not expressed in the MEFs, to knock out the *Trp53* flox and the *NF1*flox allele in the mutant MEFs, we transiently transfected the cultured MEFs with a plasmid (PIGGY-Cre) that expressed Cre recombinase. The success of recombination was validated by genotyping and the strong expression of tdTomato in these cells.

Trp53/NF1 mutant and wild-type OPC culture

OPCs were enriched through immuo-panning using the anti-O4 antibody. Cells were maintained in complete media, which contained Neurobasal media (Gibco, #21103-049), L-Glutamine (Gibco, #21051-024, 1X), Penicillin/Streptomycin (HyClone, #SV30010, 1X), Sodium Pyruvate (Gibco, #11360-070, 1X), Gentamycin (Sigma, #46305, 50 µg/mL), Trace Elements B (Cellgro, #99-175-CI, 1X), d-Biotin (Sigma, #B4639, 10 ng/mL), SATO 10 µl/mL, Transferrin (Sigma, #T-1147, 100 µg/mL), BSA (Sigma, #A-4161, 100 µg/mL), Putrescine (Sigma, #P-5780, 16 µg/mL), Progesterone (Sigma, #P8783, 60 ng/mL), Sodium selenite (Sigma, #S5261, 40 ng/mL), Insulin (Sigma, 3I6634, 5 µl/mL), B27minusVA (Gibco, #12587-010, 20 µl/mL), supplemented with growth factors as indicated. The concentration of growth factors was used as following: FGF (20 ng/ml) and PDGFAA (20 ng/ml). The plates were pre-coated by PDL.

Real-Time qPCR

Total RNA was isolated by the Trizol Reagent and reversely transcribed using the oligo (dT) primer. qRT-PCR was performed on the CFX96 Touch™ Real-Time PCR Detection System using HieffTM qPCR SYBR® Green Master Mix. β-actin (ActB) and/or glyceraldehyde-3-

phosphate dehydrogenase (GAPDH) were used as the internal control to normalize the expression of the interested genes. See **Table S4 (Supporting Information)** for primer information.

Chemical synthesis of analogs of Picropodophyllotoxin (PPP, CAS no. 477-47-4)

PB-001: rel-(5R,5aS,8aR,9R)-2,2-difluoro-9-hydroxy-5-(3,4,5-trimethoxyphenyl)-5,8,8a,9-tetra hydrofuro[3',4':6,7]naphtho[2,3-d][1,3]dioxol-6(5aH)-one (**compound 1**)

Step 1: Synthesis of intermediate 4-vinyldihydrofuran-2(3H)-one (**1.1**)

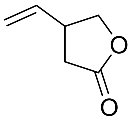

1.1

To a mixture of 2-butene-1,4-diol (206.4 g, 2.34 mol, 1.0 eq) and triethyl orthoacetate (546.7 g, 3.4 mol, 1.4 eq) was added catalytic hydroquinone (54.00 g, 0.49 mol, 0.2 eq), and the resultant mixture was heated to 120℃. Ethanol formed *in situ* was distilled out continuously until no more ethanol was produced. The temperature was increased to 150℃, and the reaction mixture was stirred for additional 48 hours. Intermediate **1.1** was collected by vacuum distillation (70-75℃, 2-3 mmHg) as colorless oil (170.0 g, 65% yield). ^{1}HNMR (400 MHz, $CDCl_3$) δ(ppm) 2.36 (dd, *J* = 17.4 Hz, 8.7 Hz, 1H), 2.69 (dd, *J* = 17.7 Hz, 8.4 Hz, 1H), 3.19-3.29 (m, 1H), 4.01-4.14 (m, 1H), 4.43-4.47 (m, 1H), 5.17-5.23 (m, 2H), 5.75-5.84 (m, 1H).

Step 2: Synthesis of intermediate rel-(3S,4R)-3-(hydroxy(3,4,5-trimethoxyphenyl)methyl)-4-vinyl dihydrofuran-2(3H)-one (**1.2**)

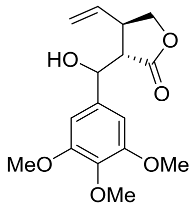

1.2

To a stirred solution of intermediate **1.1** (170.0 g, 1.52 mol, 1.0 eq) in THF (1500 ml) was added dropwise LDA (2.0 M, 834 ml, 1.67 mol, 1.1 eq) at -78℃ under N_2 atmosphere, and the reaction mixture was stirred for additional 30 min. A solution of 3,4,5-trimethoxybenzaldehyde (327.6 g, 1.67 mol, 1.1 eq) in THF (1500 ml) was added dropwise at the same condition, and the obtained mixture was stirred for additional 3 hours before gradually warmed to ambient temperature overnight. The reaction mixture was cooled to -78℃, and was quenched with sat'd NH_4Cl. The resultant mixture was extracted with EtOAc, washed with brine, dried over $MgSO_4$, and was concentrated to dryness. The obtained residue was purified by column chromatography (200-300 silica gel, PE/EtOAc=5/1-1/1) to afford intermediate **1.2** (190.1 g, 41% yield) as light yellow solid. ^{1}HNMR (400 MHz, $CDCl_3$) δ(ppm) 2.75-2.80 (m, 1H), 2.91-2.96 (m, 0.5H), 3.26-3.31 (m, 0.5H), 3.84 (s, 3H), 3.89 (s, 6H), 3.92 (t, 1H), 4.30-4.40 (m, 1H), 4.86-4.93 (m, 2H), 6.59 (s, 1H), 6.61 (s, 1H).

Step 3: Synthesis of intermediate rel-(3R,4R)-3-((R)-(2,2-difluoro-6-hydroxybenzo[d][1,3]dioxol-5 -yl)(3,4,5-trimethoxyphenyl)methyl)-4-vinyldihydrofuran-2(3H)-one (**1.3**)

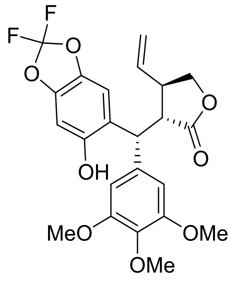

1.3

To a stirred solution of intermediate **1.3** (106.7 g, 0.41 mol, 1.0 eq) and 2,2-difluorobenzo[d][1,3] dioxol-5-ol (107.6 g, 0.61 mol, 1.5 eq) in DCM (2000 ml) was added $FeCl_3$ (34.06 g, 0.20 mol, 0.5 eq), and the reaction mixture was heated to 40℃ for 2-3 hours. The reaction mixture was quenched with sat'd $NaHCO_3$, and aqueous phase was extracted with additional DCM. The combined organic phase was washed with brine, dried over $MgSO_4$, and was concentrated to dryness. The obtained residue was purified by column chromatography (200-300 silica gel, PE/EtOAc=5/1-1/1) to afford intermediate **1.3** (153.4 g, 45% yield) as off-white solid. ^{1}HNMR (400 MHz, $CDCl_3$) δ(ppm) 3.02-3.06 (m, 1 H), 3.19-3.21 (m, 1 H), 3.83 (s, 6H), 3.88 (s, 3H), 4.04-4.07 (m, 1H), 4.25-4.29 (m, 1H), 4.73-4.75 (d, *J* = 4.8 Hz, 1H), 5.14-5.22 (m, 2H), 5.55 (brs, 1H), 5.80-5.84 (m, 1H), 6.51-6.59 (m, 2H), 6.82-6.96 (m, 1H), 7.29 (s, 1H).

Step 4: Synthesis of intermediate rel-2,2-difluoro-6-((R)-((3R,4R)-2-oxo-4-vinyltetrahydrofuran-3 -yl)(3,4,5-trimethoxyphenyl)methyl)benzo[d][1,3]dioxol-5-yl trifluoromethanesulfonate (**1.4**)

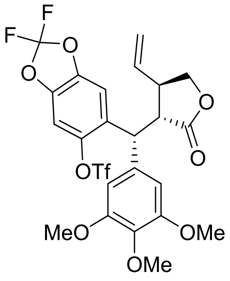

1.4

To a stirred solution of intermediate **1.3** (120.0 g, 0.26 mol, 1.0 eq) in DCM (1500 ml) was added Et_3N (52.62 g, 0.52 mol, 2.0 eq) followed by dropwise addition of Tf_2O (110.0 g, 0.39 mol, 1.5 eq) at 10℃, and the resultant mixture was stirred for additional 30 min at the same temperature. The reaction mixture was quenched with sat'd $NaHCO_3$, and aqueous phase was extracted with additional DCM. The combined organic phase was washed with 2 N HCl,

washed with brine, dried over $MgSO_4$, and was concentrated to dryness. The obtained residue was purified by column chromatography (200-300 silica gel, PE/EtOAc=6/1-3/1) to afford intermediate **1.4** (120.0 g, 61% yield) as white solid. ^{1}HNMR (400 MHz, $CDCl_3$) δ(ppm) 2.97-3.06 (m, 1H), 3.13-3.17 (m, 1H), 3.84 (s, 6H), 3.85 (s, 3H), 4.03-4.08 (m, 1H), 4.37-4.41 (m, 1H), 4.64-4.66 (d, *J* = 8.3Hz, 1H), 5.08-5.20 (dt, 2H), 5.71-5.80 (m, 1H), 6.58 (s, 2H), 7.04 (s, 1H), 7.23 (s, 1H).

Step 5: Synthesis of intermediate rel-(5R,5aR,8aS)-2,2-difluoro-9-methylene-5-(3,4,5-trimethoxy phenyl)-5,8,8a,9-tetrahydrofuro[3',4':6,7]naphtho[2,3-d][1,3]dioxol-6(5aH)-one (**1.5**)

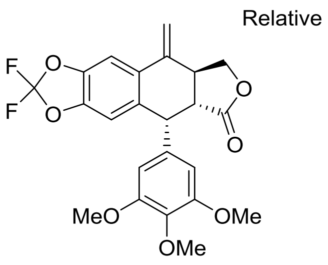

1.5

To a stirred solution of intermediate **1.4** (120.0 g, 0.20 mol, 1.0 eq) in MeCN (1500 ml) was added PPh_3 (15.83 g, 60.0 mmol, 0.3 eq), K_2CO_3 (82.93 g, 0.60 mol, 3.0 eq) and $Pd(OAc)_2$ (4.49 g, 20.0 mmol, 10 mol%), and the resultant mixture was heated to 80℃ for 20 hours. The reaction mixture was filtered, and filter cake was washed with DCM. The combined organic phase was concentrated to dryness, and the obtained residue was purified by column chromatography (200-300 silica gel, PE/EtOAc=5/1-1/1) to afford intermediate **1.5** (76.13 g, 61% yield) as white solid. ^{1}HNMR (400 MHz, $CDCl_3$) δ(ppm) 3.31-3.34 (dd, 1H), 3.65-3.68 (t, 1H), 3.76 (s, 6H), 3.84 (s, 3H), 4.21-4.24 (dd, 1H), 4.57-4.59 (m, 2H), 5.21-5.22 (d, 1), 5.57 (s, 1H), 6.31 (s, 2H), 6.95 (s, 1H), 7.27 (s, 1H).

Step 6: Synthesis of intermediate rel-(5aR,8aR,9R)-2,2-difluoro-9-(3,4,5-trimethoxyphenyl)-5a,6,8a,9-tetrahydrofuro[3',4':6,7]naphtho[2,3-d][1,3]dioxole-5,8-dione (**1.6**)

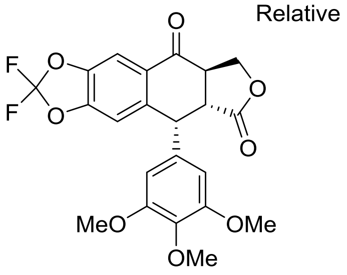

1.6

To a stirred solution of intermediate **1.5** (79.04 g, 0.18 mol, 1.0 eq) and N-methylmorpholin-N- oxide (168.7 g, 0.72 mol, 3.0 eq) in DCM (1200 ml) was added OsO_4 (4.00 g, 15.7 mmol, 8 mol%), and the reaction mixture was stirred at ambient temperature for 12 hours. Solid $NaIO_4$ (77.00 g, 0.36 mol, 2.0 eq) was added in portions, and the obtained mixture was stirred for additional 1 hour. The reaction mixture was quenched with sat'd $Na_2S_2O_3$ (300 ml) at ice-water bath temperature. Aqueous phase was extracted with additional DCM. The combined organic phase was washed with brine, dried over $MgSO_4$, and was concentrated to dryness. The obtained residue was purified by column chromatography (200-300 silica gel, PE/EtOAc=5/1-1/1) to afford intermediate **1.6** (48.80 g, 60% yield) as white solid. ^{1}HNMR (400 MHz, $CDCl_3$) δ (ppm) 3.37-3.42 (m, 2H), 3.78 (s, 6H), 3.82 (s, 3H), 4.38-4.42 (m, 1H), 4.79-4.80 (d, J = 2.8 Hz, 2H), 6.22 (s, 2H), 7.02 (s, 1H), 7.80 (s, 1H).

Step 7: Synthesis of rel-(5R,5aS,8aR,9R)-2,2-difluoro-9-hydroxy-5-(3,4,5-trimethoxyphenyl)-5,8,8a,9-tetrahydrofuro[3',4':6,7]naphtho[2,3-d][1,3]dioxol-6(5aH)-one (**compound 1**)

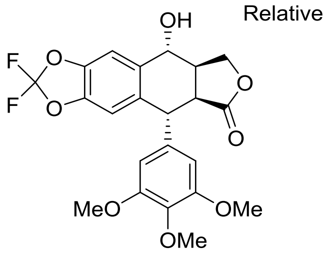

To a stirred solution of intermediate **1.6** (43.30 g, 98.9 mmol, 1.0 eq) in Et_2O (1500 ml) was added dropwise a solution of LiAl $(O^tBu)_3$ (200 ml, 197 mmol, 2.0 eq) at -78℃ under N_2 atmosphere, and the resultant mixture was gradually warmed to ambient temperature overnight. The reaction was quenched with 2 N HCl at ice-water bath temperature, and aqueous phase was extracted with additional DCM. The combined organic phase was washed with brine, dried over $MgSO_4$, and was concentrated to dryness. The obtained residue was recrystallized from EtOAc to afford compound **1** (33.10 g, 74% yield) as white solid. ^{1}HNMR (400 MHz, $CDCl_3$) δ(ppm) 7.38 (s, 1H), 6.53 (s, 1H), 6.47 (s, 2H), 4.67 (d, *J* = 0.9 Hz, 1H), 4.55-4.43 (m, 2H), 4.03 (d, *J* = 6.4 Hz, 1H), 3.89 (s, 3H), 3.85 (s, 6H), 3.26 (q, 1H), 2.96 (d, *J* = 6.9 Hz, 1H), 2.66 (m, 1H).

PB-002: rel-(5R,5aR,8aS,9R)-2,2-difluoro-8-oxo-9-(3,4,5-trimethoxyphenyl)-5,5a,6,8,8a,9-hexa hydrofuro[3',4':6,7]naphtho[2,3-d][1,3]dioxol-5-yl acetate (**compound 2**)

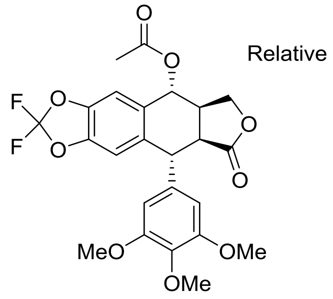

To a stirred solution of compound **1** (1.15 g, 2.55 mmol, 1.0 eq) in DCM (30 ml) was added Et_3N (770 mg, 7.6 mmol, 3.0 eq), DMAP (310 mg, 2.55 mmol, 1.0 eq) followed by AcCl (400 mg, 5.1 mmol, 2.0 eq) at ice-water bath temperature, and the resultant mixture was stirred at ambient temperature for 12 hours. The reaction mixture was quenched with sat'd NH_4Cl, and aqueous phase was extracted with additional DCM. The combined organic phase was washed with brine, dried over $MgSO_4$, and was concentrated to dryness. The obtained residue was recrystallized from PE/EtOAc to afford compound **2** (810 mg, 65% yield) as white solid. ^{1}HNMR (400 MHz, $CDCl_3$) δ (ppm) 7.02 (s, 1H), 6.76 (s, 1H), 6.41 (s, 2H), 5.81

(d, J= 6.3 Hz, 1H), 4.48-4.34 (m, 3H), 3.87 (s, 3H), 3.84 (s, 6H), 3.32 (d, 1H), 3.01 (m, 1H), 2.12 (s, 3H).

PB-006: rel-(5R,5aS,8aR,9S)-2,2,9-trifluoro-5-(3,4,5-trimethoxyphenyl)-5,8,8a,9-tetrahydrofuro[3',4':6,7]naphtho[2,3-d][1,3]dioxol-6(5aH)-one (**compound 3**)

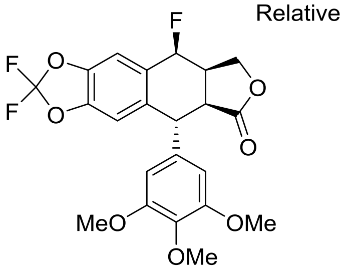

To a stirred solution of compound **1** (700 mg, 1.60 mmol, 1.0 eq) in DCM (20 ml) was added dropwise DAST (520 mg, 3.20 mmol, 2.0 eq), and the resultant mixture was stirred at ambient temperature for 12 hours. The reaction mixture was quenched with sat'd $NaHCO_3$, and was stirred for additional 30 min. organic phase was separated, and aqueous phase was extracted with additional DCM. The combined organic phase was washed with brine, dried over $MgSO_4$, and was concentrated to dryness. The obtained residue was purified by column chromatography (200-300 silica gel, DCM/MeOH=100/1-50/1) to afford compound **3** (130 mg, 18% yield) as white solid. ^{1}HNMR (400 MHz, $CDCl_3$) δ(ppm) 7.27 (s, 1H), 6.66 (s, 1H), 6.44 (s, 2H), 5.49-5.34 (d, *J*=50.8 Hz, 1H), 4.60-4.47 (m, 2H), 4.20 (d, *J*= 5.0 Hz, 1H), 3.88 (s, 3H), 3.84 (s, 6H), 3.28 (m, 1H), 3.01 (m, 1H).

PB-007: rel-(5R,5aS,8aR,9R)-2,2-difluoro-9-methoxy-5-(3,4,5-trimethoxyphenyl)-5,8,8a,9-tetrahydrofuro[3',4':6,7]naphtho[2,3-d][1,3]dioxol-6(5aH)-one (**compound 4**)

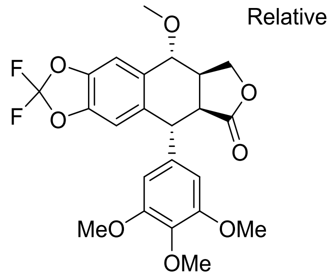

To a stirred solution of compound **1** (600 mg, 1.33 mmol, 1.0 eq) in DCM (15 ml) was added trimethyloxonium tetrafluoroborate (246 mg, 1.66 mmol, 1.2 eq), and the resultant mixture was stirred at ambient temperature for 20 hours. The reaction was quenched with sat'd $NaHCO_3$, and was stirred for additional 30 min. organic phase was separated, and aqueous phase was extracted with additional DCM. The combined organic phase was washed with brine, dried over $MgSO_4$, and was concentrated to dryness. The obtained residue was purified by column chromatography (200-300 silica gel, DCM/MeOH=100/1-50/1) to afford compound 4 (65 mg, 11% yield) as white solid. ^{1}HNMR (400 MHz, $CDCl_3$) δ (ppm) 7.16 (s, 1H), 6.74 (s, 1H), 6.41 (s, 2H), 4.45-4.36 (m, 3H), 4.34 (d, J = 4.3 Hz, 1H), 3.88 (s, 3H), 3.83 (s, 6H), 3.42 (d, 1H), 3.31 (s, 3H), 3.14 (m, 1H).

PB-009: rel-(5R,5aS,8aR,9R)-2,2-difluoro-9-hydroxy-5-(3,4,5-trimethoxyphenyl)-5,8,8a,9-tetrahydrofuro[3',4':6,7]naphtho[2,3-d][1,3]dioxol-6(5aH)-one-9-d (**compound 5**)

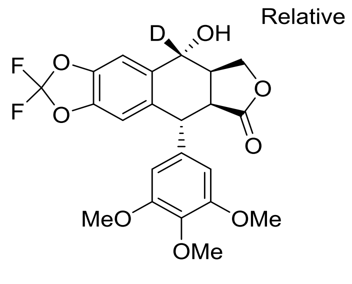

To a stirred solution of intermediate **1.6** (400 mg, 0.89 mmol, 1.0 eq) in methanol (10 ml) was added $NaBD_4$ (37.5 mg, 0.89 mmol, 1.0 eq) in portions, and the resultant mixture was stirred for 4 hours. The reaction was quenched with water, and was concentrated to dryness. The obtained residue was diluted with water, and was extracted with EtOAc. The combined

organic phase was washed with brine, dried over $MgSO_4$, and was concentrated to dryness. The obtained residue was recrystallized from PE/EtOAc to afford compound **5** (140 mg, 35% yield) as white solid. ^{1}HNMR (400 MHz, $CDCl_3$) δ(ppm) 7.38 (s, 1H), 6.57 (s, 1H), 6.47 (s, 2H), 4.66 (d, J= 9.8 Hz, 1H), 4.47 (m, 1H), 4.06 (d, J= 6.2 Hz, 1H), 3.89 (s, 3H), 3.87 (s, 6H), 3.27 (d, 1H), 2.68 (m, 1H), 2.44 (s, 1H).

PB-004: (5R,5aS,8aR,9R)-2,2-difluoro-9-hydroxy-5-(3,4,5-trimethoxyphenyl)-5,8,8a,9-tetrahydrofuro[3',4':6,7]naphtho[2,3-d][1,3]dioxol-6(5aH)-one (**compound 6**)

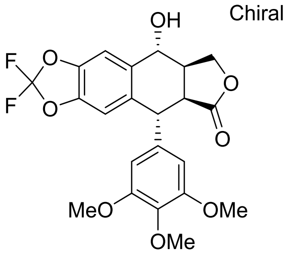

Compound PB-004 was prepared by chiral separation of compound **1** on chiral column chromatography. ^{1}HNMR (400 MHz, $CDCl_3$) δ(ppm) 7.38 (s, 1H), 6.53 (s, 1H), 6.47 (s, 2H), 4.67 (d, J = 0.9 Hz, 1H), 4.55-4.43 (m, 2H), 4.03 (d, J = 6.4 Hz, 1H), 3.89 (s, 3H), 3.85 (s, 6H), 3.26 (q, 1H), 2.96 (d, J = 6.9 Hz, 1H), 2.66 (m, 1H); *e.e.*= 98.56%; [α]= 11.94^o (MeOH, c=0.88 g/100 ml).

Preparative separation method

Instrument: waters SFC200

Column: Daicel Chiralcel AD, 250×50mm I.D., 10µm

Mobile phase: A for CO2 and B for isopropanol

Gradient: B 20%

Flow rate: 150mL /min

Back pressure: 100bar

Column temperature: 38℃

Wavelength: 220nm

Cycle time: 6.5min

Sample preparation: Compound was dissolved in ~600 ml MEOH

Injection: 3ml per injection

PB-016: rel-(5R,5aS,8aR,9R)-2,2-difluoro-9-hydroxy-5-(3,4,5-trimethoxyphenyl)-5,8,8a,9-tetra hydrofuro[3',4':6,7]naphtho[2,3-d][1,3]dioxol-6(5aH)-one-9-d (**compound 7**)

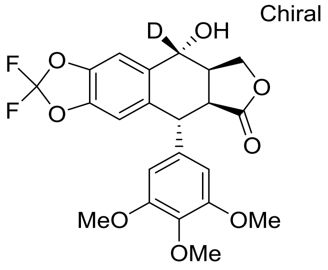

Compound PB-016 was prepared by chiral separation of compound **5** on chiral column chromatography. ^{1}HNMR (400 MHz, $CDCl_3$) δ(ppm) 7.38 (s, 1H), 6.57 (s, 1H), 6.47 (s, 2H), 4.66 (d, J= 9.8 Hz, 1H), 4.47 (m, 1H), 4.06 (d, J= 6.2 Hz, 1H), 3.89 (s, 3H), 3.87 (s, 6H), 3.27 (d, 1H), 2.68 (m, 1H), 2.44 (s, 1H).

Preparative separation method

Instrument: waters SFC200

Column: Daicel Chiralcel AD, 250×50mm I.D., 10µm

Mobile phase: A for CO2 and B for isopropanol

Gradient: B 20%

Flow rate: 150mL /min

Back pressure: 100bar

Column temperature: 38℃

Wavelength: 220nm

Cycle time: 6.5min

Sample preparation: Compound was dissolved in ~600 ml MEOH

Injection: 3ml per injection

PB-017: rel-(5R,5aS,8aR,9R)-2,2-methyl-9-hydroxy-5-(3,4,5-trimethoxyphenyl)-5,8,8a,9-tetra hydrofuro[3',4':6,7]naphtho[2,3-d][1,3]dioxol-6(5aH)-one (**compound 8**)

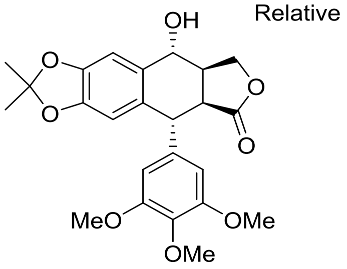

Compound PB-017 was synthesized in similar method with compound **1**, using 2,2-methylbenzo[d][1,3] dioxol-5-ol as starting material. ^{1}HNMR (400 MHz, $CDCl_3$) δ(ppm) 6.95 (s, 1H), 6.48 (s, 2H), 6.30 (s, 1H), 4.43-4.53 (m, 3H), 4.12 (d, J=5.2 Hz, 1H), 3.87 (s, 3H), 3.84 (s, 6H), 3.25 (dd, J=5.2 Hz, 9.3 Hz, 1H), 2.74-2.80 (m, 1H), 2.18 (brs, 1H), 1.68 (s, 3H), 1.65 (s, 3H).

PB-018: rel-(5R,5aS,8aR,9R)-2,2-difluoro-9-hydroxy-5-(3,4,5-trimethoxyphenyl)-5,8,8a,9-tetra hydrofuro[3',4':6,7]naphtho[2,3-d][1,3]dioxol-6(5aH)-one-5,9-d2 (**compound 9**)

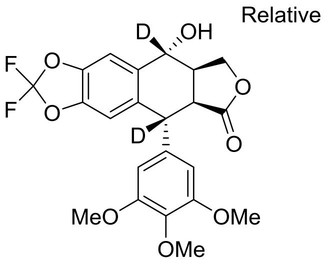

Compound PB-018 was synthesized in similar method with compound **5**, using 3,4,5-Trimethoxybenzaldehyde-formyl-d as starting material. ^{1}HNMR (400 MHz, $CDCl_3$) δ(ppm) 7.38 (s, 1H), 6.56 (s, 1H), 6.47 (s, 2H), 4.65 (d, J=9.8 Hz, 1H), 4.47 (dd, J=6.1 Hz, 9.7 Hz, 1H), 3.90 (s, 3H), 3.86 (s, 6H), 3.26 (d, J=9.4 Hz, 1H), 2.66-2.70 (m, 1H), 2.52 (brs, 1H).

PB-019: rel-(5R,5aS,8aR,9R)-2,2-difluoro-9-hydroxy-5-(3,4,5-tris(methoxy-d3)phenyl)-5,8,8a, 9-tetrahydrofuro[3',4':6,7]naphtho[2,3-d][1,3]dioxol-6(5aH)-one-9-d (**compound 10**)

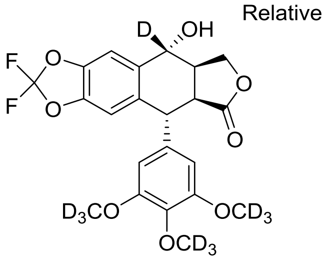

Compound PB-019 was synthesized in similar method with compound **5**, using 3,4,5-Tris(trideuteriomethoxy)benzaldehyde as starting material. ^{1}HNMR (400 MHz, $CDCl_3$) δ(ppm) 7.38 (s, 1H), 6.55 (s, 1H), 6.46 (s, 2H), 4.64 (dd, J=1.2 Hz, 9.8 Hz, 1H), 4.45 (dd, J=6.0 Hz, 9.8 Hz, 1H), 4.04 (d, J=6.3 Hz, 1H), 3.26 (dd, J=6.4 Hz, 9.3 Hz, 1H), 2.78 (s, 1H), 2.66 (dd, J=6.0 Hz, 8.5 Hz, 1H).

PB-020: (5R,5aS,8aR,9R)-2,2-difluoro-9-hydroxy-5-(3,4,5-tris(methoxy-d3)phenyl)-5,8,8a,9-tetrahydrofuro[3',4':6,7]naphtho[2,3-d][1,3]dioxol-6(5aH)-one-9-d (**compound 11**)

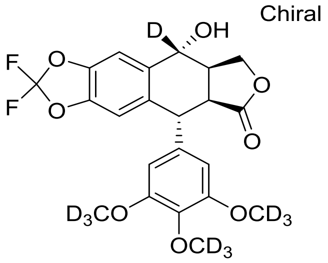

Compound PB-020 was prepared by chiral separation of compound **10** on chiral column chromatography. ^{1}HNMR (400 MHz, $CDCl_3$) δ(ppm) 7.38 (s, 1H), 6.55 (s, 1H), 6.46 (s, 2H),

4.64 (dd, J=1.2 Hz, 9.8 Hz, 1H), 4.45 (dd, J=6.0 Hz, 9.8 Hz, 1H), 4.04 (d, J=6.3 Hz, 1H), 3.26 (dd, J=6.4 Hz, 9.3 Hz, 1H), 2.78 (s, 1H), 2.66 (dd, J=6.0 Hz, 8.5 Hz, 1H).

Preparative separation method

Instrument: waters SFC200

Column: Daicel Chiralcel AD, 250×50mm I.D., 10µm

Mobile phase: A for CO2 and B for isopropanol

Gradient: B 20%

Flow rate: 150mL /min

Back pressure: 100bar

Column temperature: 38℃

Wavelength: 220nm

Cycle time: 6.5min

Sample preparation: Compound was dissolved in ~600 ml MEOH

Injection: 3ml per injection

Representative compounds with structures, ^{1}HNMR, MS, cellular activity data

Compound	Structure	^{1}HNMR (400MHz, $CDCl_3$) δ(ppm)	MS $[M+H]^+$	U87 IC_{50} (nM)
PB-001 (compound 1)	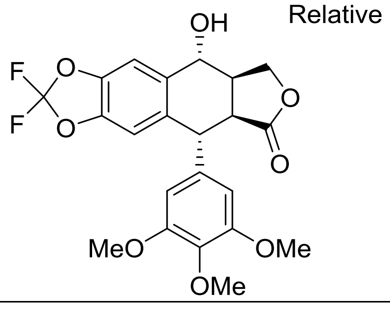	7.38 (s,1H), 6.53(s,1H), 6.47(s,2H), 4.67(d, J= 0.9 Hz, 1H), 4.55-4.43 (m,2H), 4.03(d, J= 6.4 Hz, 1H), 3.89 (s, 3H), 3.85 (s, 6H), 3.26 (q, 1H), 2.96 (d, J= 6.9 Hz, 1H), 2.66 (m, 1H)	451	2726

PB-002 (compound 2)	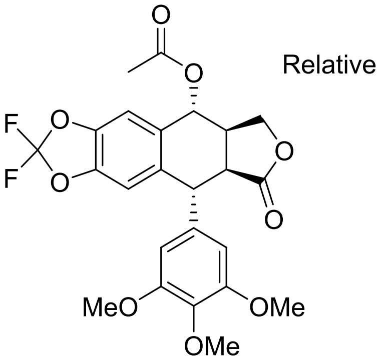	7.02 (s, 1H), 6.76 (s, 1H), 6.41 (s, 2H), 5.81 (d, J= 6.3 Hz, 1H), 4.48-4.34 (m, 3H), 3.87 (s, 3H), 3.84 (s, 6H), 3.32 (d, 1H), 3.01 (m, 1H), 2.12 (s, 3H)	493	6346
PB-006 (compound 3)	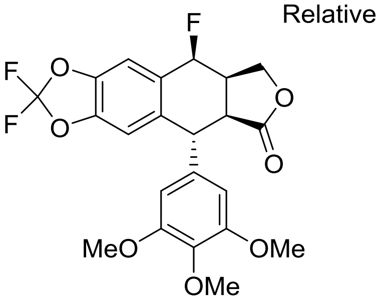	7.27 (s, 1H), 6.66 (s, 1H), 6.44 (s, 2H), 5.49-5.34 (d, J=50.8 Hz, 1H), 4.60-4.47 (m, 2H), 4.20 (d, J= 5.0 Hz, 1H), 3.88 (s, 3H), 3.84 (s, 6H), 3.28 (m, 1H), 3.01 (m, 1H)	453	7506
PB-007 (compound 4)	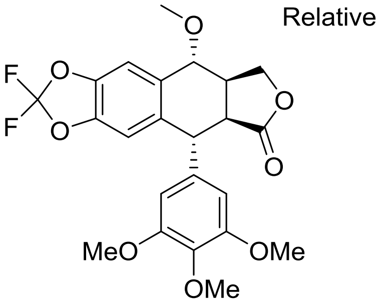	7.16 (s, 1H), 6.74 (s, 1H), 6.41 (s, 2H), 4.45-4.36 (m, 3H), 4.34 (d, J= 4.3 Hz, 1H), 3.88 (s, 3H), 3.83 (s, 6H), 3.42 (d, 1H), 3.31 (s, 3H), 3.14 (m, 1H)	465	4346
PB-009 (compound 5)	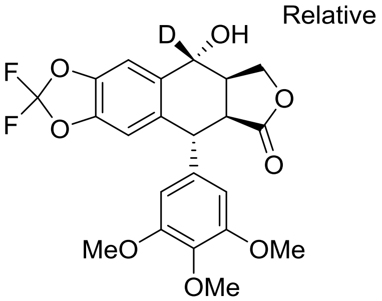	7.38 (s, 1H), 6.57 (s, 1H), 6.47 (s, 2H), 4.66 (d, J= 9.8 Hz, 1H), 4.47 (m, 1H), 4.06 (d, J= 6.2 Hz, 1H), 3.89 (s, 3H), 3.87 (s, 6H), 3.27 (d, 1H), 2.68 (m, 1H), 2.44 (s, 1H)	452	2499
PB-004 (compound 6)	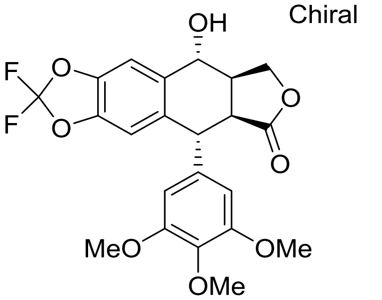	7.38 (s,1H), 6.53(s,1H), 6.47(s,2H), 4.67(d, J= 0.9 Hz, 1H), 4.55-4.43 (m,2H), 4.03(d, J= 6.4 Hz, 1H), 3.89 (s, 3H), 3.85 (s, 6H), 3.26 (q, 1H), 2.96 (d, J= 6.9 Hz, 1H), 2.66 (m, 1H)	451	902

PB-016 (compound 7)	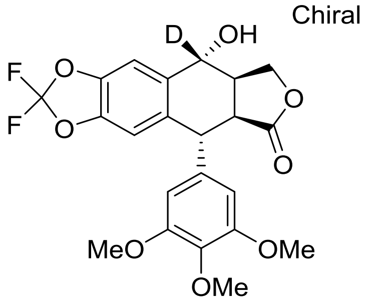	7.38 (s, 1H), 6.57 (s, 1H), 6.47 (s, 2H), 4.66 (d, J= 9.8 Hz, 1H), 4.47 (m, 1H), 4.06 (d, J= 6.2 Hz, 1H), 3.89 (s, 3H), 3.87 (s, 6H), 3.27 (d, 1H), 2.68 (m, 1H), 2.44 (s, 1H)	452	1340
PB-017 (compound 8)	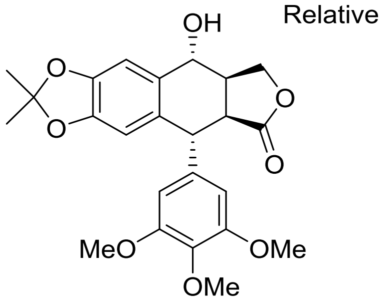	6.95 (s, 1H), 6.48 (s, 2H), 6.30 (s, 1H), 4.43-4.53 (m, 3H), 4.12 (d, J=5.2 Hz, 1H), 3.87 (s, 3H), 3.84 (s, 6H), 3.25 (dd, J=5.2 Hz, 9.3 Hz, 1H), 2.74-2.80 (m, 1H), 2.18 (brs, 1H), 1.68 (s, 3H), 1.65 (s, 3H)	443	7486
PB-018 (compound 9)	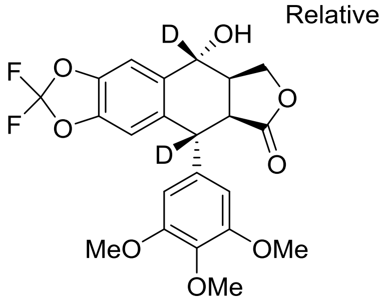	7.38 (s, 1H), 6.56 (s, 1H), 6.47 (s, 2H), 4.65 (d, J=9.8 Hz, 1H), 4.47 (dd, J=6.1 Hz, 9.7 Hz, 1H), 3.90 (s, 3H), 3.86 (s, 6H), 3.26 (d, J=9.4 Hz, 1H), 2.66-2.70 (m, 1H), 2.52 (brs, 1H)	453	1994
PB-019 (compound 10)	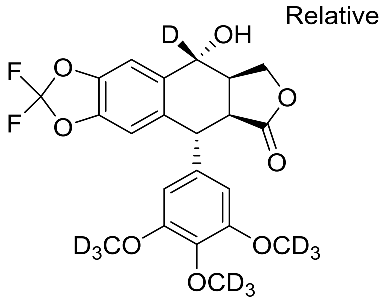	7.38 (s, 1H), 6.55 (s, 1H), 6.46 (s, 2H), 4.64 (dd, J=1.2 Hz, 9.8 Hz, 1H), 4.45 (dd, J=6.0 Hz, 9.8 Hz, 1H), 4.04 (d, J=6.3 Hz, 1H), 3.26 (dd, J=6.4 Hz, 9.3 Hz, 1H), 2.78 (s, 1H), 2.66 (dd, J=6.0 Hz, 8.5 Hz, 1H)	461	1539
PB-020 (compound 11)	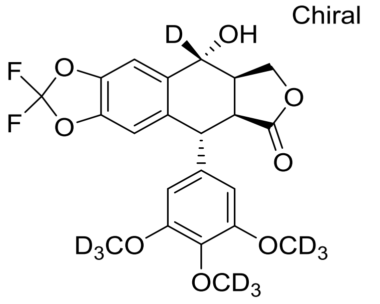	7.38 (s, 1H), 6.55 (s, 1H), 6.46 (s, 2H), 4.64 (dd, J=1.2 Hz, 9.8 Hz, 1H), 4.45 (dd, J=6.0 Hz, 9.8 Hz, 1H), 4.04 (d, J=6.3 Hz, 1H), 3.26 (dd, J=6.4 Hz, 9.3 Hz, 1H), 2.78 (s, 1H), 2.66 (dd, J=6.0 Hz, 8.5 Hz, 1H)	461	1070

Quantification schemes

Below we describe the schemes to quantify all the *in vivo* models in the paper. All the raw data can be found in **Table S7 (Supporting Information)**.

Quantification of tumor Spheres

Tumor cells were incubated with growth factors as indicated for 4-7 days. Human GBM spheres were cultured for 14 days, and 50% of the culture media was replaced for every 7 days. Images for each well were taken with Fluorescence Inverted Microscope System (OLYMPUS CKX53) in 10x magnifier. 4 images (one for each well) were analyzed for each condition. The size and/or the number of spheres were quantified and measured by Image J. We defined tumor spheres as the cluster with more than 3 cells. The diagraph and statistical analysis were done with Graphpad Prism 5.

Quantification for the NG2-Cre^{ER} mouse models in Figure 3

The cell proliferation rates were quantified by the percentage of the cells that incorporated BrdU. The mouse brains were sectioned coronally in 20 μm thickness and stained with BrdU (1:500), DsRed (1:100) and Olig2 (1:500). We used DAPI to determine the anatomical regions and collected images from the olfactory bulb (OB) and distinct cortical regions. We randomly collected images from 10 brain sections in the OB and 6 sections in the cerebral cortex. For each brain section, 4 regions were collected in the OB and 6 regions were collected in the cerebral cortex. Each image was taken by a 20x objective with 1x digital zoom by 2 μm optical sectioning (scanning depth 6 μm). We quantified the BrdU$^+$, DsRed$^+$, Olig2$^+$ co-stained cells and the DsRed$^+$, Olig2$^+$ co-stained cells. These counts were used to calculate the proliferation rate of labeled OPCs (the percentage of BrdU labeling among all mutant OPCs) in the brains of distinct genotypes (see **Figure 3C-3D**). These values were averaged from N=3 brains and presented as mean ± SEM.

To calculation the differentiation rates, we sectioned the brain coronally in 20 μm thickness and co-stained with PDGFRα (1:250), and APC-CC1 (1:50). We used DAPI to define the corpus callosum. For each brain, we collected images from the corpus callosum of 6 non-adjacent sections (30 images for each brain). Each image was taken by a 20x objective with 1x digital zoom by 2 μm optical sectioning (scanning depth 6μm).We counted all tdT^+, $PDGFR\alpha^+$, $CC1^-$ (OPCs), tdT^+,$PDGFR\alpha^+$,$CC1^+$ (newly formed oligodendrocytes) and tdT^+, $PDGFR\alpha^-$, $CC1^+$ (mature oligodendrocytes) cells (see **Figure 3G**).The relative percentage of these three sub-populations were then calculated. The number was averaged from N=3 brains and presented as mean ± SEM.

Quantification for the G/R ratios and the density of OPC-lineage cells in the MADM mouse models presented in Figure 4

To quantify MADM labeled OPC-lineage cells, the MADM brain tissues were sectioned sagittally in 20 μm thick slices. We stained the MADM brain sections with GFP (1:500), c-Myc (1:200) and Olig2 (1:500). N=4 brains were processed for each time point. We used DAPI to determine the brain regions. For each brain, 5 brain sections were examined and 5 cortical regions within each brain section were imaged. Therefore, 25 images were collected from each mouse brain. Each image was taken by a 20x objective with 1x digital zoom by 2 μm optical sectioning (scanning depth 6μm). We processed each image in two ways: First, we quantified all $Olig2^+$ cells, including red, green, yellow and non-labeled ones. These data were used to calculate the G/R ratio between green and red $Olig2^+$ cells (see **Figure 4G**) and the percentage of all four labeled cell populations with distinct genotypes (see **Figure 4D-F**). Second, we calculated the area (mm^2) of each image to determine the density of all $Olig2^+$ cells (see **Figure 4H**). These values were averaged from N=4 brains from each genotype and presented as mean ± SEM.

Quantification for the Nestin-Cre^{ER} mouse models in Figure S7

To determine the distribution of the mutant OPCs (PDGFRα$^+$, tdT$^+$) in distinct brain structures, the mouse brains were sectioned coronally in 20 μm thick sections and stained with PDGFRα antibody (1:250). The live fluorescence of tdTomato was strong enough. Therefore, no DsRed antibody was used to enhance the signal. We used DAPI to determine brain anatomical structures and defined 10 brain regions covering major representative brain structures in both the gray and the white matter (see **Figure S7C**), which include the olfactory bulb (two random fields), the cerebral cortex (three random fields), the corpus callosum (two random fields, one is next to the SVZ and the other was in the midline), the lateral-SVZ, the striatum and the ventral pallidum. Each image was taken by a 20x objective with 1x digital zoom by 2μm optical sectioning (scanning depth 6μm). We counted all the PDGFRα$^+$, tdT$^+$ co-stained cells and tdT$^+$ only cells to calculate the percentage of mutant OPCs among all NSC-derived cells (see **Figure S7D**). We calculated the area using the Olympus Fluoview 1000 software. The number was averaged from N=3 brains and presented as mean ± SEM.

Quantification for the Nestin-Cre^{ER} mouse models presented in Figure 5

To measure the proliferation rates of mutant adult NSCs and OPCs, we focused on 2 distinct brain regions: the lateral-SVZ and the corpus callosum next to the SVZ in the coronal brain sections. Brain tissues were sectioned coronally in 20 μm thick sections and stained with BrdU (1:500), DsRed (1:100) and Olig2 (1:500) or Sox9 (1:250). In order to quantify the number of mutant granule cells derived from NSCs, we stained the OB sections with NeuN (1:250). We used DAPI to determine brain regions such as the lateral-SVZ (the region where mutant NSCs reside), the corpus callosum next to SVZ (the region mutant OPCs reside) and the OB (the region NSC-derived granule cells reside). Each image in the SVZ region was

taken by a 40x objective with 2x digital zoom by 2 μm optical sectioning (scanning depth 6μm). Each image in the corpus callosum next to the SVZ and in the OB region was taken by a 20x objective with 1x digital zoom by 2μm optical sectioning (scanning depth 6μm). For each brain, we collected images from 5 sections randomly including 10 images in the lateral-SVZ and the corpus callosum next to the SVZ. We also collected 6 images randomly from the OB (one image per section, 6 sections in total). In the lateral-SVZ, we processed each image in three ways for different purposes: (1) We quantified $BrdU^+$, $DsRed^+$ and $Sox9^+$ co-stained cells and $DsRed^+$, $Sox9^+$ co-stained cells. These data were used to calculate the proliferation rate of labeled mutant/WT NSCs (see **Figure 5C**). (2) We quantified the number of $DsRed^+$, $Sox9^+$ cells within the SVZ and the area scanned (mm^2) to calculate the density of mutant NSCs with distinct genotypes (see **Figure 5D**). (3) We counted the number of all $BrdU^+$ cells regardless of whether or not they were tdT^+. This quantification was used to calculate the density of all proliferating cells in the SVZ (see **Figure 5E**). In the corpus callosum next to the SVZ we processed each image in two ways: (1) We quantified $BrdU^+$, $DsRed^+$, $Olig2^+$ co-stained cells and $DsRed^+$, $Olig2^+$ co-stained cells to calculate the proliferation rate of tdTomato labeled OPCs (see **Figure 5G**). (2) We quantified the number of $DsRed^+$, $Olig2^+$ co-stained cells per area (mm^2). This number was used to calculate the density of mutant OPCs in the scanned region (see **Figure 5H**). (3) In the OB region, we quantified the number of $DsRed^+$, $NeuN^+$ co-stained cells and all $NeuN^+$ cells respectively. These numbers were used to calculate the percentage of mutant NSC-derived granule cells among all neurons in the OB (see **Figure 5F**). We calculated the area with the Olympus Fluoview 1000 software. N=4 mice for all groups except for the CKO-Nestin-Cre^{ER} group at 43dpi in **Figure 5C-E, 5G** and **5H**, where N=3. Data presented as mean ± SEM.

Quantification in Figure 5L

Those cells were fixed and stained with primary antibodies such as PDGFRα (1:250), Nestin (1:1000) and Olig2 (1:500) as described above. For each well, we collected over 9 images randomly, each image was taken by a 10x objective with 1x digital zoom by 2μm optical sectioning (scanning depth>= 6μm). We counted all tdT^+, tdT^+ and $PDGFR\alpha^+$, tdT^+ and $Nestin^+$ cells in those images. The relative percentage of these sub-populations were calculated based on these counting. These values were averaged from N=3 (Nestin) / 6 (PDGFRα) wells and presented as mean ±SEM.

Quantification of PDX models in Figure 6P-Q

Mouse brain tissues were sectioned in 20 μm thick sections and stained with GFP (1:500), PhiYFP (1:10,000) and Olig2 (1:500) (for **Figure 6P-Q**). For samples in **Figure 6P** and **6Q**, we collected all the brain sections that contained GFP^+ and $PhiYFP^+$ tumor cells. We processed each image in two ways: (1) We quantified the number of all GFP^+ and $PhiYFP^+$ tumor cells from each brain to calculate the ratio of GFP^+ to $PhiYFP^+$ cells (see **Figure 6P**). (2) Second, we quantified $Olig2^+$ and GFP^+ cells to calculate the percentage of $Olig2^+$ cells among all GFP^+ cells (see **Figure 6Q**). These values were averaged (N=3 for each group) and presented as mean ± SEM.

Quantification of human glioma samples in Figure S2D

Human glioma tissues were sectioned in 15 μm thick slices and stained with OPC linage markers (such as Olig2, 1:500; PDGFRα, 1:250; Sox10, 1:50), astrocyte marker (GFAP; 1:800), stem cell marker Sox2 (1:500) and proliferation marker Ki67 (1:400). (1) We quantified $Ki67^+$, $Olig2^+$, $PDGFR\alpha^+$, $Sox10^+$, $Sox2^+$, $GFAP^+$ cells respectively. These numbers were used to calculate the percentage of the cells expressing these markers among all $DAPI^+$ cells (see **Figure S2D, Supporting Information**). The number from each sample

was the average from 3-12 imaged regions. (2) We quantified Olig2$^+$, PDGFRα^+, Sox10$^+$, Sox2$^+$, GFAP$^+$ cells among all Ki67$^+$ cells and used these numbers to calculate the percentage of the cells expressing these markers among all proliferating cells. Each number was the average from 7-20 imaged regions. (3) We quantified Ki67$^+$, Olig2$^+$, Sox2$^+$, GFAP$^+$ cells respectively among all PDGFRα^+ cells. This quantification was used to calculate the percentage of four kinds of cells among all tumor OPCs. Each number was the average from 7-20 imaged regions.

Quantification of human glioma samples in Figure S8D

Human glioma tissues were sectioned in 15 μm thick slices. To quantify pIGF1R$^+$ cells in **Figure S8D**, we stained the glioma tissue sections with pIGF1R (1:200), PDGFRα (1:250), Ki67 (1:400) and DAPI (N=5 for each grading, see **Table S2** for the information of all patients, **Supporting Information**). The adjacent section stained with secondary antibodies only were always available as the negative control. Each image was taken by a 40x objective with 2x digital zoom by 1 μm optical sectioning (scanning depth 3μm). For each tumor sample, we collected 10 images randomly from 10 sections. Data presented as mean ± SEM.

Quantification of PDX models in Figure 8J

Mouse brain tissues were sectioned in 20 μm thick sections and stained with Ki67 (1:500), Human nuclei (hNA, 1:250) and Olig2 (1:500) (for **Figure 8J**). Each image was taken by a 20x objective with 2x digital zoom by 2μm optical sectioning (scanning depth 6 μm). We processed each image in two ways: (1) we quantified the number of all Olig2$^+$, hNA$^+$ and Ki67$^+$ tumor cells from each brain to calculate the percentage of hNA$^+$, Olig2$^+$ and Ki67$^+$ cells among all hNA$^+$ and Ki67$^+$ cells. (2) Second, we quantified hNA$^+$, Olig2$^+$ and hNA$^+$ cells to

calculate the percentage of Olig2^{+} cells among all hNA^{+} cells. These values were averaged (N=6 for each group) and presented as mean ± SEM.
